# Plasma membrane phylloquinone biosynthesis in nonphotosynthetic parasitic plants

**DOI:** 10.1093/plphys/kiab031

**Published:** 2021-01-30

**Authors:** Xi Gu, Ing-Gin Chen, Scott A Harding, Batbayar Nyamdari, Maria A Ortega, Kristen Clermont, James H Westwood, Chung-Jui Tsai

**Affiliations:** 1 Institute of Bioinformatics, University of Georgia, Athens, GA 30602, USA; 2 School of Forestry and Natural Resources, University of Georgia, Athens, GA 30602, USA; 3 Department of Genetics, University of Georgia, Athens, GA 30602, USA; 4 Department of Plant Biology, University of Georgia, Athens, GA 30602, USA; 5 School of Plant and Environmental Sciences, Virginia Polytechnic Institute and State University, Blacksburg, VA 24061, USA

## Abstract

Nonphotosynthetic holoparasites exploit flexible targeting of phylloquinone biosynthesis to facilitate plasma membrane redox signaling. Phylloquinone is a lipophilic naphthoquinone found predominantly in chloroplasts and best known for its function in photosystem I electron transport and disulfide bridge formation of photosystem II subunits. Phylloquinone has also been detected in plasma membrane (PM) preparations of heterotrophic tissues with potential transmembrane redox function, but the molecular basis for this noncanonical pathway is unknown. Here, we provide evidence of PM phylloquinone biosynthesis in a nonphotosynthetic holoparasite *Phelipanche aegyptiaca*. A nonphotosynthetic and nonplastidial role for phylloquinone is supported by transcription of phylloquinone biosynthetic genes during seed germination and haustorium development, by PM-localization of alternative terminal enzymes, and by detection of phylloquinone in germinated seeds. Comparative gene network analysis with photosynthetically competent parasites revealed a bias of *P. aegyptiaca* phylloquinone genes toward coexpression with oxidoreductases involved in PM electron transport. Genes encoding the PM phylloquinone pathway are also present in several photoautotrophic taxa of Asterids, suggesting an ancient origin of multifunctionality. Our findings suggest that nonphotosynthetic holoparasites exploit alternative targeting of phylloquinone for transmembrane redox signaling associated with parasitism.

## Introduction

Phylloquinone (vitamin K1) is a membrane-bound naphthoquinone derivative known to function as an essential electron acceptor in photosystem I (PSI; Brettel et al., 1986). Phylloquinone also serves as an electron carrier for protein disulfide bond formation crucial for PSII assembly (Furt et al., 2010; Karamoko et al., 2011; Lu et al., 2013). Accordingly, phylloquinone is found predominantly in thylakoids, and most phylloquinone-deficient Arabidopsis (*Arabidopsis thaliana*) mutants are seedling-lethal or growth-impaired (reviewed in Gilles et al., 2016). A sizable phylloquinone pool is stored in plastoglobuli attached to thylakoid membranes (Lohmann et al., 2006; Eugeni Piller et al., 2012). A small portion of leaf phylloquinone is present as fully reduced quinol, with potential redox function during senescence or dark growth (Oostende et al., 2008).

The eubacterial cognate menaquinone (vitamin K2) functions in respiratory electron transport across the cell membrane (Nowicka and Kruk, 2010). A similar role for phylloquinone in plant plasma membrane (PM) electron transport has also been proposed, and phylloquinone has been detected in PM preparations of maize (*Zea mays*) roots (Lüthje et al., 1998; Lochner et al., 2003). UV-irradiation of cultured carrot (*Daucus carota*) cells destroyed phylloquinone, and blocked transmembrane electron transport until restoration by phylloquinone feeding (Barr et al., 1992). The PM redox activities can be inhibited by phylloquinone antagonists, dicumarol and warfarin, whereas applications of menadione (vitamin K3) or phylloquinone restore transmembrane electron flow (Döring et al., 1992a, 1992b; Lüthje et al., 1992). Despite these early reports, however, molecular evidence that directly supports PM-targeting of phylloquinone remains elusive.

**Figure 1:**
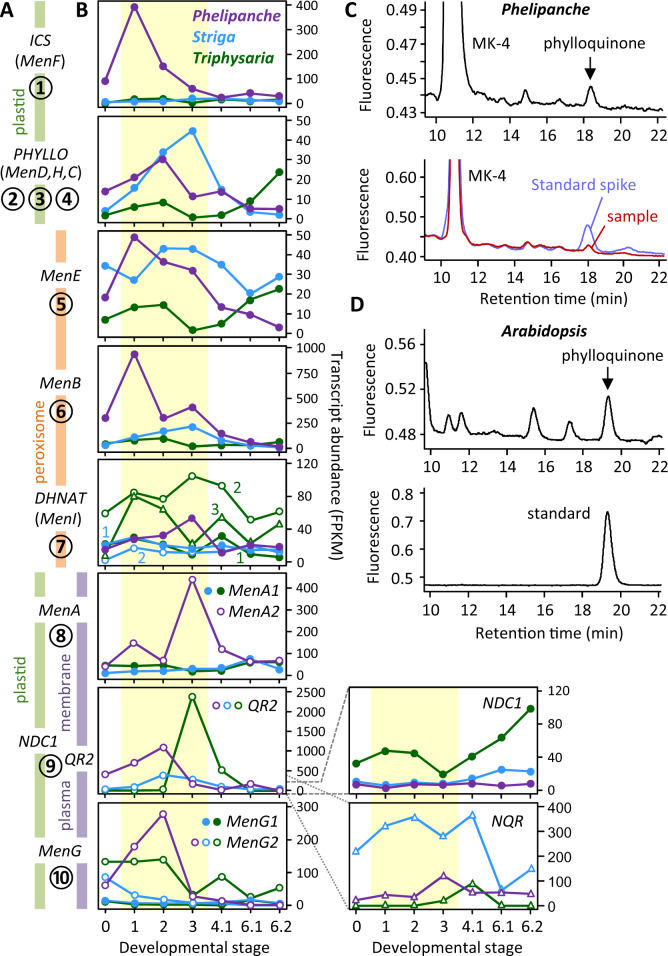
Phylloquinone biosynthesis in parasitic plants. A, A simplified phylloquinone biosynthetic pathway from top to bottom as numbered and color-coded by their predicted subcellular localization. B, Expression profiles of phylloquinone genes during parasitic plant development. Gene order is the same as in (A), and *Y*-axis is shown on the right. Developmental stages are as reported (Yang et al., 2015): 0, imbibed seeds; 1, germinated seeds/radicles; 2, HIF-treated seedlings; 3, haustoria, pre-vascular connection; 4.1, haustoria, post-vascular connection; 6.1, leaves/stems; 6.2, floral buds. Data from alternative gene candidates for the penultimate step are shown on the lower right panels, with grey dashed/dotted lines denoting the scale. C, HPLC detection of phylloquinone in germinated *P. aegyptiaca* seeds (top panel), and with spiked authentic standard (lower panel). Menaquinone (MK-4) was included as a reference. D, HPLC detection of phylloquinone in *A. thaliana* seed (top panel), and with authentic standard (lower panel)

The phylloquinone biosynthetic pathway has an endosymbiotic origin, comprising a series of “Men” proteins named after their eubacterial homologs in the menaquinone biosynthesis operon (Figure 1). A notable exception is the penultimate enzyme (step 9, Figure 1A) of the flavin-containing NAD(P)H quinone oxidoreductase (FQR/NQR/QR) family recently shown to be necessary for phylloquinone biosynthesis in plants and cyanobacteria (Eugeni Piller et al., 2011; Fatihi et al., 2015). The corresponding enzyme in *A. thaliana*, type II NAD(P)H dehydrogenase C1 (NDC1), was first identified as a mitochondrial respiratory chain component (Michalecka et al., 2003), but later was also found to target to the chloroplast (Carrie et al., 2008), with additional functions in plastoquinone reduction and redox cycling of α-tocopherols in plastoglobuli (Eugeni Piller et al., 2011). Intracellular compartmentalization is a hallmark of phylloquinone biosynthesis, with the early (steps 1–4, Figure 1A) and late pathway steps (8–10) occurring in chloroplasts, and the intermediate steps (5–7) in peroxisomes (Babujee et al., 2010). Even within the chloroplast, the three terminal steps shuttle between envelope membranes (MenA, Schultz et al., 1981), plastoglobuli (NDC1, Eugeni Piller et al., 2011), and thylakoid membranes (MenG, Kaiping et al., 1984), highlighting the complex trafficking involved in this pathway.

We have observed in multiple photosynthetic taxa that phylloquinone-specific genes such as *MenA* and *MenG* have measurable expression in heterotrophic tissues where photosynthetic genes are barely detected. To ascertain a noncanonical phylloquinone pathway, we exploited parasitic plants as a photosynthesis-free study system. Among angiosperm parasite families, only the Orobanchaceae contains species that span the full spectrum of photosynthetic capacities, and for which rich transcriptomic resources are available (Westwood et al., 2010; Yang et al., 2015). Of particular interest are obligate holoparasites, such as *Phelipanche aegyptiaca*, that are devoid of photosynthetic activity and obtain all of their carbon from their hosts. In contrast, obligate (e.g. *Striga hermonthica*) and facultative (e.g. *Triphysaria versicolor*) hemiparasites are partially or fully photosynthetic. Here, we report on the biosynthesis and PM targeting of phylloquinone in the nonphotosynthetic *P. aegyptiaca*. Gene network analysis revealed a strong link between phylloquinone and cellular oxidation–reduction processes implicated in parasitic invasion and haustorium development. We propose that parasitic plants exploit alternative phylloquinone targeting for PM redox regulation associated with parasitism.

## Results

### *Phelipanche aegyptiaca* contains the full complement of phylloquinone biosynthetic genes

Phylloquinone pathway protein sequences of *Mimulus guttatus*, a photoautotroph from Phrymaceae sister to Orobanchaceae, were searched against transcript assemblies available from the Parasitic Plant Genome Project (Yang et al., 2015). Full-length coding sequences were identified for *PaICS* and *PaMenE* genes in *P. aegyptiaca*, along with partial assemblies of other phylloquinone pathway genes. Fragmented transcripts may represent nonfunctional relics of genes undergoing degeneration or may reflect technical limitations of *de novo* assembly that prevented identification of the full complement of phylloquinone biosynthetic genes. The recovery of full-length *PaICS* and *PaMenE* transcripts strongly favored the latter scenario.

We addressed the fragmented assembly challenge by developing a pipeline called parallelized local assembly of sequences (PLAS) that combines reference-guided mapping (against the *M. guttatus* proteome in this case) with iterative *de novo* assembly for transcriptome reconstruction. When applied to the RNA-seq datasets of parasitic plants, we successfully recovered full-length transcripts with intact ORFs for all known phylloquinone genes from the holoparasite (Supplemental Data Set S1). These transcripts were detected at moderate to high levels during *P. aegyptiaca* development, except *PaNDC1*—the latest addition to the pathway—which was poorly expressed throughout the holoparasite (Figure 1B). *PHYLLO*, *MenE* and *DHNAT* (*dihydroxynaphthoate thioesterase*) transcripts were detected at similar levels in the three parasites. In contrast, the expression patterns of *ICS*, *MenB*, *MenA*, and *MenG* differed between *P. aegyptiaca* and its photosynthetic relatives *S. hermonthica* and *T. versicolor*, especially in response to germination stimulants and haustorium-inducing factors (HIFs) during early development (stages 1–3, Figure 1B). However, the apparent lack of *PaNDC1* expression weakened support for a functioning phylloquinone pathway. Alternatively, retention and expression of the other seven phylloquinone pathway genes may point to a different pathway configuration at the penultimate step in the holoparasite.

We therefore sought to substantiate phylloquinone production in *P. aegyptiaca*, focusing on early development prior to host exposure and haustorium initiation to avoid potential contamination from the photosynthetic host. HPLC analysis confirmed the presence of phylloquinone in germinated *P. aegyptiaca* seeds (Figure 1C), with an estimated level of 0.05 ± 0.02 pmol/mg dry weight (*n* = 3). For reference, phylloquinone levels in *A. thaliana* seeds were 0.12 ± 0.03 pmol/mg dry weight (*n* = 3; Figure 1D). The result lent support to phylloquinone biosynthesis in the holoparasite. We next examined other candidates that could participate in the reduction of demethylphylloquinone at the penultimate step (step 9). Besides the multifunctional NDC1 (Fatihi et al., 2015), menadione-reducing activities have been demonstrated for two other groups of evolutionarily conserved QR, one represented by *T. versicolor* TvQR2 (Wrobel et al., 2002) and *A. thaliana* AtFQR1 (Laskowski et al., 2002) of the type IV family (Patridge and Ferry, 2006), and the other by soybean (*Glycine max*) GmNQR of the DT-diaphorase type (Schopfer et al., 2008). *PaNQR* transcripts were detected at moderate levels throughout *P. aegyptiaca* development (Figure 1B), but they were not coexpressed with any phylloquinone pathway genes (see below). In contrast, *PaQR2* was well expressed during early stages of *P. aegyptiaca* development like other phylloquinone genes (Figure 1B). Type IV QR2 has in fact been implicated in reduction of menaquinone and phylloquinone to their respective quinols in the manually curated KEGG (Kyoto Encyclopedia of Genes and Genomes) reference pathways (Kanehisa et al., 2018). The structural similarity between phylloquinone and its demethylated precursor, and the reported substrate promiscuity of the orthologous TvQR2 (Wrobel et al., 2002) lent further support to PaQR2 involvement in the penultimate step of *P. aegyptiaca* phylloquinone biosynthesis.

### Phylloquinone biosynthesis is redirected to the PM in the holoparasite

**Figure 2:**
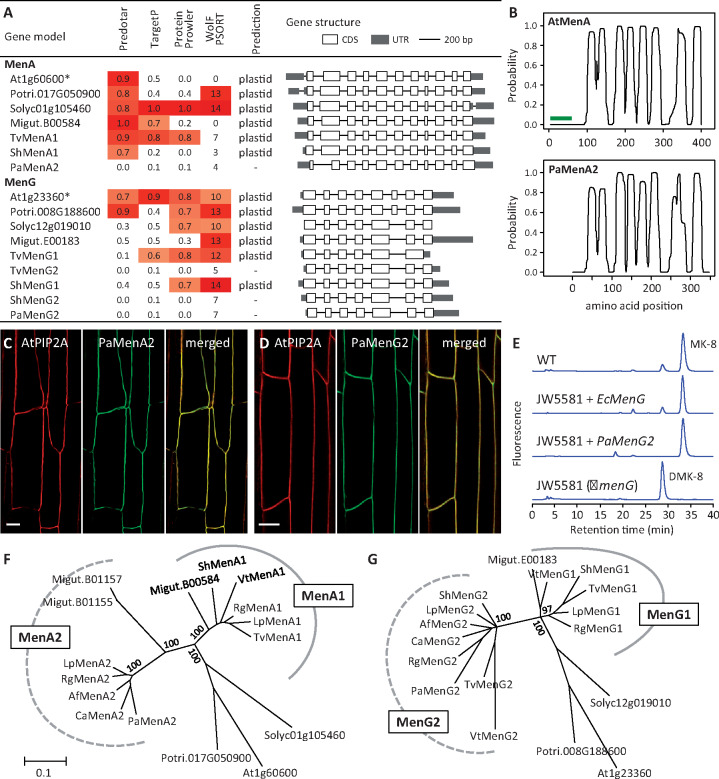
Characterization of MenA and MenG. A, Plastid-targeting prediction of MenA and MenG polypeptides from various species. Heatmaps show prediction strengths above the 50th percentile of each method. Those with a high prediction score from at least one program are deemed potentially plastidial, as not all methods accurately predict experimentally characterized plastidial proteins (asterisks). Introns are not drawn to scale. CDS, coding sequence; UTR, untranslated region. B, Transmembrane domain prediction of AtMenA (green line denoting the transit peptide) and PaMenA2 (shifted *x*-axis for domain alignment). C–D, Confocal images of PaMenA2-GFP (C) and PaMenG2-GFP (D) colocalization with a plasma membrane marker AtPIP2A-mCherry. Scale bars = 20 µm. E, HPLC analysis of *E. coli* ΔmenG mutant strain JW5581 expressing *PaMenG2* or the *EcMenG* control. (D)MK-8, (demethyl)menaquinone-8. F–G, Bayesian phylogeny of MenA and MenG from representative Asterids and two Rosids. Af, *Aphyllon fasciculata*; At, *Arabidopsis thaliana*; Ca, *Conopholis americana*; Lp, *Lindenbergia philippensis*; Migut, *Mimulus guttatus*; Pa, *Phelipanche aegyptiaca*; Potri, *Populus trichocarpa*; Rg, *Rehmannia glutinosa*; Solyc, *Solanum lycopersicum*; Sh, *Striga hermonthica*; Tv, *Triphysaria versicolor*; Vt, *Verbascum thapsus*. Branch support is shown for major nodes. Scale indicates the number of amino acid substitutions per site

Identification of the phylloquinone pathway in *P. aegyptiaca* with vestigial plastids raised the possibility that the biosynthetic enzymes exhibit alternative targeting with nonplastidial function(s). Protein subcellular localization analyses predicted plastid- and peroxisome-targeting of early (PaICS and PaPHYLLO) and intermediate (PaMenE, PaMenB, and PaDHNAT) pathway steps, respectively, similar to their orthologs in photoautotrophic taxa (Supplemental Table S1 and Supplemental Figures S1–S3). However, the predicted polypeptides of late pathway steps PaMenA and PaMenG are truncated at the N-terminus relative to their photoautotrophic orthologs (Figure 2;Supplemental Figures S4–S5) and scored poorly for plastid-targeting with multiple prediction programs (Figure 2A). The N-truncation of PLAS-derived *PaMenA* and *PaMenG* transcripts was independently confirmed by reverse transcription polymerase chain reaction (RT-PCR) cloning and sequencing (GenBank accession numbers MT506520 and MT506521), and by identification of N-truncated orthologs from other species (see below).

PaMenA is predicted as an integral protein with multiple transmembrane helices in a topology similar to that of AtMenA (Figure 2B). The absence of an N-terminal plastid-targeting peptide in PaMenA suggests its localization to other cellular membranes. The penultimate PaQR2, like its photoautotrophic orthologs, also lacks plastid-targeting sequence (Supplemental Figure S6), which contrasts with the alternative NDC1s that are predicted plastidial (Supplemental Table S1). Indeed, PM association has been reported for *A. thaliana*, rice (*Oryza sativa*), and yeast (*Candida albicans*) QR2 orthologs (Marmagne et al., 2004; Natera et al., 2008; Li et al., 2015). These data suggest the post-peroxisomal steps are likely targeted to PM in *P. aegyptiaca*. We generated stably transformed *Nicotiana benthamiana* expressing *35S:PaMenA-GFP* or *35S:PaMenG-GFP* along with a PM marker *35S:AtPIP2A-mCherry* (Nelson et al., 2007). Confocal imaging showed co-localization of PaMenA and PaMenG with the PM marker in transgenic roots (Figure 2, C and D), providing experimental support for alternative targeting of the N-truncated PaMenA and PaMenG in *P. aegyptiaca*.

### The PM pathway has canonical activity and an ancient origin

The absence of the N-terminal transit signal is not expected to impact (mature) enzyme catalysis. Using PaMenG as a test case, we performed complementation experiments with the *Escherichia coli* Δ*menG* mutant strain JW5581 (Baba et al., 2006). The *E. coli* MenG, also called UbiE, is a dual C-methyltransferase involved in both menaquinone and ubiquinone biosynthesis, and its mutation leads to over-accumulation of demethylmenaquinone (Lee et al., 1997). Constitutive expression of *PaMenG* in the mutant strain restored menaquinone production similar to the *EcMenG*-complemented control (Figure 2E). The data provide biochemical evidence for canonical activity of the N-truncated PaMenG.

Interestingly, distinct *MenG1* and *MenG2* genes encoding long (plastidial) and short (PM) isoforms, respectively, are present in both *S. hermonthica* and *T. versicolor* (Figure 2A), suggesting that the PM phylloquinone pathway may have evolved before the transition to parasitism. To strengthen this finding, we mined the One Thousand Plant Transcriptomes (1KP) database (Leebens-Mack et al., 2019) and identified several photoautotrophic species—all from Lamiales—that harbor both long and short isoforms of MenA and/or MenG, including *Lindenbergia philippensis*, *Rehmannia glutinosa*, and *Verbascum thapsus*. In contrast, only N-truncated short isoforms were found in two other Orobanchaceae holoparasites, *Aphyllon* (syn. *Orobanche*) *fasciculata* and *Conopholis americana* (Supplemental Figures S4–S5). Phylogenetic analysis clustered the plastidial and PM isoforms into distinct groups for both MenA and MenG (Figure 2, F and G), suggesting their origin from lineage-specific duplication events (hereafter, the PM-localized short isoforms are referred to as MenA2 and MenG2). Of note, transcripts encoding PM-targeted MenG2 were detected at higher levels than those encoding plastidial MenG1 in both *S. hermonthica* and *T. versicolor* (Figure 1B), suggesting divergent regulation of the two MenG isoforms in photosynthetic hemiparasites.

### Photosynthetic and nonphotosynthetic parasites exhibit distinct phylloquinone gene coexpression patterns

**Figure 3:**
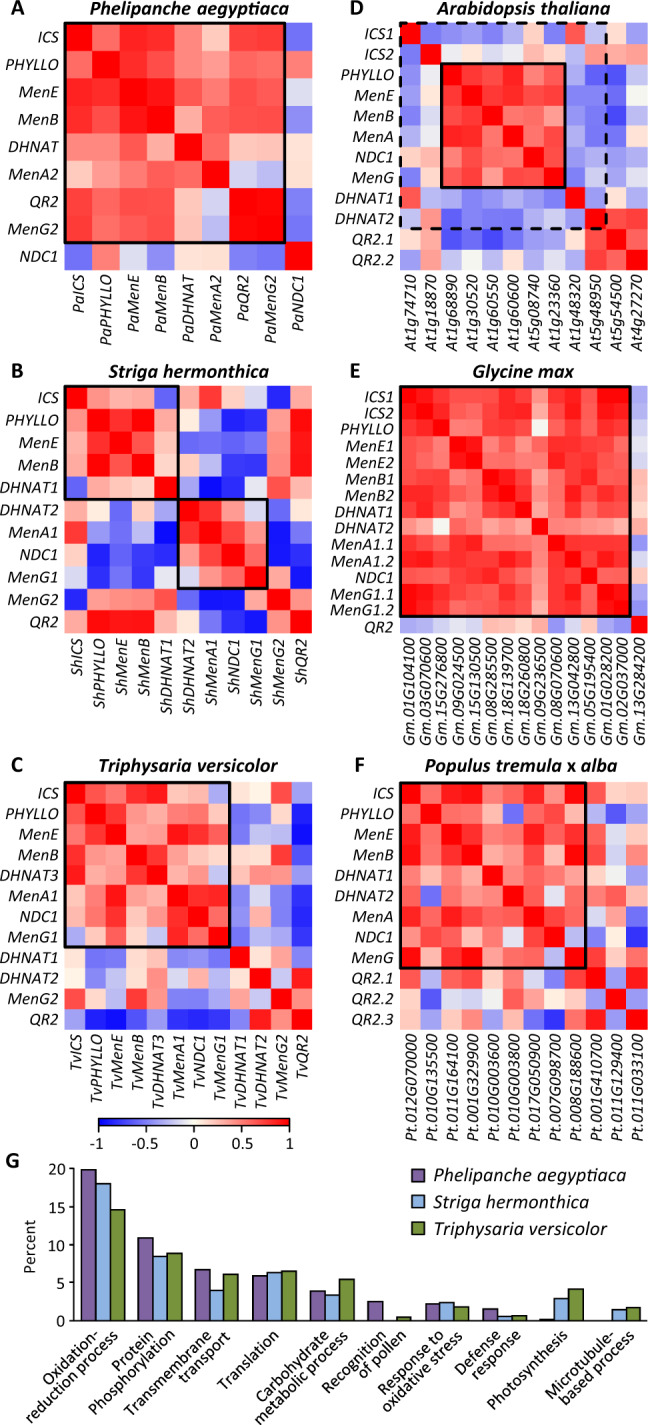
Coexpression of phylloquinone genes. A–F, Coexpression patterns among phylloquinone biosynthetic genes, including multifunctional *NDC1* and *QR2*, based on Gini correlation coefficient. Relevant genes or gene members involved in phylloquinone biosynthesis are boxed. The exceptions are *A. thaliana AtICSs* involved in salicylic acid biosynthesis for defense and *AtDHNATs* in peroxisomal β-oxidation (dashed box), besides phylloquinone biosynthesis. The corresponding *Arabidopsis*, *Glycine*, and *Populus* gene models are shown on the *x*-axis with shortened prefix for soybean (Gm = Glyma) and poplar (Pt = Potri). G, GO enrichments of phylloquinone-coexpressed genes defined as the union set of the top 500 most highly correlated transcripts for each phylloquinone gene. Only the top 10 categories are shown. Similar results were obtained using Gini correlation coefficient ≥0.8 to extract phylloquinone-coexpressed genes

We detected high levels of coexpression among phylloquinone genes in the holoparasites, reminiscent of the patterns observed in photosynthetic taxa, including *T. versicolor* (Figure 3) [except for *A. thaliana AtICSs* and *AtDHNATs* involved in salicylic acid biosynthesis (Garcion et al., 2008) and peroxisomal β-oxidation (Cassin-Ross and Hu, 2014), respectively]. Interestingly, early- and late-pathway genes showed distinct coexpression patterns in the obligate hemiparasite *S. hermonthica*, perhaps indicative of dual involvement of those genes in plastidial and nonplastidial functions. To shed light on PM-phylloquinone functions, we extracted the top 500 most highly correlated transcripts for each phylloquinone gene except the alternative *QR2* and *NDC1.* The union set contained 2,447, 3,677, and 3,930 unique transcripts for *P. aegyptiaca*, *S. hermonthica*, and *T. versicolor*, respectively. Interestingly, *QR2* but not *NDC1* was captured in all three networks. The smaller *P. aegyptiaca* gene set is consistent with stronger coexpression of phylloquinone genes when compared to *S. hermonthica* and *T. versicolor* (Figure 3, A–C). Subsets of gene ontology (GO)-annotated (biological process) transcripts (645, 1,199, and 1,173 for *P. aegyptiaca*, *S. hermonthica*, and *T. versicolor*, respectively) were then subjected to functional enrichment analysis. Transcripts associated with “photosynthesis” comprised 3%–4% GO-annotated transcripts in photosynthetic parasites but were negligible in *P. aegyptiaca* (Figure 3G). In contrast, transcripts associated with “oxidation–reduction process”, “protein phosphorylation”, and “defense response” were more enriched in *P. aegyptiaca* relative to *S. hermonthica* and *T. versicolor* (Figure 3G).

**Figure 4:**
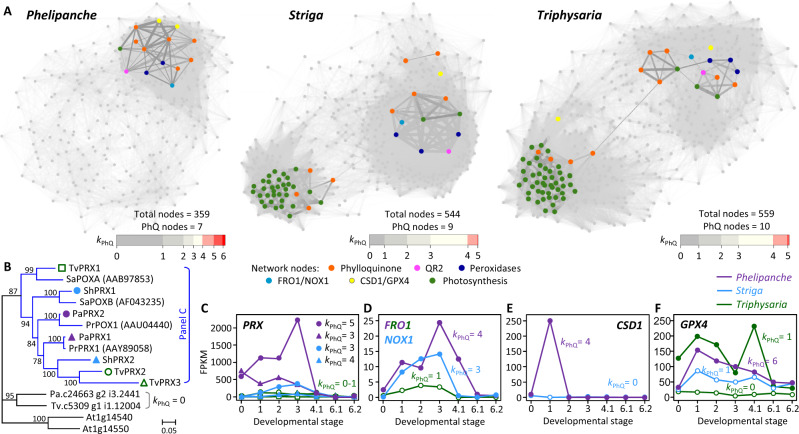
Phylloquinone gene coexpression networks. A, Network visualization of the three parasitic plants. Edge thickness reflects the coexpression strength. Key gene nodes are color-coded by pathway or family, and *QR2* is colored differently from the other phylloquinone genes (*NDC1* was not captured in any of the networks). Horizontal bars depict the distribution of nodes according to their connectivity with phylloquinone genes (*k*_PhQ_). B, Bayesian phylogeny of peroxidases (PRX). Orthologs of experimentally characterized PRXs are color-coded by species in the blue clade. Scale indicates the number of amino acid substitutions per site. C–F, Expression profiles of *PRX* (C), *FRO1/NOX1* (D), *CSD1* (E), and *GPX4* (F) orthologs. Solid symbols denote phylloquinone-coexpressed genes (*k*_PhQ_ ≥3), and others are shown in open symbols. Developmental stages are the same as in Figure 1

We focused on transcripts assigned to oxidation–reduction, defense, and photosynthesis GO terms for coexpression network analysis. Inclusion of orthologs from all three parasites resulted in 359, 544, and 559 nonredundant transcripts from *P. aegyptiaca*, *S. hermonthica*, and *T. versicolor*, respectively (Supplemental Data Set S2). Network visualization of coexpression patterns revealed striking differences (Figure 4). Two dense modules were detected for photosynthetic *S. hermonthica* and *T. versicolor*; one enriched with photosynthesis genes (Figure 4A, green nodes) and the other containing known parasitism genes (blue, magenta, and cyan nodes, see below). However, only one dense module containing parasitism genes was detected for the holoparasite. The phylloquinone genes (orange nodes) were highly interconnected with parasitism genes in the *P. aegyptiaca* network but were scattered over the two modules in *S. hermonthica* and *T. versicolor* networks, presumably reflecting dual functionality in these taxa. We ranked genes by the number of edges they shared with phylloquinone genes (referred to as *k*_PhQ_) in each network and observed a strong enrichment of phylloquinone-interconnected genes in the smaller *P. aegyptiaca* network. More than 23% of *P. aegyptiaca* nodes had a *k*_PhQ_ =4–6 (i.e. connected with a majority of the seven phylloquinone genes). However, less than 3% of the *S. hermonthica* and *T. versicolor* nodes met the same criterion (*k*_PhQ_ ≥5 of 9–10 phylloquinone genes), and only 10% and 15% of their respective nodes had a *k*_PhQ_ ≥4 (Figure 4A, *k*_PhQ_ bars).

### *Phelipanche aegyptiaca* phylloquinone gene networks associate parasitism with PM redox signaling

Several oxidoreductases known to be involved in parasitism were captured in the phylloquinone gene network of *P. aegyptiaca*. Root-specific apoplastic/secretory peroxidases (PRXs or POXs), including *S. asiatica* SaPOXA and SaPOXB and *P. ramosa* PrPOX1 and PrPRX1, have established roles in haustorium induction via generation of reactive oxygen species (ROS) and oxidation of host cell wall-derived phenolics and quinones, such as 2,6-dimethoxy-1,4-benzoquinone (DMBQ), as HIFs (Kim et al., 1998; González-Verdejo et al., 2006; Veronesi et al., 2007). The *PRX* orthologs (Figure 4B, blue clade) were abundantly expressed in *P. aegyptiaca* throughout seed germination and haustorium development (Figure 4C). The phylloquinone-coexpression strength (*k*_PhQ_) was highest in *P. aegyptiaca*, followed by *S. hermonthica*, but was not observed for *T. versicolor PRXs*, or for homologs in the neighboring clade of the phylogenetic tree (Figure 4, B and C). Thus, the phylloquinone-coexpression strength of the secretory PRXs appears to be positively correlated with parasitism.

Also implicated in parasitism are QR1 and QR2, depending on the species. QR1 is a chloroplast envelope protein of the ζ-crystalline type NAD(P)H oxidoreductase family involved in haustorium development of *T. versicolor* (Bandaranayake et al., 2010). However, *QR2* but not *QR1* is responsive to HIFs in *S. asiatica* and *Phtheirospermum japonicum* (Ishida et al., 2016; Liang et al., 2016), similar to the patterns observed for *P. aegyptiaca* and *S. hermonthica* (Figure 1B). *QR1* was not captured in any of the phylloquinone subnetworks, whereas the phylloquinone-coexpression strength of *QR2* was highest in *P. aegyptiaca* (*k*_PhQ_ =4), followed by *S. hermonthica* (*k*_PhQ_ =3) and *T. versicolor* (*k*_PhQ_ =1; Figure 4A), similar to the secretory PRXs. These results suggest a link between phylloquinone biosynthesis and parasitism.

We next searched the *P. aegyptiaca* phylloquinone subnetwork for redox proteins known to participate in transmembrane electron transport (Bérczi and Møller, 2000; Keyes et al., 2000). Several flavocytochrome *b* superfamily proteins catalyze PM electron transport from cytosolic NAD(P)H to apoplastic acceptors, including NAD(P)H oxidases (NOX, also known as respiratory burst oxidase homologs) and ferric reductase oxidases (FRO; Bérczi and Møller, 2000; Jain et al., 2014). The *P. aegyptiaca* transcriptome lacked *NOX*, but contained an *FRO* (*PaFRO1*) orthologous to *AtFRO4/AtFRO5* tandem duplicates that function as Cu-specific reductases at the root surface (Bernal et al., 2012). *PaFRO1* transcript levels were highest in prehaustorial and haustorial tissues (Figure 4D), suggesting potential involvement in the PM redox system there. Interestingly, *S. hermonthica* harbors a *NOX*, but not *FRO*, perhaps indicative of lineage-specific transcriptome rewiring. Its counterpart in *S. asiatica* (*SaNOX1*) is indeed root-specific and HIF-responsive (Liang et al., 2016).

Electron transport chains are major sources of ROS, which are tightly controlled by antioxidant systems, such as Cu/Zn superoxide dismutases (Cu/Zn-SOD or CSD) and glutathione peroxidases (GPX; Mittler et al., 2004; Margis et al., 2008). *P. aegyptiaca PaCSD1* (*k*_PhQ_ =4) and *PaGPX4* (*k*_PhQ_ =6) were highly coexpressed with phylloquinone genes (Figure 4, E and F). CSD1 is a known leaderless secretory protein frequently detected in apoplastic/secreted proteomes (Cheng et al., 2009; Pechanova et al., 2010), while GPX4 orthologs in *A. thaliana* (*AtGPX4/5* genome duplicates) were recently shown to be PM-anchored based on redox-sensitive GFP fusions (Attacha et al., 2017). Thus, besides the secretory PaPRXs, PaCSD1, and PaGPX4 may also participate in apoplastic redox modulation in the holoparasite. In contrast, the phylloquinone genes of photosynthetic parasites were coexpressed with gene family members specifically targeted to plastids (orthologs of AtCSD2 and/or Fe-SOD AtFSD2/3), mitochondria (AtGPX6) or cytosol (AtGPX8; Supplemental Data Set S2). The differential coordination between *P. aegyptiaca* and its photosynthetic relatives with distinct subcellular antioxidant systems is consistent with their contrasting photosynthetic capabilities, and with a prominent role of phylloquinone in PM redox homeostasis of the nonphotosynthetic holoparasite.

## Discussion

### An ancient origin of flexible phylloquinone biosynthesis and targeting

**Figure 5:**
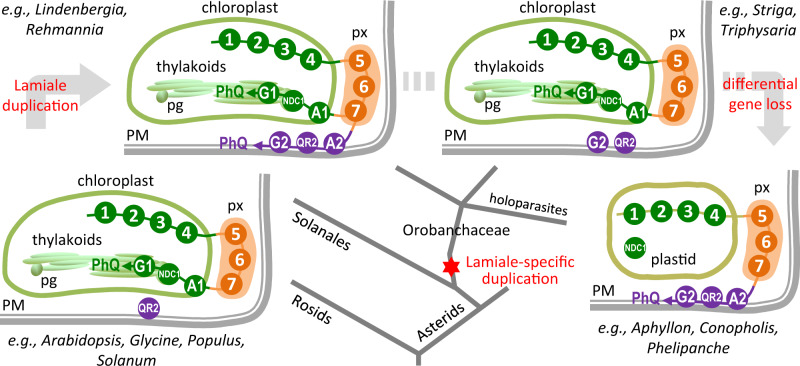
Evolutionary changes in subcellular localization of phylloquinone biosynthesis. Conserved early- and mid-pathway steps are shown as circled numbers (1–7, see Figure 1) and late pathway steps MenA and MenG are abbreviated as A1/A2 and G1/G2, respectively. Circle colors denote different subcellular compartments: green, plastid; orange, peroxisome (px); purple, PM. Branches of the simplified eudicot phylogeny in the middle point to corresponding illustrations of changing pathway organization with representative species indicated. Clockwise from lower left, exclusively plastidial late steps in rosids and some asterids such as solanales; top left, dual plastidial and plasma membrane targeting in certain photosynthetic Orobanchaceae, attributable to lamiale-specific duplication of MenA and MenG. Differential losses of *MenA* and *MenG* genes in other photosynthetic, hemiparasitic (top right) and holoparasitic (lower right) lineages, with the latter exclusively PM targeting. pg, plastoglobule

We present multiple lines of evidence to support a functional phylloquinone biosynthetic pathway in the nonphotosynthetic *P. aegyptiaca*. Our data further revealed the post-peroxisomal steps as key to flexible phylloquinone biosynthesis (Figure 5). Alternative targeting to the PM is facilitated by N-truncated MenA2 and MenG2 in conjunction with QR2. QR2 has previously been shown to function in mitigating oxidative stress in bacteria, yeast, and plants (Laskowski et al., 2002; Wrobel et al., 2002; Patridge and Ferry, 2006; Li et al., 2015). In plants, *QR2s* exhibit root-biased expression and are highly responsive to auxins and quinones, including HIFs in the case of parasitic plants (Matvienko et al., 2001; Laskowski et al., 2002; Ishida et al., 2017; Figure 1B). Specifically, RNAi-silencing of *QR2* in *Phtheirospermum japonicum* significantly reduced haustorium formation (Ishida et al., 2017). The proposed QR2 involvement in PM phylloquinone biosynthesis thus represents another example of a multi-functional NAD(P)H oxidoreductase for the penultimate step, analogous to NDC1 in plastidial phylloquinone biosynthesis (Fatihi et al., 2015).

PM-localized MenA2 and MenG2 likely arose from their plastidial counterparts via gene duplication in the common ancestor of Lamiales (Figure 5). Both *MenA* and *MenG* duplicates are present in *Lindenbergia* and *Rehmannia*, two nonparasitic genera sister to the parasitic Orobanchaceae (McNeal Joel et al., 2013). However, the evolutionary fate of the duplicates varied among parasitic lineages with different photosynthetic capabilities (Figure 5). The holoparasitic *P. aegyptiaca*, *Aphyllon fasciculata*, and *Conopholis americana* have dispensed with *MenA1* and *MenG1* along with the photosynthetic machinery, retaining only *MenA2* and *MenG2*. On the other hand, the photosynthetic *T. versicolor* and *S. hermonthica* have lost *MenA2* but retain the *MenG1/2* duplicate, as confirmed in the recently released *S. asiatica* genome (Yoshida et al., 2019). In both *T. versicolor* and *S. hermonthica*, the PM-targeted MenG2 became the dominantly expressed isoform over the plastidial MenG1 (Figure 1B), which was subsequently lost in multiple holoparasites. Interestingly, in both cases, *MenG2* but not *MenG1* exhibited strong positive coexpression with *QR2* (Figure 2, B and C). The data suggest that expression divergence of the two phylloquinone pathways in the hemiparasites predated emergence of holoparasites.

An outstanding question in PM phylloquinone biosynthesis regards the co-substrate for MenA-mediated prenylation. In photoautotrophs, the prenyl precursor of plastidial prenylquinones is phytyl-diphosphate derived from geranylgeranyl-diphosphate via *de novo* synthesis or from phytol released upon chlorophyll degradation (Keller et al., 1998; Ischebeck et al., 2006). In *A. thaliana*, the chlorophyll salvage pathway is indispensable for leaf tocopherol and phylloquinone synthesis, but seeds depend on a distinct phytol pool of as-yet-undefined origin (Valentin et al., 2006; Zhang et al., 2014; Wang et al., 2017). *Phelipanche* and related *Orobanche* seeds are filled with protein and oil bodies (Joel et al., 2012), and lipids are the main storage reserve comprising up to 30% of dry seed mass (Bar-Nun and Mayer, 2002). Whether the prenyl moiety of PM phylloquinone is supplied by the cytosolic isoprenoid pathway or via other mechanisms requires further investigation.

### Phylloquinone involvement in PM redox modulation

Membrane localization is a defining feature of lipid-soluble vitamin K in bacteria, animals and plants (Nowicka and Kruk, 2010). Retention and redirection of the phylloquinone pathway to PM in the nonphotosynthetic *Phelipanche* suggest a role analogous to the plastidial counterpart in thylakoid membranes or to menaquinone in bacterial membranes. Our data thus offer molecular support for the long-proposed involvement of phylloquinone in PM electron transport of heterotrophic tissues (Döring et al., 1998; Lüthje et al., 1998).

The *P. aegyptiaca* phylloquinone coexpression network comprised redox proteins typical of a transmembrane electron transport chain, consistent with a role in PM redox function. The early stages of the parasitic lifecycle can be characterized as a continuum of oxidative events, from activation and perception of host HIFs, to induction of haustoria for host penetration and vascular connection (Keyes et al., 2000; Fernández-Aparicio et al., 2016). Redox modulation is also integral to normal growth and development, as well as defense and counterdefense in the case of host–parasite interactions (Kim et al., 1998; Mittler et al., 2004). Redox-active phylloquinone in the PM may function in all of these processes, but most likely in ways specifically associated with haustorium formation based on network inference and coexpression with known parasitism genes presented herein. This interpretation is consistent with previous studies where the vitamin K antagonist dicumarol reduced haustorium development in *T. versicolor* (Matvienko et al., 2001; Wrobel et al., 2002).

Recently, two studies independently identified an *A. thaliana* PM-localized leucine-rich-repeat receptor kinase, named hydrogen peroxide-induced Ca^2+^ increases1 (HPCA1) or cannot respond to DMBQ1 (CARD1), as a sensor for hydrogen peroxide as well as quinones in Ca^2+^-dependent signal transduction (Laohavisit et al., 2020; Wu et al., 2020). AtCARD1 perceives various quinone molecules and has lower *K*_m_ values for menadione and 1,4-naphthoquinone than DMBQ (Laohavisit et al., 2020). Its parasitic orthologs from *Phtheirospermum japonicum* and *S. asiatica* rescued *A. thaliana* mutants in quinone-mediated Ca^2+^ signaling (Laohavisit et al., 2020), suggesting a conserved quinone/ROS signaling mechanism that has been exploited for parasitism. These previously unannotated orthologs were not included in our network analysis, but of the two *PaCARD* transcripts we recovered, one exhibited strong coexpression with phylloquinone genes. It is tempting to speculate that phylloquinone functions as an endogenous quinone signal to bolster HIF-induced CARD signaling for haustorium formation. Phylloquinone may also facilitate disulfide bond formation between two Cys residues in the extracellular sensing domain of CARD/HPCA necessary for signal transduction (Laohavisit et al., 2020; Wu et al., 2020), analogous to its function as an electron carrier for oxidative protein folding in the thylakoid lumen of photoautotrophs (Furt et al., 2010).

In closing, our work highlights a link between PM phylloquinone and parasitism that warrants future investigation. RNAi silencing of *QR2* in *Phtheirospermum japonicum* has indeed demonstrated a negative effect on haustorium formation (Ishida et al., 2017). Recent advances in *Agrobacterium rhizogenes*-mediated transformation of *Phelipanche* (Libiaková et al., 2018) and CRISPR genome editing should enable experimental verification of MenA2 and MenG2 function in parasitism. The existence of the alternative pathway in nonparasitic lamiales should also motivate research into the functions of PM phylloquinone in photoautotrophic species.

## Materials and methods

### Transcriptome assembly of parasitic plants

RNA-Seq data of *Phelipanche aegyptiaca*, *Striga hermonthica* and *Triphysaria versicolor* were downloaded from the Parasitic Plant Genome Project database (http://ppgp.huck.psu.edu/). After quality control filtering, cleaned reads were assembled using the custom PLAS pipeline. Briefly, PLAS performs reference-guided read mapping using Bowtie 2 v2.2.3 (Langmead and Salzberg, 2012) against the closely related *Mimulus guttatus* proteome binned by homology to facilitate parallel computing. Mapped reads were used for *de novo* assembly by Trinity (Haas et al., 2013), and the assembled contigs were quality-checked against the reference in each bin by Blast before being used as baits in the next round of *de novo* assembly. This process was repeated for up to 10 iterations until the output was stable. Assembled sequences were filtered to remove potential contaminations (e.g. host plants, protozoa, invertebrates, bacteria, fungi, and human sequences) and redundant contigs sharing ≥95% sequence identity. The transcriptome was annotated against *A. thaliana* and *M. guttatus* proteomes and UniProt database. Transcript abundance was estimated using eXpress 1.5.1 (Roberts and Pachter, 2013). Additional *MenA* and *MenG* sequences were obtained from the 1KP database (https://db.cngb.org/onekp) by BlastN against the Core Eudicots/Asterids clade using *P. aegyptiaca* sequences as query. The assembled transcriptome sequences of parasitic plants, the PLAS pipeline and other codes are available at https://github.com/TsailabBioinformatics/PM-PhQ. All phylloquinone and relevant transcripts described in this work were manually curated (Supplemental Data Set S1). *PaMenA2* and *PaMenG2* were further confirmed by RT-PCR. Briefly, cRNA derived from RNA isolated from stages 2 and 3 as described in Yang et al. (2015) was reverse transcribed using SuperScript™ IV reverse transcriptase (Invitrogen) with mixed random hexamers and gene-specific reverse primers (Supplemental Table S2). PCR was performed using Q5 High-Fidelity DNA Polymerase (NEB) with gene-specific primers (Supplemental Table S2), column purified for Gibson assembly into pUC19 using NEBBuilder^®^ HiFi DNA Assembly Master Mix, and confirmed by Sanger sequencing (Eurofins Genomics).

### Subcellular and transmembrane domain prediction and gene structure

Phylloquinone gene sequences of photosynthetic species were downloaded from Phytozome v11. Subcellular localization was predicted by Predotar 1.04 (Small et al., 2004), TargetP 1.1 (Emanuelsson et al., 2007), Protein Prowler 1.2 (Bodén and Hawkins, 2005), and WolF PSORT (Horton et al., 2007). Transmembrane domain was predicted by TMHMM Server v.2.0 (Krogh et al., 2001), and data plotted in *R*. Gene structures were drawn by Gene Structure Display Server 2.0 (Hu et al., 2015). The exon–intron junctions of parasitic *MenA* and *MenG* were inferred by Blast alignment of transcripts against genomic short read data of *P. aegyptiaca* (NCBI Sequence Read Archive accession SRX2067908), *S. hermonthica* (SRX2067907), and *T. versicolor*. Sequence alignment was performed using MUSCLE 3.8.31 (Edgar, 2004) and visualized with Color Align Conservation (www.bioinformatics.org/sms2/color_align_cons.html).

### Coexpression analysis

Transcripts with an FPKM ≥2 in at least two samples were subject to pairwise Gini correlation coefficient (GCC) computation using a python script. Phylloquinone-coexpressed transcripts were defined as the 500 most highly correlated transcripts or those with a GCC ≥0.8 for each phylloquinone gene, excluding the alternative *NDC1* and *QR2*. The union sets were subjected to GO enrichment analysis using topGO 2.26.0 (Alexa et al., 2006). To facilitate comparative analysis between species, ortholog groups were constructed by OrthoFinder 1.0.8 (Emms and Kelly, 2015). Network visualization was performed in Cytoscape 3.4.0 (Shannon et al., 2003) using prefuse force-directed layout, with a GCC cutoff of 0.6.

### RNA-seq data processing of photoautotrophic species

RNA-seq data of *A. thaliana*, *Glycine max*, and *Populus tremula* × *alba* were downloaded from the NCBI SRA and quality control processed by Cutadapt 1.9.dev1 (Martin, 2011) and Trimmomatic 0.32 (Bolger et al., 2014). Reads were mapped by Tophat 2.0.13 (Kim et al., 2013), alignment sorted by Samtools 1.2 (Li et al., 2009), and read count and expression estimation obtained by HTseq 0.6.1p1 (Anders et al., 2015) and DESeq2 (Love et al., 2014). *Arabidopsis thaliana* data used for GCC computation (excluding stressed samples) were SRA236885, SRA091517, SRA269936, SRA219425, SRA308579, SRA050132, SRA067724, SRA291734, SRA269101, SRA098075, SRA100242, SRA122395, SRA163488, SRA064368, SRA246225, SRA248861, SRA202878, SRA201550, SRA303151, SRA221137, SRA272654, and SRA221060. *G. max* data included SRA187830, SRA047293, SRA036577, SRA116533, SRA091756, SRA187830, SRA036538, SRA036577, and SRA129337. *P. tremula* × *alba* datasets were SRA274261 and SRA097208.

### Phylogenetic tree construction

The protein sequences of *Phelipanche ramosa* PrPRX1 (AAY89058) and PrPOX1 (AAU04440), and *Striga asiatica* SaPOXA (AAB97853) and SaPOXB (AF043235) were searched against the PLAS assemblies of *P. aegyptiaca*, *S. hermonthica*, and *T. versicolor* to identify orthologs. Their protein sequences were aligned by MUSCLE 3.8.31 (Edgar, 2004) and the alignment trimmed by Gblocks (Castresana, 2000). Bayesian phylogenetic tree was constructed using MrBayes 3.2.5 (Ronquist et al., 2012). Phylogenetic trees for MenA and MenG were similarly constructed using protein sequences obtained from Phytozome or 1KP followed by manual curation (Supplemental Data Set S1), and visualized by MEGA X (Kumar et al., 2018).

### Phylloquinone analysis

*Phelipanche aegyptiaca* seeds were surface-sterilized, pre-conditioned on moist filter paper for 7–10 d, and treated with GR-24 for 4–6 d before collection of stage 1 germinated seeds as described (Westwood et al., 2012). Three biological replicates from independent germination experiments were used for the analysis. Nonimbibed *A. thaliana* (Col-0) seeds from three independent collections were used without further treatment. Tissues were snap-frozen in liquid nitrogen, freeze-dried, and milled to a fine powder for phylloquinone analysis as described (Booth et al., 1994). Tissues were partitioned twice in isopropanol:hexane (3:2 v/v), with menaquinone MK-4 (Sigma V9378) as a reference for *P. aegyptiaca* analysis. The hexane phase was dried under nitrogen, and resuspended in methylene chloride:methanol (1:4, v/v), 10 mM ZnCl_2_, 5 mM Na-acetate, and 5 mM acetic acid. Isocratic reverse-phase HPLC (Agilent Eclipse Plus C18 column, 5 µm, 4.6 × 250 mm) was carried out using methanol:methylene chloride (9:1, v/v), with post-column Zn reduction and fluorescence detection (excitation 244 nm, emission 418 nm). Phylloquinone peaks were verified by comparison with the authentic standard (Sigma 47773) and concentrations were estimated using calibration curves of the standard.

### *Nicotiana benthamiana* transformation and confocal microscopy

*PaMenA2* and *PaMenG2* coding sequences were gene-synthesized (General Biosystems) and Gibson-assembled with PCR-amplified GFP (from pCXDG) into *Bam*HI-digested pCXSN vector (Chen et al., 2009; see Supplemental Table S2 for primers). Due to concern over 35S promoter silencing in co-transformation experiments, another PaMenA2-GFP construct was prepared by PCR using pCX-PaManA2-GFP as template and primers containing a viral 2A peptide bridge sequence (Luke et al., 2015) to link the hygromycin phosphotransferase (HPT)-coding sequence to PaMenA2-GFP behind double 35S promoter as one transcriptional unit (*35S:PaMenA2-GFP-2A-HPT*; Supplemental Table S2). All constructs were sequence verified. *Agrobacterium*-mediated transformation of *Nicotiana benthamiana* was performed as described and regenerated on selection media (Pathi et al., 2013) using individual constructs (pCX-PaMenA2-GFP and pCX-PaMenG2-GFP) or in conjunction with a PM (AtPIP2A-mCherry) marker (Nelson et al., 2007; pCX-PaMenA2-GFP-2A-HPT + AtPIP2A-mCherry and pCX-PaMenG2-GFP + AtPIP2A-mCherry). Root samples from independent transformants were screened under a fluorescence microscope and at least three positive lines were further examined using a Zeiss LSM 880 upright confocal microscope in the Biomedical Microscopy Core at the University of Georgia. GFP signal was detected using an Argon excitation laser (488 nm) and an emission filter at 490–540 nm, and mCherry signal was obtained using a HeNe excitation laser (543 nm) and an emission filter at 547–697 nm.

### *Escherichia coli* complementation

The *PaMenG2* and *EcMenG* (positive control) coding sequences were PCR-amplified (see Supplemental Table S2 for primers) for Gibson assembly into a constitutive expression vector pUCM (Kim et al., 2010), and transformed into *E. coli* DH5α. Sequence-verified plasmids were transformed into *E. coli* strain JW5581 carrying the mutated *menG* (*ubiE*) gene (obtained from the *E. coli* Genetic Stock Center). At least two PCR-confirmed colonies per construct were used for complementation experiments. Approximately 30 mg of freeze-dried bacterial cells were extracted as described (Booth et al., 1994) using a Misonix sonicator. Reverse-phase HPLC conditions were the same as above except with a run time of 40 min.

### Accession numbers

The accession numbers of genes mentioned in this work are provided in Supplemental Table S3. Additional sequences from PLAS or 1KP are provided in Supplemental Data Set S1.

## Supplemental Data

The following materials are available in the online version of this article.

**Supplemental Figure S1** MenE sequence alignment with C-terminal peroxisome targeting signal PTS1.

**Supplemental Figure S2**. MenB sequence alignment with N-terminal peroxisome targeting signal PTS2.

**Supplemental Figure S3**. DHNAT sequence alignment with C-terminal peroxisome targeting signal PTS1.

**Supplemental Figure S4**. MenA sequence alignment.

**Supplemental Figure S5**. MenG sequence alignment.

**Supplemental Figure S6**. QR2 sequence alignment.

**Supplemental Figure S7**. The signal intensity profiles of plasma membrane marker AtPIP2A with PaMenA2 or PaMenG2.

**Supplemental Table S1**. Plastid-targeting prediction for ICS, PHYLLO and NDC1.

**Supplemental Table S2**. List of primers.

**Supplemental Table S3**. Accession numbers.

**Supplemental Data Set S1**. PLAS-assembled or 1KP-derived and manually curated transcript sequences of phylloquinone biosynthetic and coexpressed genes described in the manuscript.

**Supplemental Data Set S2**. Lists of transcripts used in coexpression network analysis.

## Supplementary Material

kiab031_Supplementary_Data

## Notes

C.-J.T. and X.G. conceived the study; X.G. performed all bioinformatic analyses with contributions from C.-J.T.; I.-G.C. performed bacterial complementation, plant transformation and microscopic analyses with help from M.A.O.; S.A.H., B.N., and I.-G.C. performed metabolic analyses; K.C. and J.H.W. contributed materials; X.G. and C.-J.T. wrote the manuscript with input from S.A.H. and J.H.W.

The author responsible for distribution of materials integral to the findings presented in this article in accordance with the policy described in the Instructions for Authors (https://academic.oup.com/plphys/pages/general-instructions) is: Chung-Jui Tsai (cjtsai@uga.edu).
