# Supplementary material for: Plasma membrane phylloquinone biosynthesis in nonphotosynthetic parasitic plants
Source: Plant Physiol. 2021 Jan 30;185(4):1443–56. doi: 10.1093/plphys/kiab031 (PMC8133638; doi:10.1093/plphys/kiab031)
Supplement: kiab031_Supplementary_Data [file kiab031_supplementary_data.zip › pp.01123.2020-s01.pdf]

Supplementary Materials

Xi Gu, Ing-Gin Chen, Scott A. Harding, Batbayar Nyamdari, Maria A. Ortega,
Kristen Clermont, James H. Westwood, Chung-Jui Tsai

```
AT1G30520          MANHSRPHICQCLTRLASVKRNAVVTVYGNRKRTGREFVDGVLSLAAGLIRLGLRNGDVVSIAAFNSDLFLEWLLAVALV 80
Potri.011G164100   MANYSQAHICQCLTRLSTLRSTSVVTISGNRQKTGHQFVGDVLSLAHGLLQLGLGNGDIVAICGFNSDWYLEWLLAVAFV 80
Solyc02g069920     MANYSKAHVCQCLSRLSTVRRSSTVMIIGERRKTGMEFVEGVLGLAHGLIQLGLNPGDVVAISALNSDLYLEWLLAVAYV 80
Migut.H01327.1     MANYSEAHICQCLSRLSAAARHSTVTINGDRRKNGMQFVDGVMALVRGLLQLGINPGDVVSISALNSDLYLEWMLAITYI 80
TvMenE             MANYSEPHICQCLSRLAAVSRDPTVTICGDRRKTGTRFVGGVMGLAHGLLQLGIKPGDVVSISALNSDLYLEWMLAITYV 80
ShMenE             -------------------------------------------------------------------------------- 0
PaMenE             MANYSESHICQCLSRLAAVSRISTVTIYGDRRKTGMQFVEEVMGLAHGLLQLGIKPGDVVSISALNSDLYLEWMLAITYV 80

AT1G30520          GGVVAPLNYRWSLKEAKMAMLLVEPVLLVTDETCVSWCIDVQNGDIPSLKWRVLMESTSTDFANELNQFLTTEMLKQRTL 160
Potri.011G164100   GGIVAPLNYRWSFEEAKSAMLMVRPVMLITDESCKHWYQELQSNALPSVKWHVFSGSPSSGFVKT-SNVLTTETLRKHVI 159
Solyc02g069920     GGITAPFNYRWSLEEARAALQVAKPTILVHDATGNFWKSESYADSVASLRWQVLMDTPCELHST--NIGLTTEQLKRSCE 158
Migut.H01327.1     GGIAAPLNYRWSLEEAKSAMEVANPVLLVTDSSRGYWHSKFEIDSVPSLRWHVLMDVPVESNNN--GTIFATELLKEPAG 158
TvMenE             GGIAAPLNYRWSLEEAKSAMELVRPVLLVTDSSRGYWHSKFQIDSVPFLRWHALMDMPLKSHSS--GT---TELLKEPA- 154
ShMenE             -------------------------------------------------------------------------------- 0
PaMenE             GGIAAPLNYRWSLEEAKSALEVARPVLLVTDSSPGYWHSKFQIDYVPSLRWHVLMDMPVKADST--RTIFAAEFLKEPAG 158

AT1G30520          VPSLATYAWASDDAVVICFTSGTTGRPKGVTISHLAFITQSLAKIAIAGYGEDDVYLHTSPLVHIGGLSSAMAMLMVGAC 240
Potri.011G164100   GTKQLDYSWAPEGAVIICFTSGTTGRPKGVIVSHSAMIVQSLAKVAAVGYSEDDVYLHTAPLCHIGGLSSAITMLMVGGC 239
Solyc02g069920     RRLTADYLWAPKEAAIICFTSGTTGRPKGVTLSHSALVVQSLAKIAIVGYGEDDVYLHTAPLCHIGGISSALAMLMAGGR 238
Migut.H01327.1     RFIELDYLWAPERAAVICFTSGTTGKPKGATISHSALIMQSLAKIAIVRYSEDDVYLHTAPLCHIGGISSAMAMLMAGGC 238
TvMenE             ---VLDYLWAPERAAIICFTSGTTGRPKGATISHSALIVQSLAKLAIVRYDEDDVYLHTAPLCHIGGISSALAMLMAGGC 231
ShMenE             ----------------------------------------------------------------------------MAGGS 5
PaMenE             RSVKVDYLWAPERAAIICFTSGTTGRPKGATISHSALIVQSLAKIAIVRYNEDDVYLHTAPLCHIGGISSALAMLMAGGC 238

AT1G30520          HVLLPKFDAKTALQVMEQNHITCFITVPAMMADLIRVNRTTKNGAENRGVRKILNGGGSLSSELLKEAVNIFPCARILSA 320
Potri.011G164100   HVLIPKFEASLAIEAIKQHCVTSLITVPAMMADLISLTRLKETWKGRQYVKKLLNGGGSLSAELMKDATELFPRAKLLSA 319
Solyc02g069920     HILLPKFEAKLAVESIDQHSVTSLITVPAMMTDLISFYKTKHISVGSKSVKKVLNGAGGLSSSLIKNVIEIFPRAKLLSA 318
Migut.H01327.1     HVIIPKFEANLAFEAIREYNVTSLITVPTMMADLISYNRTKQKSESFESVKKILNGGGGLSVELINNATKLFPRATIQSA 318
TvMenE             HVILPKFEANLAIKAIREHNVTSLITVPTMMADLISINRMNQTFETFESVKKVLNGGGGLSVDLIKNATEIFPRATLISA 311
ShMenE             HVILPKFEANLAIEVIKKHNVTPLITVPALMADLISFNRLNRTSVTYESVKKILNGGGGLSTELTKDTIEIFLSATLMSA 85
PaMenE             HVILPKFEASLAIEAIREHSVTSLITVPTMMADLISSHRMDQTSTSFESVKKILNGGGGLSVELIKDATKLFPLATLLSA 318

AT1G30520          YGMTEACSSLTFMTLHDPTQES---------FKVTYPLLNQPKQGTCVGKPAPHIELMVKLDEDSSRVGKILTRGPHTML 391
Potri.011G164100   YGMTETCSSLTFMTLHDPTLQTPAQTLQT--VDKTKSSSAHQPHGVCVGKPPPHVELKISADEPST-IGRILTRGPHLML 396
Solyc02g069920     YGMTEACSSLTFMTLYDPARESCIQH-----SYANSSNLAHKPDGICVGKPAPHIEIRIAGDDSSC-IGRILTRGPHLML 392
Migut.H01327.1     YGMTEACSSLTFMTIYEPTKESRFVHV----NDVQKSNL-NCQGGVCVGKPAPHVELKISCDGSSNNIGRILMRGPHAML 393
TvMenE             YGMTEACSSLTFMTLYDPTKENPHKQSSSYYNDVRKSNL-SREGGTCVGKPAPHVELKVSGDGSPNNTGRILMRGPHVML 390
ShMenE             YGMTEACSSLTFMTLFDPTKTT---------HQKYHSSF---QGGICVGQSATHVKLKISPDEKSLNTGKILTRGPHVML 153
PaMenE             YGMTEACSSLTFMTLYEPTKEGHHLQP----HDIQKSNLISCQGGICVGKPAPHVELQVNAEE-SCNTGRILMRGPHVML 393

AT1G30520          RYWGHQVAQENVETSESRSNEAWLDTGDIGAFDEFGNLWLIGRSNGRIKTGGENVYPEEVEAVLVEHPGIVSAVVIGVID 471
Potri.011G164100   RYWDQ--NPMK---ATESTNDFWLDTGDIGSIDDCGNVWLVGRQNAQIKSGGENIYPEEVEAMLLQHPGVIATVVVGVPE 471
Solyc02g069920     GYWDQ--MPSN---NSSPVDECWLDTGDIGHIDDCGNIWLVGRLKGRIKSGGENVYPEEVEAVLLQHPGISASVVIGLPD 467
Migut.H01327.1     RYWGQ-----------NGPRQSWLDTGDIGQIDDHGSLWLIGREKGRIKSGGENIFPEEVEGVLLQHPGISSIAVVGVPD 462
TvMenE             HYWGQ--SPSD---HLDPVYGSWLDTGDIGQLDGHGNLWLVGRAKDRIKSGGENIYPEEVEAVLAQHSGISKIVVVGIPD 465
ShMenE             GYWGQRTSKSN---HSKPGLEGWLDTGDIGQIDDHGNLWLAGRAKDRIKSGGENVHPEEVECVLSQHPGISRIVVIGIPD 230
PaMenE             RYWGQ--SPSK---HLSPVYEGWLDTGDIGQVDNHGNLWLIGRAKDRIKSGGENIYPEEVEAVLSQHLGISRIVVVGIPD 468

AT1G30520          TRLGEMVVACVRLQEKWIWSDVENR-----KGSFQLSSETLKHHCRTQNLTGFKIPKRFVRWEKQFPLTTTGKVKRDEVR 546
Potri.011G164100   ARLTEMVVACIKLRQSWQWTNNNCKQSA--ENNLTLCREVLRDYCREKKLTGFKVPKLFILWRKPFPLTTTGKIRRDQVR 549
Solyc02g069920     SRLTEMVVACIRMKDNWQWTDSSSNHPVN-KNEHCLSSTVLQNFCRAKDLTGFKIPKKFVVWKNQFPMTTTGKLRRDQVR 546
Migut.H01327.1     SRLTEMVIACVRLNDSWRWDDFGAINHSE-EYTKCVSSEILKRFCRQKNLTGFKIPKRFVLWKNAFPMTTTGKLRRDKVR 541
TvMenE             SRLTEMLIACVRLKDGWRWAEFGANRSAG-DQIQCLSSEMLRHFCREKNLTGFKIPRRFALWTNDFPMTTTGKLKRDRVR 544
ShMenE             ARLTEIVTACVQLKSDWQWVDFGAGRSAGVEQVKCLSSEILRQFCRQKNLTRFKIPKRFILWENDFPTTTTGKLRRDQLM 310
PaMenE             SRLTEMVFACIKLNDGWRWTDFVGNHSAG-EHIQCLSSEILKRFCREKNLTGFKIPKRFVLWKNDFPITTTGKLRRDQVR 547

AT1G30520          RQ-VLSHFQIMTSSL 560
Potri.011G164100   RE-VMSHLQFFPSNL 563
Solyc02g069920     AE-LMSFRQLAPSRL 560
Migut.H01327.1     EDVLLSHTQFLSSKL 556
TvMenE             EEVVMSHTRFLPSKL 559
ShMenE             VE-VMSHTQFVSSKL 324
PaMenE             AE-VMSHTQFLTSKL 561
```

Supplemental Figure S1. MenE sequence alignment with C-terminal peroxisome targeting signal PTS1

MenE sequences from the three parasitic species and representative non-parasitic plants from Phytozome are shown. The C-terminal PTS1 is boxed in red. The assembled *ShMenE* transcript sequence contains stop codons in the 5'-end, leading to truncated polypeptide sequence.

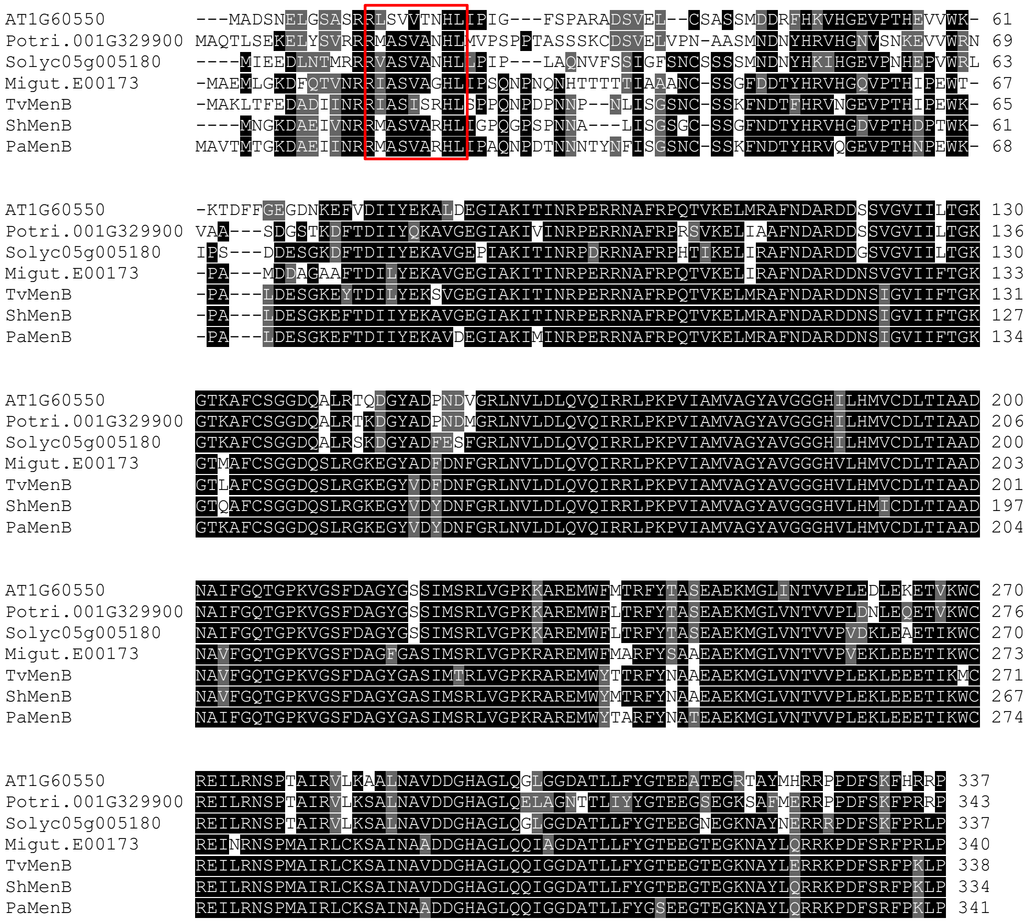

Supplemental Figure S2. MenB sequence alignment with N-terminal peroxisome targeting signal PTS2

MenB sequences from the three parasitic species and representative non-parasitic plants from Phytozome are shown. The N-terminal PTS2 is boxed in red.

```
AT5G48950          M------------DPKSPEFIIDQPLKILGFVFDELSATRVSGHLTLTEKCCQPFKVLHGGVSALIAEAL 58
AT1G48320          MD-----------SASSNTKAIDPPLHMLGFEFDELSPTRITGRLPVSPVCCQPFKVLHGGVSALIAESL 59
Potri.010G003600   -----------------------------------MSPQRVTGRLLVNPMSVQPFKVLHGGISALISESM 35
Potri.010G003800   MEQSSSSSSS---SSSSKKESLDVPLHSFGFQIEELSPQKVTGRLLVTPKCVQPFKVLHGGVSALISEAM 67
Solyc03g006440     MEQLT--------KSIPMKEILDAPLDVIGFKISEISPQKIFGYFLVVKKCCNPFNVLHGGVSALVAESL 62
Solyc02g078410     MGSPSAAA-----AAGVKARVLDIPLHGIGFEFVEITPHKITGRLPVTDKCCQPFKVLHGGVSALMAESL 65
Solyc03g006450     MEPSA--------KSIPIKDLLDAPLDVIDFKITEVSPHKIFGYFSVTEKCCNPFEVLHGGVSALTAESL 62
Migut.K00082       MNQIGDGGEP---PSSSKTEILDSPLHMFGFEIDELSPHRVSGHLLVTPKCCQPFKMLHGGVSALIAEAL 67
TvDHNAT3           MNQPPPNAGP---PPQSMTEKLDVPLHWLGFEIDCLSPDKVSGHFIITSKSSQAFKVLHGGVSALIAESL 67
TvDHNAT2           MTQPPSAVGPPPLPPPAKTEELDSPLHLIGFEIDCLSPDKVSGHIIITSKCCQPFKVLHGGVSALIAESL 70
TvDHNAT1           MSESLP-------PAVKRMELLDAPLHLFGFELDELSPHKVSGHLLITSKCCQPFNMLHGGVSALIAEAL 63
ShDHNAT1           MNR----------PLPLNTKDLDIPLHTIGFEIDCISPEKVSGHVLITEKCCQPFKVLHGGVSALIAEAL 60
ShDHNAT2           MNRTPSATGPPP-PPPSKTKELDIPLHTIGFEIDCLSPQKVSGHVLISEKCCQPFKVLHGGVSALIAESL 69
PaDHNAT            MNQPPPSARPP--SPPSNTKELDFPLHTLGFKFDCLSPDKVSGHLLITSECCQPFKVLHGGVSALIAESL 68

AT5G48950          ASLGAGIASGFKRVAGIHLSIHHLRPAALGEIVFAESFPVSVGKNIQVWEVRLWKA-KKTETPDNKIMVS 127
AT1G48320          ASMGAHMASGFKRVAGIQLSINHLKSADLGDLVFAEATPVSTGKTIQVWEVKLWKT-TQKDK-ANKILIS 127
Potri.010G003600   ASLGAHLASGLQRVAGIQLSINHVKSAHVGDLIVAEATPSSIGKTIQVWDVRIWKLSDPSNTESSKSLVS 105
Potri.010G003800   ASMGAHMASGLQRVAGIHLSINHVKSARLGDLVLAEATPFSIGKTIQVWEVRIWKLADPSNTESSKSLVS 137
Solyc03g006440     ASMGAHVASGFERVAGVHLSIHHLRSANLGEIVYAEAKPLNVGKSIHVWEVNLWKN-DSLIL-GERILIS 130
Solyc02g078410     ASMGAHMASGFKRVAGVHLSIHHLKSAHLGDLVFAEAVPINIGKSIHVWEVCLWKI-DPANE-EKKTLLA 133
Solyc03g006450     ASIGAYVASGFDRVAGVHLSIHHLRSANIGEIVYAEAKPLNVGKSIHVWEVNLWKN-NSLIL-GERILIS 130
Migut.K00082       ASMGAHIASGFHRIAGIHLSISHLKSAQAGDFVVAVATPVNLGKSIQVWEVSLSKC-DPSNS-EIKTLIA 135
TvDHNAT3           ASIGAHLASGQQRVAGVHLSISHLKSAQLGDFIVAEATPVNIGKTIQVWEVRLSKCDDPLNT-ETKTLIS 136
TvDHNAT2           ASIGAHLASGQQRVAGVHLSINHLKSAKLGDFVVAEATPVNIGKTIQVWEVRLSKCDDPSNT-EIKTLIS 139
TvDHNAT1           ASIGAHMASGFHRVAGIQLSINHLKPAQAGDFVIAEATPVSVGKSVQVWEVRLFKC-DPLKSDEIRTLIA 132
ShDHNAT1           ASTGAYLASGLQRVAGVHLSINHLKSAKLGDYVVAEATPVNIGKSIQVWEVNLSKC-DPSNS-ETKTLIS 128
ShDHNAT2           ASIGAHLASGLQRVAGVHLSISHLKSAKLGDFVVAEAKPVNIGKSIQVWEVNLSKS-DSSNS-ETKTLIS 137
PaDHNAT            ASIGAHLASGLQRVAGVHLSISHLKSAKLGDTVLAEATPVNIGKTIQVWEVNLSKC-DPSDS-EIKTLIS 136

AT5G48950          TSRVTLFCGLPIPDHVKDAPDELKKVISKL 157
AT1G48320          SSRVTLICNLPIPDNAKDAANMLKMV-AKL 156
Potri.010G003600   SSRVTLICNLPVPDHAKEAVENLRSQ-AKL 134
Potri.010G003800   SSRVTLMCNLPVPDHAKEATENLRSH-AKL 166
Solyc03g006440     TSRVTIKTNMPLPKNVKDADVIIKKY-AKL 159
Solyc02g078410     SSRVTLKVNMSIPENAKDAAVNLKKY-AKL 162
Solyc03g006450     SSRVTIKTNMPLPKNVKEAAMNIKKH-AKL 159
Migut.K00082       SSRVTILCNLPVPESARDAAQNLKKY-AKL 164
TvDHNAT3           SSRVTLLCNLPVPESFKTTAQGLKKY-AKL 165
TvDHNAT2           SSRVTLICNLPVPESLKSAAQGLRKY-AKL 168
TvDHNAT1           SSRVTVFCNLPVPESSRDAAQNLKKY-SKL 161
ShDHNAT1           SSRVILLCNLPVPDSLKEAAQGFKKY-SKL 157
ShDHNAT2           SSRVTLLCNLPVPESLKTAAQGLKKY-AKL 166
PaDHNAT            SSRVTLLCNLPVPESLKAAAQGLKKY-ARL 165
```

Supplemental Figure S3. DHNAT sequence alignment with C-terminal peroxisome targeting signal PTS1

DHNAT sequences from the three parasitic species and representative non-parasitic plants from Phytozome are shown. The C-terminal PTS1 is boxed in red.

```
At1g60600         ----MVNFVSLC--DIKYGFVPKNSTDLFVKRKI-----HKLPSRGDVITRLPVFGSNARENLNAKPRRNLRVRPIFCKSY--------- 70
Potri.017G050900  ------MAAAFC--SLHQGSSLKKLQYYHVRQSYATRSYPGALTSNASKKSSLCCCTAQNFVCGSRISRLEFKKGIQCWRAFSGSIANYA 82
Solyc01g105460    ----MASSATFNVPILVSAFNKTSHAPISSRNFILRTC----SLPSHVRQRSICCFQCTGRSGSVIDLGWKEVKQWHSSLRGFFMCKVQQ 82
Migut.B00584      MAMAAAAAATFC--SISHGYGVTKLNSF---RICIYSRYQSRAITHENVFRL---DARK----------LHSIQRRYIIPLKPRASSN-- 70
VtMenA1           -----MAAANFC--SMSHGYGVKNLNQFLPRRCNNYRAYQELSPQDAVRMPL---CSKTYFDKTIIRR-LHSKKPHHRILFKCAAELI-- 77
LpMenA1           -----MAAATFC--SISHGYGVSRVNDYLLNRHGINRIDQVLPLPDASRRRK---LVKKDFNKTVIRQ-LHSIRRHYTMSLKFRAENN-- 77
RgMenA1           -----MAAATFC--SISHGYGVNKLNQHLLNRHNFNRIYQVIPLPDASRRQL---RMEMCFDKTIIRQ-LHCIRRHYGILLKCRSEHN-- 77
TvMenA1           -----MAAATFCSISISHGYAVQRLN-----RHKINRTYQVLPLVCGSRTTK------VHLNKTIIRQYLHSIQRRYKISPKFRSENNAD 74
ShMenA1           MAPLAVAAAVYC--STSHGYGVKKLDDYLTRKLSISRIHQVLLLPDACQRSL---CTKFNFKKASMRQ-LYSLRGHYINSFKQRAEHCG- 83
Migut.B01155      ----MLS-------SIQENYAVTG------------------NLHADDVTPE-------------------------------------- 23
Migut.B01157      ----MLS-------SIQENYAVTG------------------NLHADDVTPE-------------------------------------- 23
RgMenA2           -----MAGTSF---SSSDN-----------------------PSKEKNGKRS---KKE-------------------------------- 24
LpMenA2           -----MAGTSL---NLVEN-----------------------ASKEKNIKRS---KNGS------------------------------- 25
PaMenA2           ----MAEAANH-------------------------------PNQANIVKRL-------------------------------------- 17
CaMenA2           ----MAGGANFI--SLADN-----------------------PSLEKNVKRS-------------------------------------- 23
AfMenA2           ----MAGGSSI---SLADN-----------------------PTQEKVVRRS-------------------------------------- 22

At1g60600         GDAAKVYQE-EE----IPRAKLIWRAIKLPMYSVALVPLTVGASAAYLETGLFLARRYVTLLLSSILIITWLNLSNDVYDFDTGADKNKM 155
Potri.017G050900  DKSHEGETG-ED----VPKATLIWRAVKLPIYSVALVPLTVGGAAAYLQTGMFSARRYFSLLVSSILIITWLNLSNDVYDFDTGADKNKK 167
Solyc01g105460    HDATSEENE-EN----ISQATLIWRAIKLPIYSVALIPLTVGSAAAYLQTGLFSARRYFMLLASSVFIITWLNLSNDVYDFDTGADKDKK 167
Migut.B00584      EDHIQHEKEVNN----ISKATLIWRAVKLPMYTVAFIPISVGSAAAYLQTGKYFAKRYFVLLVSSVLVIAWLNLSNDVYDFDTGADKNKK 156
VtMenA1           DTHGEDEKE-EH----ISKATLIWRAIKLPIYTVALIPITVGNAAAYLQTGQYYGKRYVMLLVSSFLIIAWLNLSNDVYDFDTGADKNKK 162
LpMenA1           GDRIEEEKE-GN----VSKATLIWRAIKLPIYTVALIPITVGSAAAYLQTGQYFGKRYVMLLISSVFVIAWLNLSNDVYDSETGADKNKK 162
RgMenA1           DSHVVEEKE-ES----ISKATLIWRAIKLPMYTVALIPITVGSAAAYLQTGQYFGKRYLMLLISSVLIIAWLNLSNDVYDFETGADKNKK 162
TvMenA1           NTHVEEEEDEDEQESVSKATLIWRAIKLPMYTVALIPITVGSAAAYLQTGQYFGKRYIMLLVSSVLIIAWLNLSNDVYDFDAGADKNKK 164
ShMenA1           SSNIEENKE-ES----ISRVALMWRAIKLPIYSVALIPITVGSAAAYLQTGQFFGKRYLKLLLSSVLIITWLNLSNDVYDFETGADKNKK 168
Migut.B01155      --DETKTRD-DD----VSRATLIWRAAKLPMYTVALIPVTVGTAAAYWQTGLYSSARYFKILASYVLVIVWLNLSNDVYDFDTGVDVNKK 106
Migut.B01157      --DETKTRD-DD----VSRATLIWRAAKLPMYTVALIPVTVGTAAAYWQTGLYSSARYFKILASYVLVIVWLNLSNDVYDFDTGVDVNKK 106
RgMenA2           TTSHDEKKE-ED----IGRATLIWRAAKLPMYTVALIPLTVGTAAAYWESGYYSLERYFILLASFVLVNLWVNLSNDVYDFDTGADINKK 109
LpMenA2           TSPNEEKNE-EE----ISRATLIWRAAKLPMYTVALIPLTVGTSAAYWESGYYSLERYFILLASFVLVNVWVNLSNDVYDFDTGADKNKK 110
PaMenA2           --HKKEKKE-GD----ISRATLIWRAAKLPMYTVALTPLTVGTAAAYWESGFYSLERYFTLLASFVLVNLWINLSNDVYDFDTGADKNKK 100
CaMenA2           KKKGEKKEE-ED----ISRATLIWRAAKLPMYTVALIPLTVGTATAYWESGYYSLERYFILLASFVLVNVWINLSNDVYDFDTGADKNKR 108
AfMenA2           ----KSKKE-ED----ISRAILIWRAAKLPMYTVALIPLTVGTAAAYLESGFYSLERYFILLASFVLVNVWVNLSNDVYDFDTGADKNKK 103

At1g60600         ESVVNLVGSRTGTLAAAITSLALGVSGLVWTSLNASNIRAILLLASAILCGYVYQCPPFRLSYQGLGEPLCFAAFGPFATTAFYLLLGSS 245
Potri.017G050900  ESVVNLVGSRSVAFIAAYSSLLLGFAGLAWTSMGEGNIHAILFLACAILCGYVYQCPPFRLSYQGLGEPLCFAAFGPFATSAFYLLLGST 257
Solyc01g105460    ESVVNIVGSRTGTFVAACLLLALGFLGLTYTSVEAANLRAILLLASAITCGYIYQCPPFRLSYQGLGEPLCFAAFGPFATTAFYLLQSNT 257
Migut.B00584      ESVVNLLGSRTGAHVIAWLLLGIGFTGLTLVSIEAKSIRSILLLACAVVCGYIYQCPPFRLSYMGLGEPLCFAAFGPLATTAFYLLQSGR 246
VtMenA1           ESVVNLIGSRKGTRAFAWSLLALGFMGLTRVSMDAGSFYPLFLLACAIICGYIYQCPPFRLSYMGLGEPLCFAAFGPFATTAFYFLQTGT 252
LpMenA1           ESVVNLFGSRTGTHVFAWLLLALGFTGLTLVSVEARSRHSIFLLACSVFCGYVYQCPPFRLSYLGLGEPLCFAAFGPLATIAFYLLQGGT 252
RgMenA1           ESVVNLFGSRTGTHVFAWLLLALGFTGLTWVSVEAGSLRSIFLLGCAVFCGYIYQCPPFRLSYLGLGEPLCFAAFGPFATTAFYLLQSGT 252
TvMenA1           ESVVNLFGSRTGTHVFAWLLLALGFAGLARVSVEAGSLRSIFLLGCAVFCGYIYQCPPFRLSYMGLGEPLCFVAFGPFATTAFYLLQSGT 254
ShMenA1           ESVVNLIGSQTGTHILAWVLLELGFGGLTWVSIEAGSIRSIILLACAIFCGYIYQCPPFRLSYLGLGEPLCFAAFGPFATTAFYLLQSGT 258
Migut.B01155      ESVINLFGSRTGINIIAWLILVLGFLGLAWVAVEARNPRSMILLASAVFCFFLYQCPPFRLSYYGVGEPLLFVAFGPLSTIAFYLLHSIK 196
Migut.B01157      ESVINLFGSRTGINIIAWLILVLGFLGLAWVAVEARNPRSMILLASAVFCSFLYQCPPFRLSYYGVGEPLLFVAFGPLSTIAFYLLHSIK 196
RgMenA2           ESVVNIVGSRNAIHILSWLLLAIGFAGLSWVGVEAKNPRAILLLAAAVFCGYIYQCPPFRLSYHGLGEPLCFASFRPFSTVAFYLLQSRS 199
LpMenA2           ESVVNIVGSRTAIHILSWLILALGFAGLTWVGVEAKNPRAILLLASAVFCGYIYQCPPFRLSYHGLGDPLCFAAFGPFSTVAFYLLQSSS 200
PaMenA2           ESVVNIFGSRTAIHILSLLVLAIGFAGLCWVALEAKNPRAILLLASTVFCVYAYQFPPYRLSYHGLGEPLCFAAYGPFATVAFYLLQSGS 190
CaMenA2           ESVVNMVGSRTAIYILSWLLLALGFAGLSWVGIEAKNPRAILLLASSIFCVYIYQCPPYRLSYHGLGEPICFAAYGPFSTIAFYLLQSTS 198
AfMenA2           ESVVNIVGSRTAIHILSWLLLALGFAGLCWVGLEANNPRAILLLASAIFCGYIYQCPPFRLSYHGLGEPLCFASFGPFSTIAFYLLQSSS 193

At1g60600         S-EMRHLPLSGRVLSSSVLVGFTTSLILFCSHFHQVDGDLAVGKYSPLVRLGTEKGAFVVRWTIRLLYSMLLVLGLTRILPLPCTLMCFL 334
Potri.017G050900  S-EMSILPLTGTILSASLLVGFTTTLILFCSHFHQVEEDKAVGKFSPLVRLGTERGSGVVKVAVAMLYSLLFASGLSRTLPLACILLCSL 346
Solyc01g105460    S-E---LPITYTTLSASALVGFTTSLILFCSHFHQVEGDKAVRKFSPLVRLGTEAGSRVVKVGVATLYSGMLLLGFTQTLPLTCIILCAL 343
Migut.B00584      S-E---LPVSGTIVSSSILVGLTTSLILLCSHFHQIEDDKAVGKISPLVRLGTERGAKVVNVAVGLLYSLVFLLGLNQTLPFFSLVLCAL 332
VtMenA1           R-D---LSISATVISSSILVGITTSLILFCSHFHQIDDDKTVGKLSPLVRLGTETGAKVVKIAVITLYSLLFVLGLSQTLPFSSIVLCAL 338
LpMenA1           R-E--QLSISCAVVASSILVGFTTSLILFCSHFHQIEDDKAVGKFSPLVKLGTEGGAKVVKVAIGTIYSLLFILGLSQTLPFSSIVLCAL 339
RgMenA1           R-E---LSISVTIVSSSILVGLTTSLILFCSHFHQIDDDKAVGKFSPLVRLGSDGGAKVVKVAVGTLYSLLFILGLSQTLPFSSIVLCAL 338
TvMenA1           R-E---LSISGIVIVSSVLVGITTSLILFCSHFHQIEDDKAVGKYSPLVRLGSEGGANVVKMVVRTIYSLLFILGLVQTLPFASIVLCAL 340
ShMenA1           S-E---LSISATVISSSILVGFTTSLILFCSHFHQIEDDKAVGKFSPVVRLGTEGGANVVKVAVRILYSLLFVLGLAQILPFPSIVLCAL 344
Migut.B01155      S-E---LPITSTIVWSSILVGLTTSLILFCSHFHQIKDDKAVGKMSPLVRLGTETGSRVVKFAVVGLYSIILVLGFTQNLPLASVILCAM 282
Migut.B01157      S-E---LPITSTIVWSSILVGLTTSLILFCSHFHQIKDDKAVGKMSPLVRLGTETGSRVVKFAVVGLYSIILVLGFTQNLPLASVILCAM 282
RgMenA2           S-E---LPISSTIISAAILVGFTSALILFCSHFHQIEDDKAVGKISPLVRLGTETGSKVVKMAVLGLYWLVFGLGLAQTLPYPCIMLCIM 285
LpMenA2           S-E---LPISSTIVSSAILVGFTSALILFCSHFHQIEDDKAVGKISPLVRLGTEKGSKVVKMAILGLYWLVLGLGLAQTLPYACIVLCAM 286
PaMenA2           SSE---LPISSTVVFASIFVGFTSALILFCSHFHQIEDDKAVGKISPLVRLGTEKASKVVKMSVLGFYWLVFGLGLSQTLPYACVVLCTM 277
CaMenA2           KFE---LPISSTIVSAAILVGFTTALILFCSHFHQIEDDKAVGKISPLVRLGTEKASKVVKMAVLGLYWLVFGFGLTQTLPYACLVLCTM 285
AfMenA2           S-E---LPLSSTIVSAAILVGSTSALILFCSHFHQIEDDKAVGKISPLVRLGTEKASEIVKMAVLGLYWLVFGLGLSQTLPYACIVLCTM 279
```

```
At1g60600          TLPVGNLVSSYVEKHHK--DNGKIFMAKYYCVRLHA---LLGAALSLGLVIAR------------------------ 382
Potri.017G050900   TLPMGKLVVGFVEENYK--DKGKIFMAKYFCVRLHA---LFGAALASGLVAAR--VFQG-YFP-------------- 401
Solyc01g105460     TIPVGKLVVSYVEKNHKVRQSKDLYGKVLLCETSCSIWSGSGCWLGSC----------------------------- 391
Migut.B00584       TLPVGRLVSSFVEKNHK--DETKIFMAKYYCVRLHT---VFGGALAAGMVAAR--MFARRQLPHTVLL--------- 393
VtMenA1            TLPIGNKIVRFVEENHK--DKTKIFMAKYYCVRLHT---VFGAALAAGLVGAK--MFARKKIPHAMIIS-------- 400
LpMenA1            TLPMGKFVVSFIEENHK--DKTKIFMAKYYCVRLHT---VFGAALAAGLVAAR--MLARKPLPYAVIL--------- 400
RgMenA1            TLPVGKLVVSFVEKNHK--DKTKIFMAKYYCVRLHT---VFGAALAAGMVAAR--MFARKQLPHAIIL--------- 399
TvMenA1            TLPVGNLVVSFVEKNHK--DKTKIFMAKYYCVRLHT---VFGAALATGLVAAR--VLARKPIPNAIIL--------- 401
ShMenA1            TLPVGNLVVSFVGTNHK--DKMKIFMAKYYCVRLHT---IFGAALAAGMVAAR--MFARKQLPHAIIL--------- 405
Migut.B01155       TLPMGNLVVSFVEKNHK--DKTKIFMGKYLCVRLHT---VFGAALAAGLVVAR--KFAVQQLPHAIVP--------- 343
Migut.B01157       TLPMGNLVVSFVEKNHM----VNIHGEVFVCEVAHCFWGCFGCWACSG----------------------------- 326
RgMenA2            TLPMGNLVVSFVQENHK--DKSKIFMAKYYCVRLHT---VFGAALAAGLVASRL-IQSGEQLQSLSTYFDRAKFAYF 356
LpMenA2            TLPMGNLVVSFVQENHK--DKSKIFMAKYYCVRLHT---VFGAALAVGLAASR--INGGDYLQSPSTYFDRAKFAYF 356
PaMenA2            TLPMGNLVVSFVQENHK--DKSKIFMAKYYCVRLHT---VFGAALAAGLVASRLIMHSGEQLQQSI--FDYAKFLYL 347
CaMenA2            TLPIGNLIVSFVQENHK--DKSKIFMAKYYCVRLHT---VFGSALAAGLVASR--MHSGELTSEFINVLRATKFAYF 355
AfMenA2            TIPMGNLVVSFVQKNHK--DKSKIFMAKYYCVRLHT---VFGAALAVGLVASRL-MHSGEQFQSLLTYFDRAEIA-- 348
```

Supplemental Figure S4. MenA sequence alignment.
MenA sequences from the three parasitic plants were aligned with representative MenAs of non-parasitic plants from Phytozome and additional sequences identified from 1KP (Supplemental Dataset S1). Af, *Aphyllon fasciculata*; Ca, *Conopholis americana*; Rg, *Rehmannia glutinosa*; Lp, *Lindenbergia philippensis*; and Vt, *Verbascum thapsus*.

```
At1g23360        -MAALLGIVSP---VTFTGKHPVNSRSRRRTVVKCSNERRILFNRIAPVYDNLNDLLSLGQHRIWKNMAVSWSGAKKGDYVLDLCCGSGD  86
Potri.008G188600 --MASLQLQLSFGYQSSSCRHLPISTFSYPS-IRCANDRQLLFNRIAPVYDNLNDLLSLGQHRIWKRMAVSWTGAKLGDCVLDLCCGSGD  87
Solyc12g019010   --MASLHVPLS-------SLPRPSFRPTGKLLIRCSADRQALFNRIAPVYDNLNDLLSLGQHRIWKRMAVSWSGAKEGDTVLDICCGSGD  81
Migut.E00183     --MATLQLNLH---SVTGGQRRPACRSVITP-IRCAAERQALFNRIAPVYDNLNDLLSLGRHRVWKRMAVSWSGAKEGDTVLDVCCGSGD  84
VtMenG1          --MATLQFIPP---SITGRSSS---RSIRRP-VQCSAERQALFNRIAPIYDNLNDLLSLGRHRVWKRMNVSWSGAKEGDTVLDVCCGSGD  81
RgMenG1          --MATLQFTLP---SITGGRRLPESRSTIRP-VRCAAERQALFNRIAPVYDNLNDLFSLGSHRIWKRMAVSWSGAKEGDTVLDVCCGSGD  84
LpMenG1          --MATLQFTLP---SITGGRRQPESRSILRP-VRCAAERQALFNRIAPVYDNLNDLLSLGAHRIWKRMAVSWSGAKEGDTVLDVCCGSGD  84
ShMenG1          --MATLHFTLP---STTGRQRPPEFRSIFKP-ARCAAERQALFDRIAPVYDNLNDLLSLGGHRVWKRMAVSWTGAKEGDTVLDVCCGSGD  84
TvMenG1          --MISLQFTLP---SITSRRSLPESRPILKP-IRCASDRQALFNRIAPVYDNLNDLLSLGAHRVWKRMAVSWSGAKEGDTVLDVCCGSGD  84
VtMenG2          --MASIR-----------RRSE--SQPAGKF-AEGAAERQELFNRIAPVYDKLNDVFSLGMHRLWKNWSISWSGAKEGNRVLDVCCGSGD  74
RgMenG2          --MATLR-----------RRSE----PQTVT-HESATERQELFNRIAPVYDKLNDLFSLGLHRLWKRWTISWSEAKEGDKVLDACCGSGD  72
LpMenG2          --MATLR-----------RRSE----PPAVT-HDSADERQELFNRIAPVYDKLNDLFSLGLHRLWKRRVISWSGAKEGDNVLDVCCGSGD  72
TvMenG2          MSTTLLR-----------RRSE----SEPAS-HEGAAERQELFNRIAPVYDKLNDVFSLGLHRLWKRWSISWSGGKEGDNVLDVCCGSGD  74
ShMenG2          --MATLR-----------RRSE----PQPPS-HESAAERQELFNRIAPVYDKLNDLFSLGLHRLWKKWSIAWSGAKEGDKVLDVCCGSGD  72
PaMenG2          MATATLR-----------RRTG----TQTAT-HQGAAERQELFNRIAPVYDKLNDLFSLGLHRLWKRWSISWTGAKEGDKVLDVCCGSGD  74
CaMenG2          -MAATLR-----------RRSEAPAQPAAAS-HEGAAQRQELFNRIAPVYDKLNDLFSLGLHRLWKRWSISWSGAKEGDKVLDVCCGSGD  77
AfMenG2          --MATLR-----------RRSE----AQPSS-HEAAAERQELFNSIAPVYDKLNDLFSLGLHRLWKRWSISWSGAKEGDKVLDVCCGSGD  72

At1g23360        LAFLLSEKVGSTGKVMGLDFSSEQLAVAATRQSLKARS--CYKCIEWIEGDAIDLPFDDCEFDAVTMGYGLRNVVDRLRAMKEMYRVLKP 174
Potri.008G188600 LAFLLSEKVGSNGKVSGLDFSKEQLLMASSRQHLLSKA--CYKNIEWIEGDATELPFPDCYFDAITMGYGLRNVVDKRKAVQEMFRVLKP 175
Solyc12g019010   LTFLLSEKVGPHGKAVGLDFSNEQLLIASTRQKLRSKT--CYKNIKWMEGNALDLPFPDSSFDAVTIGYGLRNVVDRHRAMTEICRVMKP 169
Migut.E00183     LAFLLSEKVGINGKVIALDFSKEQLKIAASRQSERSKS--CYKNIEWIEGDAMDLPFSGSSFDAATIGYGLRNVVHRKKALEEMCRVLKP 172
VtMenG1          LAFLLSEKVGTNGKVIALDFSSEQLQIAASRQRERTKA--CYKNIEWIEGDAVDLPFSDSSFDAATIGYGLRNVVDRKKALEQMFRVLRP 169
RgMenG1          LAFLLSEKVGINGKVIALDFSKEQLTIAASRQRKRSRA--FYKNIEWIEGDAVDLPFSDSSFDAATIGYGLRNVVDRKKALEEMCQVLKP 172
LpMenG1          LAFLLCEKVGIDGKVIALDFSKEQLQIAASRQLARSKA--CYKNIEWIEGDAVDLPFFDSSFDAATIGYGLRNVVDRKKALEEMCRVLKP 172
ShMenG1          LAFLLSEKVGVKGKVFAVDFSKELLQVAASRQLKRSKA--CYKNIEWIGGDAVDLPFSGSYFDAATIGYGLRNVVNRKKALEEMSRVLKP 172
TvMenG1          LAFLLSEKVGTNGKVFALDFSKEQLYIAGSRQSKRSKN--CYKNIEWIEGDAVDLPFSASFFDAATIGYGLRNVLDRKKALNEMCRVLKP 172
VtMenG2          LSFRFSEKVGINGKVIGLDYSKELLQIAATRQHKLVQSKPSYKNIEWIEGDATKLPFPNSSFDAATIGYGLRNIVDRKNALEEMVRVLKP 164
RgMenG2          LSFRLSEKVGIHGKVIALDFSKELLEIAASRQREHERSKPCYKNIEWIEGDAVALPFSDSTFDAATIGYGLRNIVDRKKALEEMHRVLKP 162
LpMenG2          LSFLLSEKVGVNGKVIALDFSKEQLQIAASRQRERSRSKPCYKNIEWIEGDAVALPFPDSTFDAATIGYGLRNVVDRKKCLEEMVRVLKP 162
TvMenG2          LSFLFSQTVGVNGKVTALDFSSDQLQVAKSRQHERSKSNPCYKNIEWIEGDAVALPFPDSTFDAATIGYGLRNVLDRKKALEEMVRVLKP 164
ShMenG2          LSFLLSLKVGIDGKVIALDFSKELLEIAASRQREWSKSKPCYENIEWIHGDAVALPFPDSSFDAATISYGLRNVVDRKKALEEMVRVLKP 162
PaMenG2          FSFRLAEKVGINGKVIGLDFSKELLQVAACRRRDQSSSKPCYNNIEFIEGDAVALPFPDSAFDAATIGYGLRNVIDRKKALEEMVRVLKP 164
CaMenG2          LSFLMSEKVGS-GKVNALDFSKEQLHIAASRQHERLNSKPCYKNIEWFEGDAVALPFSDTIFDAATIGYGLRNVVDKKKAMEEMVRVLKP 166
AfMenG2          LSFLLSEKVGINGKVTSLDFSKEQLKIAASRQRERSKSKPCYNNIEWTEGDAVALPFSDSTFDAATIGYGLRNVIDRKKALEEMVRVLKP 162

At1g23360        GSRVSILDFNKSNQSVTTFMQGWMIDNVVVPVATVYDLAKEYEYLKYSINGYLTGEELETLALEAGFSSACHYEISGGFMGNLVAMR 261
Potri.008G188600 GSKASVLDFNKSTQPFVASFQEWMIDNAVVPVATAYGLAKEYEYLKGSIREFLTGNELEELALEAGFSTAKHYEISGGLMGNLVATR 262
Solyc12g019010   GSTLSVLDFNKSINPLSTTVQEMMIDNIVVPVASGYGLENEYKYLKSSIKDFLTGNELEKLALEVGFSTAEHFEIGFGFMGNLVAIR 256
Migut.E00183     GSKVSVLDFNKSTSPLTSSLQDWMIDNVVVPVANGYGLASEYRYLKNSIKEYLSGNELEKLAFEAGFSEGKHYEIDAGFMGNLVATR 259
VtMenG1          GSKLSILDFNKSSSPLTSTLQDWMIDFVVVPVASGYGLASEYQYLKNSIKEYLTGKELEKLALEAGFCDAKFFEIEAGLMGNLVATR 256
RgMenG1          GSKVSVLDLNKSTNPLTSSIQDWXIDYAVVPIANGYGVASDYQYLKNSIKEYLTGNELEKLALEAGFSKAKHYEIGAGFMGNLVATL 259
LpMenG1          GSKLSVLDFNKSTSPLTSSIQDWMIDNVVVPVASGYGVASDYQYLKNSIKEYLTGNELENLALEAGFSEATHYEIGGGLMGNLVATL 259
ShMenG1          GSKLSVLDFNKSTNPLTSSVQDWMIDYVVVPVASGYGLASDYKYLKNSIKEYLTGDELEKVALEAGFSRARHYEICGGLMGNLVATL 259
TvMenG1          GSKLSVLDFNKSTNPLTSSIQDLMIDYVVVPVADGYGVASDYKYLKNSIQEYLTGNELEKLALEAGFSRAKHYEISGGLMGNLVATL 259
VtMenG2          GSKLSVLDFNKSTSKSMCKFQELVLDYIVVPIASWYGLRDEYHYLNKSIKEYMTGNELEKFALEAGFSEAKYYEIAGGAMGNLVATR 251
RgMenG2          GAKLSVLDFNKSTNWLNIKIQEWMIDYIVVPVASWYGLASEYRYLKNSIKEYLTGPELEKLALEAGFSEAKFYEISNGSMGILVAKR 249
LpMenG2          GAKVSVFDFNKSTSWLTSKIQDWMIDYIVVPVASWYGLASEYRYLKNSIKGYLTGKELEKLALEAGFSEAKHYEVAGGSMGNLIATR 249
TvMenG2          GAKLSVLDFNKNTSQVMCKIQDWMIDYVIVPVASWYGLASEYRYLKNSIKGYLTGSELEKLALEAGFSEAKHYPIAGGAMGNLVAKK 251
ShMenG2          DAKLSVLDFNKSTNRLTSKIQDWMIDYIVVPVATWYGLASEYRYLKNSIKEYLTGKELEKLALEAGFSEAKHYEIACGSMGNLVATR 249
PaMenG2          GAKLSVLDFNKSTHWLNIKIQEWMIDYVVAPVASWYGLESEYRYLKISVKEYLTGSELEKMALEAGFSVAKFHSIAGGVMGNLVATR 251
CaMenG2          GAKLSVLDFNKSNNWLNSKIQDWMIDYVVVPVASWYGLANEYRYLKNSIKEYLTGKELEKLALEAGFSEAKHYEIAGGYMGNLVAIR 253
AfMenG2          GAKLSVLDFNKSTNWLNSKIQDWMIDYVVVPVASWYGLTSEYRYLKNSIKEYLTGTELEKLALEAGFSVAKHHAIAGGSMGNLVATR 249
```

Supplemental Figure S5. MenG sequence alignment.

MenG sequences from the three parasitic plants, representative non-parasitic plants from Phytozome and additional species from 1KP (Supplemental Dataset S1) are shown. Af, *Aphyllon fasciculata*; Ca, *Conopholis americana*; Rg, *Rehmannia glutinosa*; Lp, *Lindenbergia philippensis*; and Vt, *Verbascum thapsus*.

```
Potri.011G033100 MAVKIYIIYYSMYGHVARLAEEIKKGADTVEGVEIKLWQVPETLPEEVLGKMGAPPK-SDVPIIKPNDLTEADGVLFGFP 79
Migut.M01839     MATKVYIVYYSMYGHVEKLAEEIKKGAASVEGVEAKLWQVPETLQEDVLTKMSAPPK-SEVPIITPSELAEGDGFIFGFP 79
AT5G54500        MATKVYIVYYSMYGHVEKLAEEIRKGAASVEGVEAKLWQVPETLHEEALSKMSAPPK-SESPIITPNELAEADGFVFGFP 79
AT4G27270        MATKVYIVYYSMYGHVEKLAQEIRKGAASVDGVEAILWQVPETLQEDVLSKMSAPPK-SDAPIITPNELAEADGFIFGFP 79
Potri.001G410700 MATKVYIVYYSMYGHVEKLAEEIKKGASSVEGVEAQLWQVPETLPEEVLGKMSAPPK-SDVPIITPGELAEADGFVFGFP 79
Potri.011G129400 MATKVYIVYYSMYGHVEKLAEEIRKGASSVEGVEAKLWQVPEILPEEVLGMMSAPPK-SDVPIITPSELAEADGFVFGFP 79
TvQR2            MATKVYIVYYSTYGHVERLAQEIKKGAESVGNVEVKLWQVPEILSDEVLGKMWAPPK-SDVPVITPDELVEADGIIFGFP 79
ShQR2            MAAKVYIVYYSTYGHVERLAQEIKKGVESIPEVEAKLWQVPETLTNDILAKMGAPPKNNDVPVISPNELVDADGIIFGFP 80
PaQR2            MATTLSIVYYSTYGHVEKLAQEIKKGAESVEGVEAKLWQVPETLSDEILGKMGAPPKSSEVPVITPDELAEADGFIFGFP 80
Migut.M01245     MATKVYIVYYSTYGHVERLAKEIKKGAESVEGVEAKLWQVPETLSDEILSKMGGPPK-SDVPIITPNELVEADGILFGFP 79

Potri.011G033100 TRFGMMAAQFKAFLDATGGLWGTQQLAGKPAGIFFSTASQGGGQETTALTAITQLVHHGMIFVPIGYTFGAGMFEMEQVK 159
Migut.M01839     TRFGMMAAQFKAFFDATGGLWRAQQLAGKPAGIFYSTGSQGGGQETTALTAITQLVHHGMIFVPIGYTFGAGMFEMEKVK 159
AT5G54500        TRFGMMAAQFKAFLDATGGLWRAQALAGKPAGIFYSTGSQGGGQETTALTAITQLVHHGMLFVPIGYTFGAGMFEMENVK 159
AT4G27270        TRFGMMAAQFKAFLDATGGLWRTQQLAGKPAGIFYSTGSQGGGQETTALTAITQLVHHGMIFVPIGYTFGAGMFEMENVK 159
Potri.001G410700 TRFGMMAAQFKAFLDATGGLWKTQQLAGKPAGIFFSTGSQGGGQETTALTAITQLVHHGMIFVPIGYTFGAGMFEMEKVK 159
Potri.011G129400 TRFGMMAAQFKAFLDATGGLWKTQQLAGKPAGMFFSTGSQGGGQETTALTAITQLVHHGMIFVPIGYTFGAGMFEMEKVK 159
TvQR2            TRFGMMAAQFKAFFDSTGGLWKTQALAGKPAGIFFSTGTQGGGQETTALTAITQLTHHGMIYVPIGYTFGADMFNMEKIK 159
ShQR2            TRFGMMAAQFKAFFDSTGGLWRTQALAGKPAGIFYSTGSQGGGQETTPLTAITQLTHHGMIFVPIGYTFGAGMFEMGEIK 160
PaQR2            TRFGMMASQFKAFFDSTGGLWRTQALAGKPAGIFYSTGSQGGGQETTALTAITQLTHHGMIFVPIGYTFGAGMFEMEKIK 160
Migut.M01245     TRFGMMAAQFKAFIDSTGGLWRTQALAGKPAGIFYSTGSQGGGQETTPLTAITQLTHHGMIFVPIGYTFGAGMFEMGEIK 159

Potri.011G033100 GGSPYGAGTFAG-DGTRQPTELELGQAFHQGKYFAGIAKKFKGTT-- 203
Migut.M01839     GGSPYGAGTFAG-DGLRQPSDLELEQAFHQGKYIATITKKLKGSA-- 203
AT5G54500        GGSPYGAGTFAG-DGSRQPTELELQQAFHQGQYIASITKKLKGSTA- 204
AT4G27270        GGSPYGAGTFAG-DGSRQPTELELGQAFHQGKYIAAISKKLKGPAAA 205
Potri.001G410700 GGSPYGAGTFAG-DGSRQPTELELEQAFHQGKYIAAITKKLKGAA-- 203
Potri.011G129400 GGSPYGAGTFAG-DGSRQPTELELEQAFHQGKHIAAITKKLKGAA-- 203
TvQR2            GGSPYGAGTFAGADGSRQPSDIELKQAFHQGMYIAGITKKIKQTSA- 205
ShQR2            GGSPYGAGTYAG-DGSRQPSGIELEQAFHQGKHIAAITKKLKQTT-- 204
PaQR2            GGSPYGAGTYAG-DGSRQPSDIELAQAFHQGKYIAAITKKLKKSV-- 204
Migut.M01245     GGSPYGAGTYAG-DGSRQPSDLELAQAFHQGKYIAGITKKLKTSAA- 204
```

Supplemental Figure S6. QR2 sequence alignment.

QR2 sequences from the three parasitic plants and representative non-parasitic plants from Phytozome are shown.

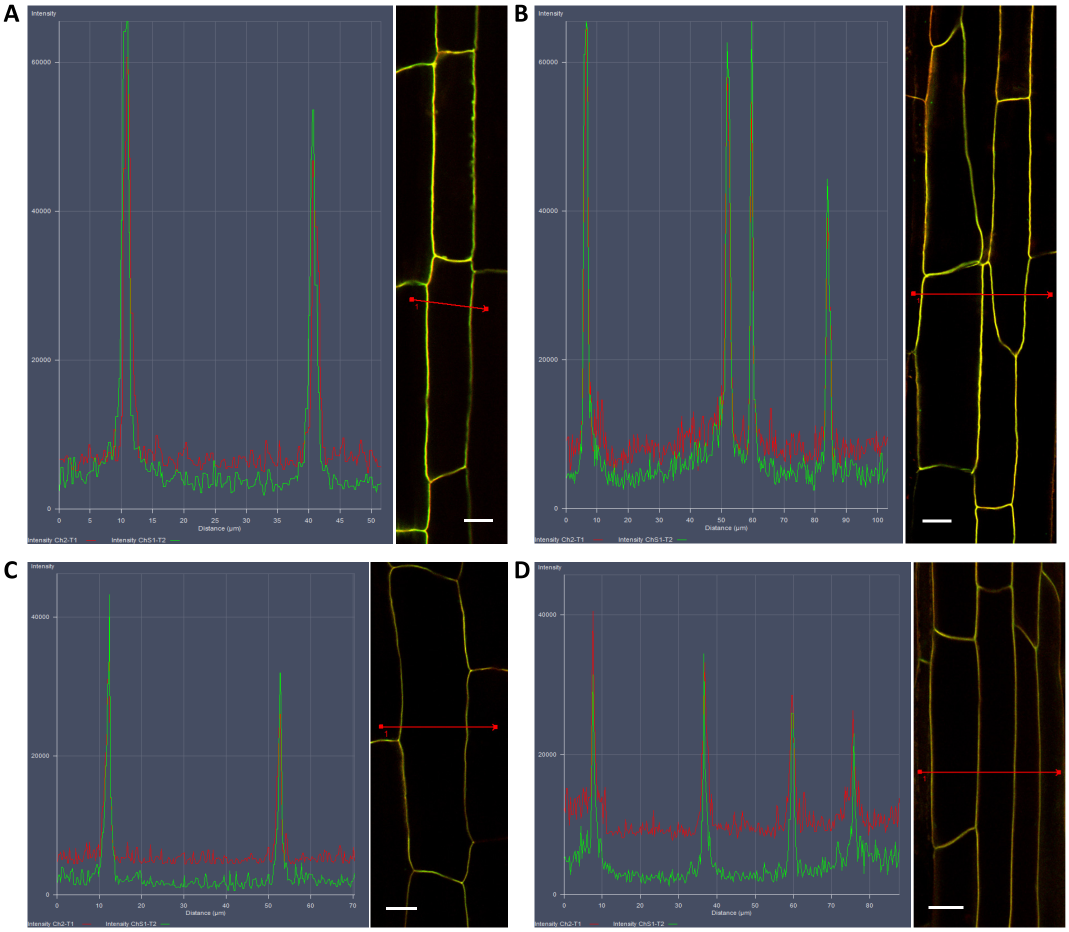

Supplemental Figure S7. The signal intensity profiles of plasma membrane marker AtPIP2A with PaMenA2 or PaMenG2.

The intensity profiles of AtPIP2A-mCherry in the red channel (A-D) in conjunction with PaMenA2-GFP (A-B) or PaMenG2-GFP (C-D) in the green channel from roots of two independent transgenic events each. Within each panel, signal intensities were recorded along the red arrow shown on the image, which corresponds to the x-axis of each profile. Scale bars = 20 µm.

Supplemental Table S1. Plastid-targeting prediction for ICS, PHYLLO and NDC1

	Predotar	TargetP	Protein Prowler	WolF PSORT	Prediction
ICS					
AT1G18870*	0.79	0.95	0.97	13	plastid
AT1G74710	0.82	0.77	0.99	11	plastid
Potri.012G070000	0.21	0.27	0.07	9.5	plastid
Solyc06g071030	0.11	0.52	0.08	5	plastid
Migut.I00130	0.70	0.16	0.08	10	plastid
Migut.I00129	0	0.03	0	13	plastid
TvICS	0	0.18	0	4	-
ShICS	0.01	0.30	0.07	6	-
PaICS	0.18	0.22	0.03	8	plastid
PHYLLO					
AT1G68890*	0.37	0.94	0.89	12	plastid
Potri.010G135500[1]	0.07	0.58	0.92	14	plastid
Solyc04g005190[1]	0.24	0.46	0.31	12	plastid
Migut.L01140	0.20	0.64	0.31	13.5	plastid
TvPHYLLO	0.37	0.45	0.89	11	plastid
ShPHYLLO	0.57	0.74	0.98	12	plastid
PaPHYLLO	0.63	0.40	0.28	13	plastid
NDC1					
At5g08740*	0.4	0.9	1.0	11.5	plastid
Potri.007G098700	0.6	0.8	1.0	11.5	plastid
Solyc03g043750	0.9	0.9	0.9	13.5	plastid
Migut.L00271	1.0	0.9	1.0	12	plastid
Migut.L00582	1.0	0.9	1.0	12	plastid
TvNDC1	1.0	0.8	0.8	13	plastid
ShNDC1	1.0	0.7	1.0	14	plastid
PaNDC1	0.9	0.9	1.0	12	plastid

Heatmaps show prediction strengths for scores above the 50th percentile of each method.
*Experimentally verified for plastid-targeting.
[1] GenBank accession numbers XP_024466568 (poplar) and XP_004237229 (tomato) were used due to erroneous gene model annotation in the reference genomes.

Supplemental Table S2. List of primers

Primer tails (overlapping sequences) for Gibson assembly are underlined.

Gene/vector	Primer sequence (5'-3')	Purpose
PaMenA2	GGACCAGATCCCAAGCAAATTA	RT-PCR cloning
PaMenA2	GTTGTAAAACGACGGCCAGTGAATTCGTAGAGTGTGCAAAGACTCRGA	RT-PCR cloning, pUC19 tail
PaMenA2	CTATGACCATGATTACGCCAAGCTTGGACCAGATCCCAAGCAAATTA	RT-PCR cloning, pUC19 tail
PaMenG2	TTAACAARATCCACTGGATCG	RT-PCR cloning
PaMenG2	CTATGACCATGATTACGCCAAGCTTGAGACCGAAAAKCGAAAATGGC	RT-PCR cloning, pUC19 tail
PaMenG2	GTTGTAAAACGACGGCCAGTGAATTCCAAACACGAACRCAAAGCA	RT-PCR cloning, pUC19 tail
pUC19	AAGCTTGGCGTAATCATGGTCATAG	pUC19 vector
pUC19	GAATTCACTGGCCGTCGTTTTACAAC	pUC19 vector
PaMenG2	TCTAGAAGGAGGATTACAAAATGGCTACAGCTACACTTAG	pUCM cloning, vector tail
PaMenG2	GTTGTAAAACGACGGCCAGTGAATTCCTAGCGCGTGGCGACCAAATTTC	pUCM cloning, vector tail
EcMenG	TCTAGAAGGAGGATTACAAAATGGTGGATAAGTCACAAGAAAC	pUCM cloning, vector tail
EcMenG	GTTGTAAAACGACGGCCAGTGAATTCAGAACTTATAACCACGATGC	pUCM cloning, vector tail
pUC19	CATTTTGTAATCCTCCTTCTAGACGCTCACAATTCCACACAAC	pUCM vector
pUC19	GAATTCACTGGCCGTCGTTTTACAAC	pUCM vector
GFP	AGAAGACTGAAGTTAGTAGCTCGAGATCTGAGTCCGGACT	PaMenA2-GFP-2A-HPT cloning, 2A tail
HPT	GCTACTAACTTCAGTCTTCTGAAGCAGGCTGGAGATGTGGAGGAGAACCCTGGACCAATGAAAAAGCCTGAACTCACC	PaMenA2-GFP-2A-HPT cloning, 2A tail
GFP	ATGGGTAAAGGAGAAGAACTTT	pCX-AtMenA.1-GFP cloning
GFP	GAACGATCGGGGAAATTCGCTATCTGGCTTTTAGTAAGCCCC	pCX-AtMenA.1-GFP cloning, pCXSN (NosT) tail

Supplemental Table S3. Accession numbers

Species	Gene	Accession number	Database
Arabidopsis thaliana	CSD1	AT1G08830	TAIR v10 https://www.arabidopsis.org
	CSD2	AT2G28190	
	DHNAT1	At1g48320	
	DHNAT2	At5g48950	
	FRO4	AT5G23980	
	FRO5	AT5G23990	
	FSD2	AT5G51100	
	FSD3	AT5G23310	
	GPX4	AT2G48150	
	GPX5	AT3G63080	
	GPX6	AT4G11600	
	GPX8	AT1G63460	
	ICS1	At1g74710	
	ICS2	At1g18870	
	MenA	At1g60600	
	MenB	At1g60550	
	MenE	At1g30520	
	MenG	At1g23360	
	NDC1	At5g08740	
	PHYLLO	At1g68890	
	QR2.1 (FQR1)	At5g54500	
	QR2.2	At4g27270	
Glycine max	ICS1	Glyma.01G104100	Phytozome v12 https://phytozome.jgi.doe.gov
	ICS2	Glyma.03G070600	
	PHYLLO	Glyma.15G276800	
	MenE1	Glyma.09G024500	
	MenE2	Glyma.15G130500	
	MenB1	Glyma.08G285500	
	MenB2	Glyma.18G139700	
	DHNAT1	Glyma.18G260800	
	DHNAT2	Glyma.09G236500	
	MenA1.1	Glyma.08G070600	
	MenA1.2	Glyma.13G042800	
	NDC1	Glyma.05G195400	
	MenG1.1	Glyma.01G028200	
	MenG1.2	Glyma.02G037000	
	QR2	Glyma.13G284200	
	NQR1.1	Glyma.04G140400	
	NQR1.2	Glyma.11G152400	

Mimulus guttatus	MenA	Migut.B00584, Migut.B01155, Migut.B01157	Phytozome v12 https://phytozome.jgi.doe.gov
	MenG	Migut.E00183	
Solanum lycopersicum	SlMenA	Solyc01g105460	Phytozome v12 https://phytozome.jgi.doe.gov
	SlMenG	Solyc12g019010	
Populus trichocarpa (P. tremula x *alba)*	ICS	Potri.012G070000	Phytozome v12 https://phytozome.jgi.doe.gov
	PHYLLO	Potri.010G135500	
	MenE	Potri.011G164100	
	MenB	Potri.001G329900	
	DHNAT1	Potri.010G003600	
	DHNAT2	Potri.010G003800	
	MenA	Potri.017G050900	
	NDC1	Potri.007G098700	
	MenG	Potri.008G188600	
	QR2.1	Potri.001G410700	
	QR2.2	Potri.011G129400	
	QR2.3	Potri.011G033100	
Phtheirospermum japonicum	QR2	QIH44907	NCBI/EMBL
Phelipanche aegyptiaca	PaMenA2	MT506520	NCBI/EMBL
	PaMenG2	MT506521	
Phelipanche ramosa	PRX1	AAY89058	NCBI/EMBL
	POX1	AAU04440	
Striga asiatica	POXA	AAB97853	NCBI/EMBL
	POXB	AF043235	
	NOX1	ABG35770	
Triphysaria versicolor	QR1	AF304461	NCBI/EMBL
	QR2	Q9AYU0	
E. coli	MenA (UbiA)	P32166	NCBI/EMBL

Supplementary Dataset S1. PLAS-assembled or 1KP-derived and manually curated transcript sequences of phylloquinone biosynthetic and coexpressed genes described in the manuscript.

```
>PaICS Phelipanche aegyptiaca Pa.c23458_g1_i4.7758
GTTACTACTACTCACATTCCCATAACATATGCAACCCAAATAGAAAAGAAAAAAGATATACACAACTCACACTAGCGAGCGTACAT
GGCTACTACTCTTAAGCAATACTATTTCAATGCCGGCAGTTATAAGGACATTGGATCCAAGAAACGCTTTCCCTCATCCCCCACTG
CAATTGCCAACCCGTCGGTTAATTTCTACAACCATAAACATCAAGAACTCGGTTCGTTGTCCATGAATGGTTGTGGAGGTGATCCA
AGAGCTCCCATTGGCACCATCGAGACTCGGACACTTCCGACAGAACCGACAATGGCACTGGCGGCCGACCGCCTCAACTCCGCCAT
ATATGGCCTCAAATCCGATGCTCCGTCGTTCGACTCAGGGATTATTCGAATCGAGGTACCAATTGGAGAGCAGATAGAGGCCCTTG
ATTGGCTTCGTTCACAGAGCCATAGCCATCTTCTTCCCCGCTGCTTTTTCTCAGGCCGAGATTCCAACAATATCACACCACAAATT
AATGGAAACGGAAATGGGGTTATTAACGGCTATCACTCGTCTTCTTCTCAACAAAAACAGAAGCTTGTTAGTGTTGCCGGCCTGGG
CTCAGCCGTCTCCTTCCGCCATCTTCATCCTTTCTCTTTGGACGATTGGCATTCCATCAAAAGGTTCTTATCCAAAAGGTGTCCAT
TGATTCGTGCTTATGGCGCTATGCGATTTGACGCGAGATCCGTTATAGCCCCTGAGTGGAGGGGCTTTGGTTCATTTTATTTTATG
GTTCCTCAGGTTGAGTTTGATGAGTTTGAAGGAAGTTCAATGATAGCTGTAAATGTTGCATGGGACAATCGCCTGTCACAGTCCTA
CGAACAAGCAATTGCTGCGGTTCAAGCTACAATGTCTAAGATTTCAAGAGTTGTCCGGAGAACAAATGGTAGCTCACCTCGTGCAG
TTCTACTGCATCAGACTCATGTTCCCAACAAAGCGTCATGGGATTCATCTGTCAAGCAAGCTTTGGACTTGATAAGCAGGAAAGAC
TCGAGGCTTATTAAGGTTGTTCTAGCACGTAGCAGCAGAGCACTAACCACCGTTGAGATCGACCCTTTAGAGTGGTTATCAAGCTT
GCAGGTTGAAGGGGTTAATTCTTACCAGTTTTGTCTTCAGCCACCTGAATCCCCTTCATTCATCGGGAACACTCCTGAGCGACTAT
TCTACCGAGACCGACTTAGCGTCTGCAGTGAGGCTTTGGCTGGAACACGAGCTAGAGGAGGAACCGAGTCACTTGATCTGCAGATA
GGAAATGATTTACTTTCAAGTGCTAAAGACCATCATGAATTTGCTGTTGTAAGAGAGAGCATAAGAAGAAAGTTAGAGAATGTATG
CTCTAGCACAATAGTTGAACCAAGCAAAACTCTACGAAAACTCCCACGTGTTCAACATCTTTATGCTAAGCTGACGGGGACATTGC
AGAAAGAAGAAGACGAGTTTAAGATTCTGTCTTCTCTTCATCCGACTCCTGCTGTTTGTGGGCTACCTACGGAAGATGCACGGATT
TTAATTTCCGAAACCGAAAGGTTTGACCGAGGAATGTATGCTGGTCCTGTTGGGTGGTTTGGTGGTGCAGAGAGTGAGTTTGCTGT
TGGGATAAGATCGGCATTAGTTGGGAAGGGTGTTGGTGCATTACTGTATGCTGGAACTGGGATAGTAGAAGGAAGCGACTCGGGCC
TCGAATGGCAGGAGCTCGAACTCAAGACTTCACAGTTCACCAGATTGATGAAACTCGAGGCAACACCTCTTCCGGCAATGGGGAAG
AAAAATTGAAACGTGATGTAAAATAAGGGCTTAAAAACCGGGCGAGCTTACTTGCGAAAATCTCAATGTCCAGTTGCTTCATATGC
TGTCCGGTGCACTGTCTACCATCATCAATATATATTTCTAAGAAATAAAATTAATACTACTACCATGTAATTAGGCATAAGGGAAA
ATAGGTCCTCAATCTCAATGTAAAATATCTGTGTTTTGTACAAAAATATTATTTGTTCAGTAAAGGTTTTCTTCTTGAGCAATAGC
CAATACGGTTTACGTTGTTGGTTGAACGTTTAAGGATTAAATTAAAGTTTTGTGTGATAGGATTAAATTTTGGCTTTAAAATTTCT
GTGGCTCAGTATGGGAAATTGACCAATAAATATAAATTGTTTCCCACCAAAAAAAAAAA
>ShICS Striga hermonthica Sh.c17992_g1_i8.612
CCACACACACACACACACACACACATTAAAGCACAAATGTCCATTATAAGGCATTTCAATGCAAGCTTCAAGGACATTAAGTCCAAGA
AGTGCTTTTTCTCAACCCCAACTGCAATTGCAAATGCATCACTTCAATTCTCCAATTATAAACCTCAAGAGCTTTGTTCCTTGTCA
TTGGATGGGTGTCGAGGCCTCGACCCGCGGGCCCCACTTGGGACGATTGAGACTCGAACGCTCCCCAAGGCCCCGACTCCCGCTCT
GGCGGCCGACCGACTCAACTCTGCAGTCTATCGTCTCAAATCCGACGAGCCGCCTTCGGAATCCGGGATTATTCGGATTGAGGTAC
CCATCGCAGAGCCGATAGAGGCGCTTGACTGGCTTCGTTCGCAGAGCCACGGCCATCTTCTTCCTCGCTGCTTTTTCTCCGGCAGA
GATACGGCCAACACCTCACTAATCGAGCATGTTAACGGCAACGGCAATGGGATTAACGGCCACTCATCTCCTCAACGGAAACTTGT
CAGTGTTGCTGGCCTCGGCTCAGCCGTCTCCTTCCGCCATCTCCGACCGTTCTCTTTGGACGATTGGCATTCGATTAAAAGGTTTG
TATCCAAAAACTGTCCGTTGATTCGTGCTTATGGTGCAATGCGCTTTGATGCGAGATCTAAGATATCCCCTGAGTGGAATGGTTTT
GGTTCCTTCTACTTTATGGTCCCTCAGATCGAGTTTGACGAGTTTGAAGGAAGTTCAATGATAGCTGCAACTGTGGCGTGGGATAA
TCGTCTTTCACTTCCCTACGAACAAGCAGTTGCAACACTCGAAGCTACACTGTCCGAGGTTTCAACAGTCGTTTGGAAAATAAATG
ATAGCTCTCATCGTGCTCCTGTCCTTCACCAGACTCATGTTCCCAACAAAGCCTCGTGGGATCAAGCTGTTAAACGAGCTCTTGAC
TTGATAAGCAGTAAAAACTCCTCGCTTGTTAAGGTTGTGCTTGCGCGTAGCAGCAGAATTCTGACCACGGTTGAGATTGATCCTTT
AGAATGGTTATCGAGCTTGAAGGTTGAAGGGGCTAACTCGTACCAATTTGTTCTTCAGCCACCTGAATCCCCTGCATTCATTGGGA
ACACACCTGAGAGACTGTTTTACCGAGACCGTCTAAGCGTATACAGTGAGGCCTTGGCCGGAACTCGAGCTAGAGGAGGAACAGAA
TCACTAGATTTACAAATAGGACATGATCTACTCACCAGCCCTAAAGATCACCATGAATTCGCTGTCGTAAGAGAGAGTATAAGAAG
AAAATTCGAGAACGTGTGCACAAGCACTGTAGTTGAACCGAGCAAAGCTCTAAGGAAACTGCCACGTGTCCAACATCTTTATGCTA
AGCTAACGGGCACATTGCAGAAAGAAGATGACGAGTTTAAGATTCTGTCTTCACTTCATCCAACCCCTGCTGTTTGTGGGCTTCCA
ACTGAAGATGCACGAATTCTAATATCACAAACTGAAATGTTTGACCGAGGAATGTATGCTGGCCCGGTTGGATGGTTTGGTGGTGC
TGAGAGTGAATTTGCCGTCGGAATAAGATCAGCATTAGTAGGAAAGGATATCGGGGCATTACTTTATGCTGGGACCGGAATAGTAG
AAGGAAGCAACTCTTCCCTCGAGTGGAAGGAACTCGAACTCAAGACATCTCAGTTTACGAAATTGATGAAACTCGAGGGGCCTCTA
CCAACAATAAGGGAGAAAGTTGAAATGTAACTCAGAAAAGAAAAGTATATACTTTTCTTCCAGAAGTATATATAGATTTGCTCAGA
GTGCAGCTTGTTTAAGTGATGCAGACCCTAGTAAGATATTGTCCGCTTTGGGCTACTTCCGCCACCCGCACGGCTTTGTTCTTGGC
TGGGCTTCAATACACACCCACCAAAACGCGTCTTACTAGTGAAGGTATCCACATCCTCATATAAGGCCTGCTTCGTTTCCTTTCAA
ACCCGATGTGGGATGCGCTGCATCATCCACCCCCCCTTGTGGGCCCAGCGGTCACCGCTGGCACATGGGCTGGCTCTGATACCAAC
>TvICS Triphysaria versicolor Tv.c7349_g1_i2.6356
CTCAAATATTATAGTACAAAACAAGAGAAGAAAATAATCACACACTAGACTACACATGTCTACTACTAAGTATTTCAATGCTGGTT
TCAAAGACATTGAGTCCAAGAAATTCATTTTCTCATCCCCGATCGCAATTGCAAAACCATCACTTCATTTCTCTAATCATAAATAT
GAACTTGGTTCATTGTCCATGAATGGATGTGGAGGCGATCCACGAGCCCCCATTGAAAAAATCGAGACTCGAACGCTTCCGATTGC
TCCGACGCCGGCATTGGCGGCTGACTGCCTCAACTCCGCCATCTACCACCTCAAGTCGAATGCTCCGGCGGCCGATTCCGGGATTA
TTCGGATTGAGGTACCAATCCAGGAGCAGATAGAGGCGCTTGATTGGCTTCGTTCACAAAGTCACACCCATCTTCTTCCTCGCTGT
TTCTTCTCGGGCCGAGATTCCAACAATATCCCACCACTCAATCAACACATCAATGGAAATGGAAATGGGAATGGGATTAATGGCCA
```

TTCTTCTGCTAAAGAACATCAACAGAAGCTTGTCGGTGTTGCCGGGCTGGGCTCAGCTGTCTCCTTTAGCCATCTCCACCCTTTCT
CTTTGGACGATTGGCATTCCATCAAAAGGTTTTTATCCAAAAATTGTCCATTGATTCGTGCTTATGGCTCAATTCGGTTCGACGCG
AGATCCAATATAGCCTCCGAGTGGAATGGTTTTGGATCCTTTTATTTTATGGTCCCTCAGATTGAGTTCGATGAATTTGAAGGAAG
TTCAATGATAGCTGCAACAGTTGCATGGGACAATCGTTTATCACGGTCCTACGAACAGGCAATGGCTGCACTCGAGGCTACAATGT
CTAAGGTATCATCGGTTATCCGGAGATTAAATGGTAGCTCACATCAGGCTGTTGTGCTCCACCAGACTCATGTTCCCAACAAAGCC
TCCTGGGATTTGGCTGTTAAACGAGCTTTGGATTCGATAAGAAGTAAAAACTCACCGCTTGTTAAGGTTGTTCTTGCACGTAGCAG
CAGAAATTTGACCACGGTCGAGATTGACCCTTTAGAGTGGTTGTCGAGATTGCAGGTCGAAGGGGTTAATGCCTACCAGTTTTGTC
TTCAACCACCTGAATCCCCTGCATTCATCGGAAACACTCCAGAGCGACTATTCTATCGAGACCGACTAAGTATATGGAGTGAGGCT
TTAGCCGCAACACGAGGTAGAGGTGCAACCGAGTCACTCGATTTACAGATCGGAAACGATTTACTTTCAAGTGCTAAAGACCATCA
CGAATTTGCTGTTGTACGAGAGAGTATAAGGAGAAAATTTGAGAAGGTGTGCTCAAGCACAGTAGTAGAACCGAGCAAAGCTTTAA
GAAAACTACCACGTGTTCAACACCTTTATGCTAAGCTCTCGGGCACATTACATAAAGAAGATGACGAGTTTAAGATTTTGTCTTCT
CTTCATCCAACTCCTGCTGTTTGTGGGCTTCCTACAGAAGATGCGCGGGTTTTAATATCGGAAACTGAAATGTTTGACCGAGGAAT
GTATGCTGGCCCGGTTGGTTGGTTTGGTGGTGCCGAGAGTGAGTTTGCTGTCGGAATAAGATCCGCATTAGTTGGGAAGGGTGTTG
GCGCGTTACTGTATGCGGGAACGGGAATAGTAGAAGGAAGCAATTCGTCCCTGGAATGGCAAGAACTCGAACTTAAGACATCACAG
TTTGCAAAATTGATGAAACTTGAGGCTCCTCTTCAAGTGACGAGGGGAGAAAAATGATCTGTGACTTAAGGTTAGTATAAGCAATG
AGTTACTCATCTGCATTTGTGTAGCTTATTCTACAGTGCCAAAACCCAATATATATATTTCTCACTTGTGATGAAAAGTCAGATGT
CAATTCTTGTATACAGCTGGTTTGTAATCATCCATTGTTAGATAAACTCTCTATATTGTATATATAGAGAGAGATGGGCCTGAGGA
AACAATATTTTTTATTATCATTCTTTTTTTTTTTCGTTACCAATGCTTTGTACATAATTTTGTTTGGTTCCTATCCTGTTGTGATA
TAATATGTAGAAATTATAAAGGGGACTCACG

>PaPHYLLO Phelipanche aegyptiaca Pa.c19994_g1_i1.144
GCGAGATAAGTATGTATTATATATTGAACCAGCAACTGTCATTTCTTTAGGACTTAGCGCCTTCTCCTCTTTGAACCTCCATTACC
AATGATTCCCTTCACCTTATACTCTCAAATTACTCCACCTTTTCCATTATCTCCAATCCAAACCCACCAAAAGACCATAATAACAA
TTACTAAGCGCGCCCATTTTCTGAAACCTGCTTTGCCATGTCTAGCTCGCTTTGGCCACCGTCAAAATCCCATTTCCAAGGTTGTG
AGTAGCTCAATGGGAAAAGATAAAGTGTTGGATGCTAAACATGCTGCATTGCTGATCAATACTTGCATTACGCGTAATTTGCCGCC
GGTTTTGAGTTTAGAGCAAGGACTGGATAGGATTAAGGAGGATTTGGAGGAGTTAAAGGCTAATCGTCCTCCTTGTTCAAGTGGGA
TGTACAGATTCCAGCTTGCAGTGCCTCCAAGTGCAAAAGCGTTGAACTGGTTTTGCTCTCAACCGGAGTCATCAAACGTCTTTCCT
CTATGCTTCCTCTCCAATGAGGACAATCCAACATATAATTCACTTTCTCTTGGAAGAACCAGGGGTGTTTTTGGAATTGGTTCTGC
TGTTGTTTTCAAGGATCGTTCTCATGCTTCAGAAAATAGCAGAGCTGTACGAAGATGTCTTTCGGCTGAACCAACATCTTCTAAGG
CTTATGGTTTCTTGGATATCGAGTTTGACAGTAACATGTCTACTACAAAGCATCAGATCGGTTCATATTACCTTTTCATTCCCCAG
ATTGAGTTGGATGAATTTGAAGATATCCCTTTCTTGATTGCAACATTGGCATGGGATGACTCTTCAATTTGTACATTTAGTGAAGC
TGTTCAAAGATTTGAGCTCGCTTTTGATCAGGCCAGATACACCTGTGGAAACGGTAGCCAATTGATTAGATCTTCTCTTTTAAAGT
TCAGCAATGCAGAGAAACATACGGAAATGGTTCGTGCAAATGGTCTACTATTGGATGGGCAACATCTTACAGCCAGCACCATGGAA
ACGGAAGATGCTTCGTCTTGTTGCAGACAGTTTGTTGCAAGACTTTCATCAACTTTATCGATTTCAAATAACATGCTCCCAAGAGA
CGAAACCAAATTAATCGAAAATGTCACTCAAGATTTTCCCAATATCAATACTTTGTGGGCATACCTTATAGTTGAAGAATGCACCC
GTCTTGGTTTGACATATTTCTGTATAGCTCCCGGATCAAGGTCATCTCCACTGACGATTGCTGCAGCCAGTCACCCCCTTACGACT
TGCATTGCATGCATTGATGAACGATCCCTAGCATTTCATGCTCTCGGTTATGCCAAAGGTTCCGGAAAACCAGCAGTTGTTATAAC
ATCATCAGGGACAGCTGTCTCAAATCTTTTTCCGGCCGTTGTGGAGGCTAGCCAGAGTTTCGTACCAATGCTGTTGCTTACTGCCG
ACCGTCCTCTTGAGCTCGTAGATGTTGGGGCGAACCAAGCAATCAATCAGGTCAATCATTACGGATCATTCGTGAGGCACTTTTTC
AGCCTTCCTCCACCTGCCGATGACATATCCGCAAAATTCGTTCTTACCACAATCGACTCAGCAGTATGCAAATCAACATCTTCACC
AAACGGTCCTATACATATTAACTGCCCCTTTCGAGAACCGCTAGCACACAGTCCAAGAAATTGGGACCTTAAATGTTTAAGTGGAT
TAGACGTTTGGATTTCAAATGCCAAGCCGTTTACCAGTTACATTCCATTACAACATTCCCTAACGTGTAATAACCCGAACGGGAAT
ATGATTGAAGTGTTGAAACTAGTCCAAGGGGCCAATAATGGGATTTTAGTTTTGGGTTCGATTCACAAAGAGGATGATATGTGGGC
AGGTCTTTTGTTGGCTAAGCACTTGTCGTGGCCGGTTGTTGTTGATATCCAGTCGGGTTTAAGATTGAGGAAGCACTTGTCGTCGT
TTCATGAGAGAAAAGATATATTGTTCATTGATCAGCTTGATCAACTGTTGATGTCGGATTCTGTTAGAGATTGGATGCAAGCTGAT
GTTATAATACAGATTGGAAGTCGGATAACGGGAAGGCGCATTTCTCAGATGATAGAGCAATGCACTCCGTGTGCGTACATCATGGT
CGACGATCATCCAGGTCGTCATGATCCTTCAAATATCGTGACACATAGGATACAAAGTACCATCAGTGAATTCAGTGATTGCTTGA
TTAAATGTTCCATCTCTCACGTAAGCACGAAAGGGTCGGAATTTATACGAGGACTGGATATGATGGCTGCCTGGGAAACATCTTTT
TTGATTAATTCCGAACAATCGTTGACTGAACCTTATGTCGCGCGAAAAATCTTTGAGACGATTCGCTGTGGATCCGCTTTGTTTTA
TGGAAATAGCATGCCAATACGTGATGCGGACATGTATGGGAGTAACAGGGTGCAGTGCACCCACAGTGCTTCTCTGATGTTGAGTT
CTGGTTTACCGTGTCATCCTGTGCATGTTACCGGAAATAGAGGTGCTAGTGGTATTGATGGTTTGATTAGCACAGCTATTGGGTTT
GCAGTTGGCTGCAATAAAAGAGTGCTTCTTGTAATCGGAGATATTTCATCTCTGCATGATACCAATGGGTTGGCACTACTTAAACA
ACGGACCTTCCGGAAACCGATGGTCATACTTGTGCTTAACAATCATGGTGGTGCTATCTTTAGTCAACTACCTGTTGCAAATACGA
CAGACAGAAGTATACTCGACCAATTTTTCTACACGTCTCACAATGTTTCAATACAAAACCTATGTCTGGCACATGGTGTGAAGCAT
GTACAAGTGCGAACAAAAAAGGAGTTGCAAGACGCATTGTTCACATCTCAAAGGGAAGACGTTGATTGTGTAGTGGAAGTTGAGAG
TGAAATTGACACCAATGTTGCTATTCATAGTAACTTGAGAAATTTTACTCGCAAAGCCTCGGATCATGCTTTCAACATCCTCTCGA
AGCTGTCAGTTGCAGATTCCAACTCGCAACATTACAAAATAAATAAAATGGATTACTCTATGTACCGGGTTCAGCTAAATGCTCCA
CCTACATCAGCTCTTACGGACTCCAAGACTAGCACATCCTACAGAGAAGGTTTTGTTATAAGTCTGTCTCTTGAAGATGGTAGTAT
TGGGTTTGGCGAGGTCGCCCCTCTTGAAATCCACAATGAAAATTTGCTTGATGTGGAAGAGCAACTTCGGTTTCTTATTCATGCCA
TAGAAGGACAAACAATCAATAACGCCCTAGCTCTGTTGAATTGTTCATTTTCTTCTTGGATATGGAACAGTTTAGGAATTCCGCCA
GGTTCAGTCTTTCCCAGTGTCAGATGCGGATTAGAGACGGCTCTTCTCAGTGCACTTGCAAGTAGACAAAGTAGCACTTTATTAAA
TATACTTAATCCCGCAAGCGAAAAATCGTCCAAGGAATCATCTGCCATTCAAATTTGCGCCCTAATTGACTCCTATGGAAGTCCAA
TGGACACAGCTTTCGTAGCATCCAATCTTGTTGCCGAAGGATTTAGAGCAATAAAATTAAAGGTTGCGCGTCGAGCTGATACCAAT

GAAGATATTGCTACTATACAAGAGGTGAGAAGGAAAGTGGGAAAAGATATTGTTCTCCGTGCAGATGCAAATAGGAAATGGAATTA
TGATGAAGCTGTCAAGTTTGCTCACTCGGTCAAAGATTGTGGCTTGCAATATATCGAGGAGCCTGTTAATAACGAGTATGATATAG
TGAAATTTTGCCAAGAAACTGGCCTACCAGTGGCATTGGATGAAACAATCAACTCGATCGGGGAGAATCCTCTTGAGGTCCTTCAG
AAATACAGCCACTCAGGAATAACTGCTGTTGTAATCAAACCAAGTGTTATCGGAGGTTTCGAAAGAGCAGCATTGATTGCAAGATG
GGCCCAACAGCACGGAAAGACGGTTGTAATTAGTGCTGCATTTGAAAGTTCACTCGGTTTGTCGGCCTATATCCAATTTGCTCATT
ACCTCGACCTGCAAAACGCCGAGATACAGAATTTGATGAATAAAGAACCTGCACCAGTCACAGCACACGGTTTCGGAACTTACAAA
TGGTTTAAGGAAGACGTGACTGCAGAGAATCTAAATATCCGTTATGATTCAGACTGTGATTTTGTTAAGGCTGATGCTGTTGATGC
CGGTCGATTTCTCCAGAATTGTCGGTTAAATCCTGATAAGGTTGTTAGAGTTTTTAATCAAGAAAAAGTGCGGGAATATCAGTTGG
CAGTTGATACAGAGGGCGTCTCATTTACTACAAATGTGCTAGAGATGGGAGAAAGCATTGATGGTACTGCAGTTGTGTTTCTTCAT
GGATTTCTTGGAACTGGAGAAGATTGGATCCCGACCATGAAAGCCATCTCAAGCTCAACCAGATGCCTTGCAATAGATCTTCCTGG
TCATGGTGGATCAAAGTTGCAATATAAGGGTACCAAGGGTTCAGATCAATCCGATTTATCGATTGATGTGGTGGTTGATATCTTAT
GCAAGGTGTTGAATAATATTACTACTCAGAAGGTTATACTCGTCGGGTACTCGATGGGAGCTAGGATATCTTTATACACAACACTA
AAACGCAGTGATAAGGTTGAAAGAGCAGTGATTATATCTGGAAGCCCGGGTTTAGTTGATAACGGTGCAAGAGAGATCCGTAGAGC
TAAAGATGACTTCAGAGCTAGCACACTCATGTCAAACGGTTTAGAATTTTTTACGGAGGCTTGGTATGCTGAAGAACTCTGGGCTA
GCTTAAAAACCCATCCACACTTCAAACAAATAGTTGCCAGTCGTTTGCAGCATGACGACTTGCATACTCTTGGCAAAGTTCTGTCT
GACTTAAGCATCGGAAGGCAGCCATCACTGTGGGAAGATCTGAAGCACTGCAAGGTGCCCCTCCAGATTATAGTGGGAGAAAAGGA
TGTCAAGTTCAAGAAAATCGCTCATGAGATGTATACAAAGATTGAACACGGAAACGGAAGTAGTAATTCACTGCCAGTTGCTGAAA
TTTTGAATGCTGGACATGCTGTTCATCTTGAGAATCCTCTTGGTCTTATTACTGCTTTAAGGCAGTTCATAAAGAGAGAAAAGAAT
ACCTAGTTACTTTTGTTTTTGTTTTTTTATTTTTTAAAGAAAAAAATAATACACAAGGAGTTGCATTGTTGTCCACAGACAGAAAA
ATAAAGGTGTGTTCATTTTTAGGACGAAAACAGTTCTGTTAACTGAATAGAGATTGGATTTGATTTATTCATTTGTTATGAATATT
AAAGGAGGCTTATGTAATTTTGAAATTTCTCTGGTTTTCAGGGTACTTTTAGAAGAAAAAAAAAAAAAAAAA
>ShPHYLLO Striga hermonthica Sh.c19093_g2_i7.6567
CCGAAACCCCAGCAACTGTCATAGTTCTTCTCTGGGACTTAGCGCCCTCCTACTCTTTGAACCTCCATTTAATTTATCAACAAAAT
GAGCTCCTTCACCTTATTCTACTCTCAATCTACGCTCCCTTTTCCACTTTCTCCACTCCAACCCAAACAGAAGACCATAATCACAG
TAACAAAGCGCGCTTTTCTAATGAAACCTGCTTTGCGATGTCTAACTCGCTTTAGCCACCACCAAAATCCCATTTCCCAGGTTTTG
AGCAGTTCATCGGAAAAGAATGAAGTCTTGGATGCTAAAGATGCGGCATTGCTGGTCAATACTTGCATTACGCGTAATTTGCCGCC
GGTTTTGAGTTTGGAACAAGGGCTGGAGAGGATTAAGGGGTCCGTGGAGGAATTGAGGGTTAATCCTCCTTGTTGCTCAAGTGGGA
TGTACAGATTTCAGCTGGCAGTGCCACCAAGTGCAAAAGCATTGAACTGGTTTTGCTCCCAACCGGATAAGTCAAACATTTTTCCT
CTATTTTTCCTTTCCAATGAGGAGAATCCGACATATAATTCGCTCTCTCTTGGAAGAACCAGGGGTGTTTTCGGTATTGGTTCAGC
CGTTGTCTTCAAGGATTGTTCTCCTCATAGTGCAGGAAAAGGCAGTGATATTAGAAGACATCTTTTGGCTGAACCAACAAGTGCAA
AGGTTTATGGTTTCTTGGATATTGAGTTCGTCACCGACATGTCTGCTATAAAGCATCAGAGTGGTTCGTATTACCTTTTCATCCCT
CAGATTGAGTTGGATGAGTTTGAAGATATCCCTTCCTTGACCGCGACATTGGCATGGGACGACTCTTCAATTTGTACCTTTAGTGA
AGCTGTTCGAAGATTTGAGCTTGCATTAGACCAGGCCATGCATACCTGTGCAAATAGAAGCCAACTTATTCGGTCTTCTCTTTCAA
AGTATCGCAATGCAGAGAAACATAAGGCAATGGTACGTGCAAATGCTTTGTTATTGGATGGGAAGCACACAGCAGCCAGCGCCCTG
GGGCTGGGAGATTCTTTGTCTTGTTGCAGTCAGTTTGTTGCTAGGCTTTCATCAACTTTATCAATTGCAGATAACATGATAGATGA
AACAAAATCAGTCAACAATGTGATTCAAGATTTCCCCAACATCAATGCTTTGTGGGCGTACCTTATAGTTGAGGAATGCACTCGAC
TTGGTTTGACGTATTTTTGTGTGGCCCCTGGATCAAGGTCATCTCCCTTGACAATTGCTGCCGCCAGTCATCCTCTTACCACTTGT
GTTGCATGCATTGATGAAAGATCTCTTGCATTTCATGCGCTCGGTTATGCAAAAGGTTCAGGAAAACCGGCAGTTGTCATAACATC
ATCAGGCACAGCTGTCACGAATCTTTTTCCGGCTGTGGTTGAGGCTTGCCAGAGTTTTGTACCAATGCTGTTGCTAACGGCTGATC
GCCCTCCCGAGCTTGTAGATGTTGGGGCTAACCAAGCTATTGATCAGGTGAAGCATTATGGGTCGTTCGTGAGGCACTTTTTCAGT
CTCCCTCCTCCTACAGACGAAATATCGGCAAAATACGTACTAACTACAGTAGACTCAGCTGTTGTTAAATCAACATCATCCCCAGT
GGGCCCTGTACACATCAACTGCCCTTTCCGGGAGCCACTTGCAAGTACCTCAAAAACTTGGAGTCCCAAGTGTTTAAGTGGATTGG
ACATTTGGATTTCAAATGCCGAGCCATTAACCAGCCATTCTTTGACTTGTTATAGTAACGTGAGAGAGCAGATGGTTGAGGTTGTT
AGGGTGGTCCAAAGGGCTAATCATGGGATTTTGGTGTTGGGTTCTATTCAGAAGGAAGACGATATGTGGGCAGCTCTTTTGCTGGC
CAAGCACTTGTTGTGGCCGGTTGCTGTTGATATCCAGTCGGGTTTGAGGTTGAGGAAATACTTGTCGTCTTTTCTTGACAGCAAGG
ATATATTGTTCGTTGATCAGCTCGACCAGCTGTTGCTGTCAGAGTCTGTAAGGGAATGGATGCGGGCAGATGTAATAATACAGGTT
GGGAGTCGGATAACAGGGAGACGCATTGCCCAGATGATGGATCATTGTTCACCTTGCTCCTACATCATGGTCGATGATCATCCAGC
TCGTCATGATCCTTCTAATATCATCACACACAGGATCCAAAGTACCATCCCCGAGTTCACTGATTACTTGATCAAGTGTTGCAGCC
CTCGTGCGAGCAACAAATGGCGGGAGTTAATACGAGGATTGGACATGAGGGCTTCCTGGGAGACCTCATTTCTGATTAATTCGGAA
CAATCCTTGACCGAACCTTACGTAGCACGGAAAATCTTCGAGGCAATCCGCTGTGGGTCAGCTTTGTTTTACGGGAACAGCATGCC
TATACGTGATGCGGACATGTACGGTAGTGACTGGGTGCATTGCACCCACAGTGCTGCTCTCATGTTGAGCTCTGGCTTGCCATGTC
ACCCGGTGCATGTTAGCGGGAATAGAGGGGCTAGCGGTATTGATGGGTTGATAAGCACTGCTATTGGATTTGCTGTTGGCTGCAAT
AAAAGAGTACTTCTTGTGATTGGCGATATTTCATTTTTGCATGATACGAACGGGTTGGCATTACTAAGACAATGGACATACCGGAA
ACCAATGGTCATACTTGTGCTTAACAATCATGGTGGTGCTATCTTTAGTCAACTACCAGTTGCAAATACCACAGATAGAAGCATAC
TAGACCAGTTTTTCTACACGTCTCACAATGTTTCGATACGCAATCTATGTTTAGCACATGGTGTGAAGCATGTTCAAGTGCAAACA
AAAAGGGAGTTAGAAGATGCATTGTCCACATCTCAAAAAGAAGACATTGATTGTGTGGTGGAAGTCGAGAGTGAAATTGATATGAA
CGTTGCTATTCATAGTAAGTTGAGGAACTTTTCTCGGAAAGCCTCGGATAATGCTCTTAACATCCTCTCAAAGCTGTCAGTTGAAG
ATTCCCACTCGCAAGATTACAAGATCCATAAAATGGATTACTCTCTCTACCGGGTTGAGCTTAATGCCCCACCTACTTCAGCTTCA
ATGGAGTCGAAGGCTCCCACATCCTATAGGGAGGGTTTTGTCATAAGTCTAGCTCTTGAAGATGGTAGTACTGGTTTTGGTGAGGT
GGCACCTCTTGAAATCCACAAAGAGAACTTGCTTGATGTGGAAGAGCAACTTCGGTTTCTTGTTCACGCTATAGAAGGAAAGACAA
TCAGTGGCATACTACCTCTGTTGAGTTGTTCAATTTCTTCTTGGATATGGAAGAATTTAGGAATTCCGCCAGGTTCAATCTTTCCC
AGTGTTCGATGTGGATTAGAGACAGCTCTCCTGAGTGCTATTGCAAGTAGACAAAATAGCACTTTACTGGATATAATCAGCCCCAC
AACCGAAAAAACGTCCGAGAAATCATCCCCTGTTCAAATTTGTGCCCTCATTGACTCCTACGGGACTCCAATGGACACAGCTCTTG

TAGCATCCAAGCTGGTTGCTGAAGGATTTACGGCTATAAAAATAAAAGTTGCACGTCGAGCAGATCCTGATGAAGACGTAGTTACA
ATACAAGAGGTGAGAAGGAAAGTGGGAAAAGATATTGTACTCCGTGCGGATGCTAATAGGAAATGGAATTATGATGAAGCCGTTAA
GTTTGCTCTCTCGACCAAAGACTGCTGCCTGCAATATATCGAGGAACCAGTGAATGATGAGAATGATATAGTGAAATTCTGTGAAG
AAACTGGTGTGCCAGTGGCTTTGGATGAAACAATCAACTCTATAGGGGAAAACCCTCTGGAGGTCCTCGGGAAGTACAGCCACTCG
GGAATTACTTCTGTTGTAATCAAACCTAGTGTCATCGGCGGGTTCGAAAAGGCAGCTCTGGTAGCAAGGTGGGCCCATCAGCACGG
GAAAATGGTTGTAGTTAGTGCTGCATACGAGAGTTCACTTGGTTTGTCAGCCTTCATCCAGTTCGCCCGTTTCATCGACCTGCAAA
ATGCCGAAATTCGAAGCTTGACGAGCAAGGAGCCCGGACCAATCACAGTGCACGGATTGGGGACATACAAATGGTTCAAAGAGGAT
GTGACGTTGGAGCATTTAAATATTCATTATAGTCCTGAGCACAGATCAGTCGAGGTCGATGCTGTTGATGCCGGTCGATTTCTCCA
GGATTTGCGGGTAAATAATGACGTAGTTGTCAGAAAGTTTATTGGTGAACAAGTGCGCAAGTACCAAGTAGCGGTTGATACAGACG
GTTTCTCGTTTACTACAAATGTGGTCGAGGCTGGAGAAAGCATTGATGGTAGTGCTGTTGTGTTCCTCCATGGGTTTCTTGGAAGC
GGAGATGATTGGGTCCCGATCATGAAATCCCTCTCAACCTCCACTAGATGCATTGCAATCGATCTACCCGGCCACGCTGAATCAAA
GTTGCAGTTTAACGGTTCCGATTTGTCAATCGATATGGTAGTTCATATCTTGTGCAAGGTGTTAAATAATCTCACCCCTCATAAGG
TCACTCTTGTGGGCTACTCAATGGGAGCTCGGATATCTTTATACACAGCCCTAAAATGTTCTCACAAGGTTGAAAAGGCTGTTATA
ATCTCGGGAAGCCCGGGTTTGATAGACAAGGATGCAAGGGAAATCCGTAAGGCTAAAGATGACTTTCGAGCAAGCACACTGGTGTC
AAATGGGTTACAGTTTTTTACAGAGGCTTGGTATGCTGAAGAACTCTGGGCAAGCTTAAGAACCCATCCACACTTTAAAATGATAG
TTAACAGCCGTTTGCAGCATGATGACTTGCCGACTCTTGGTAAAGTTCTCTCTGGCTTAAGCATCGGAAGGCAACCATCTTTGTGG
GAGGATCTGAAGCATTGCAAGGTCCCACTGCATATTTTAGTGGGAGAAAAAGATGCCAAGTTTAGAAGAATCGCTCATGAAATGTA
CGCCAAACTCGGCCATGAAAATGGAAGTTCTAATTATTCACCGCCAGTGACTGAAATTCCGAATGCTGGACATGCAGTTCATCTCG
AGAATCCTCTTGCTCTGATTACCGCTCTACGACAGTTCATATAGAGAGGGAAGACTAGTTAGATCCCTTGATTTATGTACAATAAA
ATAAAATTAAAAAAAAAAAGAAAAGA

>TvPHYLLO Triphysaria versicolor Tv.c112198_g1_i1.18287

ATGATATTCATAGTTAAAATTTAGCTCACTCTACTCTTGAACCTCCATTAACAATGAGCCCTTTCACCTTACACTCTCAAATCACA
CTCCCTTTTGCACTATCTCCACTCCAACTCCACCAAAAAACCATTATCACAATAACTAAGCGCACCCATTTCCTCCTCAAACCTCC
TCCGCCCTGTTTAACTCGCTTTAGCTGCCGACAAAATCCCATCTCCAAGGTAGTTAGAAAATCATCAATGGGAAAAGATCAAATCT
TGGATGCTGCATTGCTGGTCAATACTTGCATAAAGCGTAATTTGGGGCCGGTTTTGAGTTTAGAGCAAGGATTGGACCGGATTAAG
GAGGCTGTGGAGGAGTTAAAGGCTAATCATCCTTCTTGTTCAAGTGGGATGTATAGATTTCAGCTTGCAGTGCCTCCGAGTGCGAA
AGCGTTGAACTGGTTTTGTTCTCAACCGGAATTATCAAACGTGTTTCCATTATTCTTTCTTTCTAATGAGGAGAACCCAATATATA
ACTCACTTTCTCTTGGAAGAACGAGGGGCGTTTTTGGTATTGGTTCAGCTGTCGTCTTCGAGGATTGTTCTTCTCATGGTTCGAAG
AAAACCAGTGCTATACGAACATCTCCAAAGGTGTATGGTTTCTTGAATATCGAGCTCGACACAAATATGTCCACTATAAAGCATCA
GAGTGGTTCGACTTACCTTTTCATTCCTCAGATTGAGTTGGATGAATTCGAAGATATCCCTTTCTTGGCTGCCACGTTGGCATGGG
ACGACTCATCGATATGTACTTTTAGTGAAGCTGTTCAAAGATTTGAGCTCGCTTTTGACCAGGCCAGATACACATGTGGAAACGGT
AATCAATTGATACGATCTTCTCTTTTAAAGTTCAGCAATGCGGAGAAACATGGTGAAATGGTACGTGCAAATTCTCTGTTATTCGA
TGGGAAGCATATTGCAGCCAGCACCTTGGAATTGGGAGATGCTTCGTCTTGTTCTCGTCAGTTCGTTGCTAGGCTTTCGTCAACTT
TATCAATAGCAAACAACATGCACCCGAGTGACGAAACCAAATTAGTCAGCAATATGACTCAAGATTTTCCAAATATCAATGCTTTA
TGGGCATACCTTATAGTTGAAGAATGCACTCGACTCGGTTTGACATATTTTTGTGTAGCGCCTGGATCAAGATCATCTCCTTTAAC
TATAGCTGCAGCTAGTCACCCTCTTACGACTTGTATCGCATGTATCGACGAACGATCACTCGCGTTTCATGCTCTCGGTTATGCCA
AAGGCTCCCAAAAGCCAGCTATCATTATAACATCTTCAGGCACAGCCGTCTCAAATCTTTTTCCCGCCGTTGTAGAGGCTAGCCAG
AGTTTTGTACCAATGCTATTGCTAACTGCCGATCGTCCTCCCGAGCTCGTAGATGTTGGGTCAAACCAAGCAATCAATCAGATAAA
TCATTACGGATCATTCGTGAGGCAATTTTTCAGTCTTCCCTCTCCTTCTGACGATATATCCGCAAGATATATTCTTACCACTATCG
ACTCGGCTGTATTTAAAGCAACTTCTTCGCCAACCGGTCCAATACACATAAACTGCCCTTTTAAAGAGCCACTAGCAAACAGTCTA
AAAAATTGGAACCGTAACTGTTTAAACGGACTAGACGTTTGGATTTCGAACGCCAAACCGTTTACGAGCTACATTCCGTTAAAAAA
TGCATTGACGTTTGATATGATTGAAGTGACGAGGCTGGTCCAAGGGGCCGATCGAGGGATTTTAGTGTTGGGTTCGATTCAGAAAG
AGGATGATATGTGGGCTGCTCTTTTATTGGCTAAGCATTTGTTATGGCCAGTTGTTGTCGATATTCAGTCGGGTTTGCGGGTGAGA
AAGTACGTTTCGTCGGTTCTTGACAGCAAGGATATATTGTTCGTTGATCATCTCGATCAGCTGTTATTGTCGGATTCTGTCAAGAA
TTGGATGCGGGCCGATGTTGTAATACAGATTGGGAGTCGGATAACCGGGAGACGAATTTCTCAAATGATAGAGAAATGCACTCCGT
GTCCGTACATCTTAGTCGACGATCATCCGGGCCGTCACGATCCTTCTCATATCATCACACACAGGATACAAAGCACCATCTCCGAG
TTCAGCTTTTGCTTGATCAAATCTTACACACCCGATATAAGCAAGAAATGGACGGATCTTATTCGAGGATTGGACACAATGGTTGC
CTGGGAAACGTCATTTTTGATTAATTCCGAGCAATCATTGACCGAACCTTACGTAGCGCGAAAAATCTCCGAGATGATCCGATGTG
GGTCCGCTTTATTTTATGGGAATAGCATGCCGATACGTGACGGGGACATGTACGCGAGTAACTTGGTGCAATGCACCCATAGCGAT
TCTCTAATGTTGAACTCCGGTTTAGAGTGTCACCCGGTGCATGTTAGTGGAAATAGAGGAGCTAGTGGTATAGATGGTTTGATTAG
CACTGCTATTGGATTTGCTGTCGGCTGTAACAAGAGAGTGCTTCTTGTGATCGGAGATATTTCGTTTCTGCATGATACTAACGGGC
TGGCATTACTGAGACAACGGACATCTCGGAAACCGATGGTCATACTTGTGCTTAACAATCACGGTGGGGCCATATTTAGTCAACTG
CCGGTTGCAAGTACGATAGACAGAAGTATACTCGATCAGTTTTTCTACACGTCTCACAATGTTTCGATACGCGATCTATGTCTCGC
ACATGGTGTGAAGCATATATCAGTTCAAACTAAAAGTGAATTGCAAGACACATTGTTCAAATCTCAAAGAGAAGAAGTTGATTGTG
TAGTGGAAGTCGATAGCGAAATTGATACGAACGTTGCTATTCATAGTACTTTGAGGGATTTTAATCGCAAGGCCTCGGATCAAGCT
TTAAACATCCTCTCAAATCTTTCAGTTTCAGTTGAAGATGACAACTTTCAAGATTACAAGATTAATAAAATGGATTATTCTCTGTA
CCGGGTTCAGCTTAATGCTCCACCTACTTCAGCTTCTTCGACGAATTCCAAGAATAGCGCATCTTATAGAGAAGGCTTTGTTATAA
CTCTGTCTCTTGAAGATGGTAGTATCGGATTTGGCGAGATTGCACCTCTTGAGATTCACAAAGAAAACTTACTTGATGTCGAAGAG
CAACTTCGTTTTCTTGTTCACGCCATACAAGGAAAGACGATCAGTAACATCATACCTTTATTGAAATGCTCGTTTTCTTCTTGGAT
ATGGAACAATTTAGGAATTCCGCCAGGTTCGATTTTTCCAAGTGTACGGTGCGGATTAGAGATGGCTCTCCTCAGTGTAATTGCAA
GTAGACAAAATAGCACATTGTTGGATATAATTAATCCAACAAGCGAAGAATCGTCCCCCGTTCAAATTTGTGCTCTTATCGACTCT
TATGGGAGTCCAACGGAAACAGCTTTTGTTGCCTCCAACCTTGTTGCCGAAGGATTTACGGCTATAAAAATAAAAGTAGCGCGCCG
AGCAAATCCCGATGAAGATATCGCTACAATACAAGAGGTGAGAAGGAAAGTGGGACCAGATATTGTACTCCGTGTCGATGCAAATA

GAAAATGGACATATGAGGAAGCTGTTAAGTTTGCTCGATCCGTCAAAGATTGCTGCTTGCAGTATATTGAGGAGCCTGTTAACAAC
AAGGATGATATAGTGCGATTCTGTAAAGAAACGGGCTTACCAGTTGCATTGGATGAAACAATAAATTCGATCGGAGATAGTATTTC
TCTTGAGGTCCTTCAGAAGTACAGCCACTCTGGAGTAGCTGCTGTTGTAATCAAACCAAGTGTAATTGGAGGTTTCGAAAAGGCGG
CATATGTTGCGAGATGGGCCCACCAGAACGGAAAAACAGCTGTTATTAGTGCTGCATTCGAAAGCTCGCTCGGTTTATCAGCCTAT
ATCCAATTCGCTCGTTACCTCGACCTGCAAAATGCCGAGATACATAGTCTGATGAACAAGGAGCCTGCACCAATCACAGCTCACGG
TTTCGGAACTTACAAGTGGTTTAAAGAAAACGTAACGGATGAGAATCTGAATATCCGTTATGATCCGGACCGTCGGTTTGTCGTTG
CTGATGCTAATGATGCCGGTCGATTTCTCCAGAATTGTCGGATAAATCGAGACTCGATTGTTCGGAGCTTCGTTCATGAAGAATTA
AAGGAATATCAGTTGACAGTTGATACGGATTTTGTCTCGTTTACTACAAATGTGGTCGAGATCGGAGAAAGCACCGATGGTACTAC
AGTTGTGTTTCTTCATGGATTTCTCGGAAACGGCAAAGATTGGGTTCCGATTATGAAGGCAATCTCAAGCTCAACTAGATGCATTG
CAATTGATATTCCTGGTCACGGCGGATCAAAACTGCAATATAAGGGTATCAACGGTTCAGATCAACCCGATTTATCTATTGACTTA
ATAGTTGATATATTATGTAAGCTGTTGGATAATATTACTCCTCGGAAAGTAATACTCGTCGGTTATTCAATGGGAGCCAGAATCTC
ATTATACACAGCCCTGAAACACAGTAATAAGGTTGAAAGTGCTGTGATAATATCTGGAAGTCCGGGTTTGGTAGATAACGAAGATA
GAGCAATCCGAAAGGCTAAAGATGACTTTAGGGCTAGCACGCTCGTCTCGAACGGGTTAGAATTTTTTACGGAAGCTTGGTATGCT
GAACAAATGTGGGCTAGCTTAAGAAGCCATCCACATTTCAAGCAGATAGTTACAAGTCGTTTGCAGCAAGGCGACTTGCAAATGCT
CGGAAAAGTTCTGTCTGAATTGAGCATCGGAAGGCAACCGTCGTTGTGGGAAGATTTAAAGCATTGTGAAGTGCCGCTTCAGATAA
TCGTGGGCGAAAAGGATGACAAGTTCAAGAAAATTGCTCGTGAGATGTACGAAAAAATCGGCTTTGAAAATGGAAATAAAAGCTCG
CCTCCGGTTGCTGAAATTCCAAATGCTGGACATGCTGTCCATCTTGAGAATCCCCTTGCTCTTATCACTGTTTTAAGGCAGTTTAT
AAAAAGAGAAAATAGTACTTAATAAAAAAAATTAAAACAAAAAAAAAATTATGTTCAGTTTTTCAAAATAAAATAATACAGATTAC
AGAAATATATTCATATTTCTTGTAATAAAGATTGAACAAGTCCTTTGCTTCAAAAACAGAGAATAAAAAGATATTGAATAAGATGT
GTTTTCATTTTGTTTGGCATGACTTAT

>PaMenE Phelipanche aegyptiaca Pa.c23477_g1_i1.14490
TCTCTCTCTCTCTCTCTCTCTCTTTTGCTATATACTTCTGTAAGTTTCCCCTTCTGCCCTCCGCCGTAATGGCTAATTACTCGGAG
TCCCACATCTGCCAGTGCTTGAGCCGCCTCGCCGCCGTCAGCCGCATCTCCACCGTCACCATATATGGAGACCGCCGGAAAACCGG
AATGCAATTTGTCGAAGAAGTTATGGGCCTGGCACATGGACTTCTCCAACTCGGTATCAAGCCCGGTGACGTTGTCTCCATTTCTG
CTCTCAACAGTGATTTGTATTTGGAATGGATGCTTGCTATTACTTATGTTGGAGGAATTGCTGCTCCACTAAATTATCGATGGAGC
TTGGAAGAGGCTAAGTCAGCATTGGAGGTAGCAAGACCCGTATTATTAGTAACCGACTCAAGCCCGGGCTATTGGCATTCCAAATT
TCAGATTGATTATGTCCCGTCTCTGAGGTGGCATGTCTTGATGGATATGCCTGTCAAAGCCGACAGTACCAGGACAATTTTTGCGG
CCGAATTTCTTAAGGAGCCTGCCGGAAGATCTGTAAAAGTGGACTATCTTTGGGCACCTGAAAGAGCTGCAATTATATGCTTCACC
TCAGGAACCACTGGAAGGCCTAAGGGAGCTACTATAAGTCACTCGGCTTTAATTGTGCAATCCCTTGCAAAAATTGCAATTGTTCG
CTATAATGAGGATGACGTATATCTTCACACTGCTCCCCTGTGCCATATAGGCGGAATATCATCAGCCTTGGCCATGCTAATGGCAG
GAGGTTGTCATGTTATATTACCAAAGTTTGAGGCTAGCTTAGCAATTGAAGCCATTAGGGAACACAGCGTCACTTCCTTGATCACT
GTACCCACCATGATGGCTGATCTAATCTCTTCTCATAGAATGGATCAAACATCTACAAGTTTCGAATCTGTGAAGAAGATCCTGAA
TGGAGGTGGTGGTCTATCAGTTGAGCTCATAAAAGATGCAACCAAATTATTTCCACTAGCCACGCTTCTCTCAGCTTATGGGATGA
CAGAGGCATGTTCTTCTCTTACCTTCATGACTCTTTACGAGCCGACAAAAGAAGGCCATCACTTGCAGCCACATGATATACAAAAG
TCCAACTTAATTAGTTGTCAAGGAGGTATATGTGTAGGAAAACCTGCTCCACATGTTGAACTACAAGTAAATGCCGAGGAATCTTG
TAATACCGGGAGAATATTGATGAGGGGTCCCCACGTAATGCTTCGTTACTGGGGTCAAAGTCCATCAAAACATTTGAGTCCTGTTT
ATGAAGGTTGGCTTGATACTGGGGATATAGGGCAGGTAGATAATCATGGTAATTTGTGGCTTATTGGACGAGCAAAGGATAGAATC
AAGAGTGGAGGGGAAAACATTTATCCAGAAGAGGTAGAGGCTGTCTTATCCCAACATCTTGGAATTTCTAGGATTGTTGTTGTTGG
AATTCCAGATTCTCGGTTGACGGAGATGGTATTTGCCTGTATTAAGCTAAACGACGGTTGGAGATGGACTGATTTTGTTGGCAATC
ACTCAGCAGGAGAACATATACAGTGTTTGTCCAGTGAGATACTCAAACGCTTTTGTAGAGAAAAAAATTTAACAGGGTTTAAAATT
CCCAAAAGATTTGTTTTGTGGAAGAACGATTTTCCAATAACAACAACTGGAAAATTAAGGAGAGACCAAGTCAGAGCAGAAGTTAT
GTCTCATACTCAGTTCCTCACCAGCAAACTTTAATTGCCACAAAATATTTTCTGGGAAATAATGATTGTACAGAGTTTGTGCACAT
TTTCTTTAAATTGATCAAATCTGGACAACAAAAGGGTGCGTGTTCATTTTGAATTTTGATAAGAATAAAAGTTGCTATGTATTTAA
AATGGGGTTGGTTGAGTTATTGAATAATCTAATAATTAATACCTCATCCGTTTCAGGAATATTGCAAAAAAAAAAAAAAA
>ShMenE Striga hermonthica Sh.c16227_g1_i2.0
CGGGGTCGCAGGCACTGAAAAAAAAACCAGTGTACAAACGCCTAGGCCCTGCGTATTAGGGTGCTCGCTTAGCGCCTAGCGCCTAAT
TTTCCATTGGCCACGTCGCCTCACTTCGAAACGACGTCGTATATCAGGCAAAGCGTTTTCGTCTCGGATAGAGCAAACGGTGTTGA
TATTACTCATTCGCAATTCTACAAAGACGATGTCATCTCCCTTTTTGTGTCGGTCGATTCCTCTTGAAATTTCAATCCTGTGTGCA
TATTTCCTTTGCAAAAGATCTGTTTATTTTTTCTTTTCTTTCCCATTTGGGTCTGATTTTTCCATGGTCGTCTAGGGCGAGCTCTT
TGGTGTGAACGGCGAGCGCAATTTCCTTCAGCTCTCGAGCAGGCCAGAAAGATTGAAGCACGACGACCTCGAGGCCATGGATTGGA
GGGGAACCGATTTCAATTTCATCATATCCGATTCTGGGCTCTGATGGTTCAGATCCATTTCTGCGGAATTTCGCTCATGGTATCGA
AGGAGCTGGCGGCCGCGTGAGGTTTTTGCGAGAGGAAAAATCTCATAATCGTTGGAATCACGCTTCTTTTTAAACGGAATCTTAAG
CCTCTTCGCCAGAATCTTGATCCTATATAATTGGTTCGTTGAATCAACGAGTTTTGGAGCATTTGTCGACCTTGCTGAAGTATTCT
GATGCTTAAAACCTGTTCGGCCCCTGATCACTTTGTACTGGCTATCAAAGTTGTGTGTGCCCGTAGTAGGGGAATCGAAGATATCA
ACCATCGACGGCCTCTGCTCAGCTCAAAACGCACAATTCAGCCGTTTCTGGCCCAGAGAATAAGCCTTTACCGTTGCCGGCGAATT
CGGGATTTTGGTTGGCATAACACATGTACTTACAGCAGAGCTGGAACCTCCTGGTGATCAGCGGCGGATTGAATGATGCTGCTCGA
TTGCATTTGATTTTCGTTGGACAAAAAAGGGAATTGCTAAAGTTGAAGAAACTTATAGTTCTCACAAAAGAAGGAAAATGTATGAC
CTTGACTATGTTTTCTCCTCAGAGTGACTTGTATTTGGAATGGATGCTTGCTATCACTTATGTTGGAGGAATTGCAGCTCCACTGA
ACTATTGATGGAGTTTGGAAGAGGCTCGTTCGGCGATGGAGTTAGCAAGAACCGTTTTGTTAGTAACCGATTCAAGCCTGGTTATT
GGCATTTTATGTTCCGTTTTTACGATGGCACGTTTTGATGGATACGAAAATTGAAGCTGAGAGTAACAGGACAATTTTCGCATCTG
AATTAGTTAAGGAGATTGGTGGAGGATTTGCTGATGTAGACTATAGATGGGCACCTGAAAGAGCCGCGATTATATGCTTCACGTCA
GGGACAACTGGGAAGCTGAAAGGAGCTACAATAAGTCACTCGGCTCTCGTTGTGCAATCCCTTGCGGAAGTTGCAATTGTTCGTTA

TGACGAAAATGATGTATATCTTCACACAGCTCCACTGTGCCACATTGGCGGAATATCATCAGCAATGGCCGTTTAGATGGCCGGGG
GTTCTCATGTTATATTACCAAAGTTCGAGGCTAATTTAGCAATTGAAGTCATTAAGAAACACAATGTGACGCCCTTGATCACTGTA
CCGGCACTGATGGCCGATTTAATCTCTTTTAATCGATTAAACAGAACATCAGTGACTTACGAGTCGGTTAAAAAGATTCTGAATGG
AGGTGGTGGCCTGTCGACCGAGCTCACTAAAGACACAATCGAAATTTTTCTGAGTGCCACACTTATGTCAGCTTATGGGATGACAG
AGGCATGTTCGTCTCTTACCTTCATGACTCTTTTCGACCCAACCAAAACGACCCATCAGAAATACCACTCGAGTTTCCAAGGAGGC
ATTTGTGTAGGACAATCGGCTACACATGTCAAACTAAAAATAAGCCCGGACGAAAAATCTTTAAACACCGGGAAAATACTAACGAG
GGGTCCACACGTAATGCTCGGTTACTGGGGTCAAAGGACATCCAAATCCAATCATTCGAAACCAGGCCTCGAAGGCTGGCTCGATA
CTGGAGACATAGGGCAGATAGACGATCACGGTAATCTATGGCTTGCCGGACGAGCAAAGGATAGAATCAAGAGTGGCGGGGAAAAT
GTTCATCCTGAAGAGGTTGAGTGTGTCCTATCCCAACATCCTGGGATTTCGAGGATTGTTGTTATTGGGATTCCAGATGCTCGGTT
GACAGAGATTGTAACCGCATGTGTTCAACTAAAAAGCGACTGGCAATGGGTTGATTTTGGCGCCGGTCGGTCAGCAGGAGTCGAAC
AAGTCAAGTGTTTGTCGAGTGAGATCCTTAGGCAATTTTGCAGACAAAAGAACTTAACAAGATTTAAAATTCCAAAAAGGTTTATT
CTGTGGGAGAACGATTTTCCAACGACGACGACTGGGAAGTTAAGGAGAGACCAATTAATGGTAGAAGTTATGTCTCATACTCAGTT
TGTGTCCAGCAAACTATAGACAGTTCACACATAATCACAAATATGTGAAAATTAGTGTATAGAGTTTGTAGCTTAATGTGTGGCCA
TTTCTTGTAAGATAATAATAAGTACAGGGAGATTAATGTGAAATTTGGTGATCAAATTGAAGGATGTGAATGTTGAGAACTGATGT
CCTGAAGCATTTGTGTAATAAAATTAATCCTTTGGGGTTTGTGTAATAAAATTCTGCATTTTTTTTTTTAATATATAGAAGGTTGA
GTATGTTTTGATTCTTACACAAATTTTGTGTGGGTCAGCCTAACCAGCAGACAAATAAATTG
>TvMenE Triphysaria versicolor Tv.c6492_g1_i1.12968
ATTTGAAACTCAGTTAGACCCCAAAGCCTACTCAAATGATTTTGCCAATTTTAAAACGATTTTATTATTTTAATGGGAAAAGAAAA
CACTATAAAATGGTTCAATAGCATATCATCAACTCTCTGCCTCTCCCTCCATATCTTCCACTATCCCTCTCTAAACTCCTAATATC
TTCGTACATCTCTGAATTTCCCAATTTGCCCTCCGCCGTAATGGCAAATTACTCGGAGCCCCACATCTGCCAGTGCTTGAGCCGCC
TCGCCGCAGTTAGCCGCGATCCAACCGTCACCATTTGCGGCGATCGCCGGAAAACAGGAACGCGATTCGTCGGAGGAGTTATGGGC
CTGGCCCATGGGCTTCTCCAACTCGGAATCAAGCCCGGTGACGTTGTCTCCATTTCTGCTCTCAACAGTGATTTGTATTTGGAATG
GATGCTTGCTATTACTTATGTTGGAGGAATTGCTGCCCCTCTCAACTACAGATGGAGCTTGGAAGAGGCTAAATCGGCAATGGAGT
TAGTCCGGCCCGTATTATTAGTAACCGACTCGAGCCGTGGTTACTGGCATTCGAAGTTTCAAATCGACTCTGTTCCGTTTCTAAGG
TGGCATGCCTTGATGGACATGCCTCTTAAATCCCATAGTAGCGGGACAACTGAATTGCTGAAGGAGCCTGCTGTACTAGACTATCT
TTGGGCACCTGAAAGAGCTGCAATTATATGCTTCACCTCAGGGACCACCGGAAGGCCAAAGGGAGCTACCATAAGCCACTCAGCCT
TAATCGTTCAGTCCCTAGCAAAACTTGCAATCGTTCGCTATGACGAGGATGATGTGTATCTTCACACAGCTCCATTGTGCCACATA
GGCGGGATATCGTCAGCGCTAGCCATGCTAATGGCAGGGGGTTGTCATGTTATATTACCAAAGTTTGAGGCCAATTTAGCAATTAA
AGCCATTAGGGAACACAATGTGACGTCTTTGATTACTGTACCCACGATGATGGCTGATCTAATCTCAATTAATAGGATGAATCAAA
CATTTGAGACTTTCGAGTCTGTAAAGAAGGTTCTGAATGGAGGTGGTGGTCTTTCAGTTGATCTCATAAAAAATGCAACCGAAATT
TTCCCAAGAGCCACACTTATCTCAGCTTATGGAATGACAGAGGCATGTTCCTCGCTAACCTTCATGACTCTTTACGATCCAACAAA
GGAGAATCCACATAAACAGTCGTCGTCATATTACAATGATGTCCGAAAATCCAACTTAAGTCGTGAAGGAGGTACTTGTGTAGGAA
AACCTGCTCCTCATGTTGAGCTAAAAGTATCCGGCGATGGGTCTCCTAATAATACCGGGAGAATATTAATGAGGGGTCCACATGTA
ATGCTCCATTACTGGGGTCAAAGTCCATCGGATCATTTGGATCCTGTTTATGGAAGTTGGCTCGATACCGGAGATATAGGACAGTT
AGATGGTCACGGTAATTTGTGGCTTGTCGGACGAGCAAAAGATAGAATCAAGAGCGGAGGGGAGAACATTTATCCAGAAGAGGTAG
AGGCTGTCTTGGCCCAACATTCTGGAATTTCCAAAATTGTGGTTGTTGGAATACCGGATTCTCGGTTGACGGAGATGTTAATAGCC
TGTGTTAGACTAAAAGACGGTTGGCGATGGGCTGAGTTTGGTGCCAATCGTTCGGCAGGAGACCAAATCCAGTGTTTGTCCAGTGA
GATGCTTAGACACTTTTGTAGAGAGAAAAACTTAACAGGGTTTAAGATTCCAAGGAGATTTGCTTTGTGGACGAATGATTTTCCGA
TGACAACAACTGGGAAATTAAAGAGAGATCGAGTCAGAGAAGAAGTTGTTATGTCTCATACTCGCTTTCTACCCAGCAAACTATAA
TTCCCTCGATATATTTTATCAGGGATGAAACACATTCAAATGTAATGATGTGATTTGTATTTTCAGTATCCCCTACTGTGATTAGC
AAGAATATAAGCCTGAGTGGCTTTGGAGACTTGAAGAGGCAAAAATGTAATTAGGGCTATACTTAAAAGAATTTGTGTTCACTATT
CATATCATTTTTAGTATTAGATTGTGTTGTAAAAAAAAAAAAAAAAAAAAAAACCAAAAAAAAAAAAAAAAAAAAAAAC

>PaMenB Phelipanche aegyptiaca Pa.c21895_g1_i1.5023
CCGAAACCCCGTGACCCAAGAAAACTACTTCCCCAAACTACAAAACAACATTATAAATACTTCTTCCCATTATTTCCATCTCCACT
AATTTCAATACTCCCCTTAAAGTCAAAACATAATTGCCTTTTACAACTGATTATCACTCTAAAACAATGGCGGTCACTATGACAGG
AAAAGATGCTGAGATAATCAACAGAAGAATGGCCTCAGTTGCCAGACATCTCATCCCAGCTCAAAACCCAGACACAAATAATAATA
CTTACAATTTCATTTCCGGATCGAATTGTAGCTCCAAATTTAACGACACGTATCACCGGGTCCAGGGAGAAGTCCCGACGCATAAC
CCGGAATGGAAACCCGCCCTGGATGAGTCCGGCAAGGAGTTTACGGACATTATCTATGAAAAAGCTGTCGACGAGGGCATCGCTAA
GATAATGATTAATAGGCCGGAGAGAAGAAATGCCTTCCGGCCACAAACGGTGAAGGAGCTGATGCGGGCATTTAACGACGCCAGGG
ATGATAACTCGATCGGAGTTATTATTTTCACTGGGAAGGGCACCAAGGCTTTCTGCAGTGGAGGGGATCAATCTCTAAGAGGCAAG
GAAGGTTACGTCGATTACGATAATTTTGGTCGCCTTAACGTTTTAGATCTTCAGGTGCAAATTCGCCGCCTTCCGAAGCCGGTGAT
AGCAATGGTTGCGGGGTATGCAGTAGGGGGTGGACATGTGCTCCACATGGTGTGCGATTTAACAATTGCAGCTGATAATGCTATTT
TTGGTCAAACAGGACCTAAGGTTGGAAGCTTCGATGCTGGTTATGGGGCTTCAATAATGTCTCGTTTGGTTGGGCCCAAACGGGCA
CGCGAAATGTGGTACACGGCAAGGTTTTATAATGCAACGGAAGCGGAGAAAATGGGACTTGTCAACACTGTTGTTCCTCTGGAAAA
GCTTGAAGAGGAAACAATCAAATGGTGCAGAGAAATTCTGAGGAACAGTCCGATGGCTATTCGTCTGTGTAAATCAGCTATCAACG
CAGCCGATGATGGTCATGCCGGACTTCAGCAAATCGGAGGAGATGCAACGCTTCTATTTTACGGGTCAGAGGAAGGCACGGAAGGG
AAGAATGCATACTTGCAACGCAGAAAACCAGACTTCTCAAGATTTCCTAAGCTTCCATGATATTAATATATATGTGGAGTAGCAAA
GTATTTTTCATATGGTTATGCATGTTTGAGATGAGAAATAATGGGTTGGGTGAGAAAGAATAAAGTTGTGTGAAGATTTGATTTGA
CTACCTTTTTTTCTTTTACGTATCTTTTCTGTTGTATCACAAAATATTGGTTATCAATAATCTTTTACTTCGTTTACTTGATGTTC
AATTCCACGAGTTTCGTATAGTTTTATGTTCTGAACTTAATTAGCAATGGAATAAGGTTTTTCAATATTTCAAATCAAAGATATTG
TACATCCTTTTATATCACAAAAAAAAAAAAAA
>ShMenB Striga hermonthica Sh.c17026_g1_i1.3451

GAGTAATTTAATCAAACCATCAACTTCACAATTACAGCTCTTGTTAAACTAAGTCCTATAAATACAACCTTGAGTTATCCTCATTT
GTGCACCAAATTTCAGTTCTAGTTAATTTCGACAGTTCGATTAAATTACTCCGACGAGATGAACGGGAAAGACGCCGAGATAGTCA
ACCGGAGAATGGCGTCCGTCGCCAGACATTTAATTGGGCCTCAAGGCCCGAGCCCAAATAACGCCCTCATATCCGGGTCGGGGTGC
AGCTCCGGATTCAACGACACGTACCATCGGGTCCACGGGGACGTCCCGACCCACGACCCGACTTGGAAACCCGCTTTGGATGAGTC
GGGCAAGGAATTTACCGACATTATTTACGAGAAAGCCGTCGGGGAAGGCATCGCTAAGATAACGATAAATAGGCCAGAAAGGAGAA
ATGCTTTTCGGCCACAAACGGTGAAAGAGCTAATGCGCGCGTTCAACGACGCAAGAGATGATAACTCTATTGGGGTTATTATTTTC
ACTGGGAAGGGCACGCAAGCCTTTTGCAGTGGAGGCGATCAATCTCTTAGAGGCAAAGAAGGCTACGTAGATTATGATAATTTTGG
ACGTCTCAATGTTTTAGATCTCCAGGTGCAAATTCGACGCCTACCTAAGCCAGTGATAGCTATGGTTGCTGGGTATGCAGTTGGGG
GTGGACATGTGCTTCACATGATTTGTGACTTAACAATTGCAGCTGATAATGCAGTGTTTGGCCAAACAGGGCCTAAGGTTGGAAGC
TTTGATGCTGGTTATGGAGCTTCGATAATGTCCCGTTTGGTTGGGCCGAAGCGCGCACGCGAAATGTGGTACATGACGAGGTTTTA
CAATGCTGCCGAAGCAGAGAAAATGGGACTCGTCAACACCGTTGTTCCTCTCGAGAAGCTTGAAGAGGAAACAATCAAATGGTGCA
GAGAGATTCTGAGGAACAGCCCAATGGCTATTCGTCTTTGCAAATCGGCCATTAATGCGGTCGATGATGGCCATGCCGGACTTCAG
CAAATCGGGGGAGACGCTACGCTTCTTTTTTACGGGACAGAGGAAGGTACGGAGGGAAAGAATGCGTACTTGCAGCGTAGAAAACC
CGATTTCTCCAGATTTCCAAAGCTCCCATGATTTCATATTCATATGTGGAGTTCTTAATAATAATTGATGGAAATAAAAATAAAAA
AAATAAAAATAAAATGAATAAAGTTGTTTGTTGAGTGATGATTTTAAGTAACTTATACTAATTTCATCATTTTGTATCAGAAAGAA
ACAGATGCATGCTGCTTATTATCAATAAACTTGTAATTTTCTTATGTTTATTTCAGTGCTTCTGAGAATTTTGTGACATTCATAAA
TTTTTATGTTGGCAGTAGATTAATTATACGGGAAATGCTACAATCCTAGATCATCATATAACCATCGTGGATAGTTGATCTAACGG
TT
>TvMenB Triphysaria versicolor Tv.c8078_g2_i2.0
CTCCGGCCGACGGAGATATCCCTTCACACTCATCCATCACCTTCTCTTCACACTTCACACTCTAAACCCATTATAAATACAACGTC
CCATTAACCCAATTTACCCAATAACCCCACTCAAATTCAAGAAATTCATTTGCCTTAATCACCTGATTACGTCATCCAAAACCAAT
GGCTAAATTAACATTCGAAGATGCTGACATAATCAACCGGCGAATTGCCTCTATTTCCCGGCATCTCAGTCCACCTCAAAACCCGG
ACCCGAATAACCCAAATCTCATTTCCGGGTCAAACTGCAGCTCCAAATTTAACGACACTTTCCACCGGGTCAACGGAGAAGTTCCG
ACCCATATCCCGGAATGGAAACCCGCTTTGGATGAGTCCGGCAAGGAGTATACCGACATTTTATACGAGAAATCCGTCGGAGAAGG
CATCGCTAAGATTACGATTAATCGGCCGGAGAGGAGAAATGCTTTCCGGCCACAAACGGTGAAAGAGCTAATGCGTGCGTTTAATG
ATGCTAGAGATGATAATTCTATCGGAGTTATTATTTTTACCGGGAAGGGCACATTGGCCTTTTGCAGTGGAGGTGACCAATCACTA
AGAGGCAAAGAAGGTTATGTTGATTTTGATAATTTTGGTCGCCTTAATGTTTTAGATCTTCAGGTACAAATCCGTCGCCTTCCCAA
GCCCGTGATAGCAATGGTTGCAGGGTACGCAGTGGGGGGTGGCCATGTGCTTCACATGGTTTGTGATTTAACAATTGCAGCTGATA
ACGCAGTTTTTGGCCAAACAGGACCTAAGGTTGGAAGCTTCGATGCCGGTTATGGGGCTTCTATAATGACTCGTTTGGTTGGGCCG
AAACGAGCACGCGAAATGTGGTACACGACGAGGTTTTATAATGCAGCCGAAGCCGAGAAAATGGGACTAGTCAACACTGTTGTTCC
GCTTGAGAAGCTCGAAGAGGAAACAATCAAAATGTGTAGAGAGATTCTGAGGAACAGTCCGATGGCTATTCGTTTATGCAAATCAG
CGATTAATGCGGTCGATGATGGTCATGCGGGCCTTCAGCAAATCGGAGGAGATGCAACACTTCTATTTTACGGGACAGAGGAAGGG
ACGGAGGGGAAGAATGCGTACTTGGAACGTCGAAAACCGGACTTCTCGAGATTTCCTAAGCTTCCATAATTTAGAGTATGAGTTTA
GGAATAATGTTTGGAGAGAAAATAATAAAGTTGTGTGGTTTTTATTCTTATTTATTTTTATCATGTAGTGTCACAAACAATTGGAA
ACTATAATTTCCTTAATAAAAGATGTGTTATGATTCTTATGAATGAAATCAAGATTTAAAAGATGTTGAGCATATTGATATGCTAT
GAAACACGTTGTTA

>PaDHNAT Phelipanche aegyptiaca Pa.c23042_g1_i1.10619
TTTGCCTCAAGTTTTTATGCTATTAGCTCATAATTTTGACCCGGAGAAAAATTGGTGATCAATATTAGTTCTCTTTTCTGGTAGAG
TTGGTAAAAAAAAAATAGTAGATCACCGCAACAAATTTGATTCAAATTAGTCGAGAGAAGAGATGAACCAACCACCACCTTCCGCC
AGGCCACCGTCACCTCCGTCGAACACAAAGGAATTGGATTTTCCGCTCCACACACTCGGTTTTAAATTCGACTGCCTTTCGCCCGA
TAAGGTTTCGGGACACCTCCTTATCACTTCAGAGTGCTGTCAGCCGTTCAAGGTGTTGCATGGAGGGGTGTCGGCTCTTATAGCGG
AGTCTCTAGCAAGCATAGGAGCTCACCTTGCTTCTGGTCTGCAGAGAGTTGCCGGTGTACACCTTAGCATCAGTCACCTTAAGAGT
GCTAAGTTAGGTGACACTGTTCTTGCCGAAGCTACACCTGTCAACATCGGTAAAACTATTCAGGTCTGGGAGGTGAACTTATCGAA
ATGTGATCCTTCGGATTCCGAGATTAAAACGTTGATCTCATCTTCAAGAGTCACTCTTCTTTGTAACTTGCCTGTGCCAGAATCAC
TCAAGGCTGCTGCTCAAGGTCTCAAAAAGTACGCAAGACTGTAATAATAAGCTCAAAATTTAGTACTGTTATTATAATCTCTGAAA
CAACTCTATGTTGTGAAAAAAACGAAAGAAAGTATCGAAATATAACTAAAATGATGTAATTTACAGAATTTATGTTATACGCAGGT
CAACTTTTCATTCAGATGCTTTAAAAACTCTCAGCATTAAGTTGGGGTCTTTCACAATCTTCACATATTGAAAGAGGCATAATCTT
GGATCCAACACATTTTGTGCGATACATAGTATTTATTCATATCAATTCTTGTTTGGGGATCAACAATGTGGAATTTTTCGGACCCA
TTTACTCTCTTTGAGCATCTCGAGCAGAGCGATGGAGATGCGTTTTTTCGCAAAATGGAAGCAAATTGGGGGTCGCAGTGCGCTAT
TTATTTGCGCCAGTCTTGTGCTTGGAATAGTCCTGCGCCATTTTCAGCGCAGGACTCTAGTGCGCTATTTTTTTTTATATACTTTT
TTGTTGTTTATATTTCTTTTTTTCTTTTAATGTAAACAATTTAATATTAAATTATTATATCTAATATTATAATTATAGAAAAAAAA
AAAAAAAAA
>ShDHNAT1 Striga hermonthica Sh.c83733_g1_i1.18685
GTCCATGCAGTACCATCATCCTGAATTGTCATTATTGTCTAAATGTAAGAGAGCTACAATAACAAACATGTATCACCCACATACAA
TCAGATTCTAGACTACACCACGGTGGCTTTTCTACCCTATCTCAGTTTTTCTTCTTCTCCTGTTCTTGAAATTCGTTAAACTGATC
TACTCAGAGTAGATTGGTCTGCGAATAATCTAAAACCATAAAGACACAAGAAGCGACTCAGCAGAGAGAAGATGAACCGACCGCTG
CCATTAAACACAAAGGATTTGGATATTCCACTCCACACGATTGGGTTTGAAATTGACTGCATTTCGCCTGAAAAGGTCTCGGGCCA
TGTTCTGATCACTGAAAAGTGCTGTCAGCCGTTCAAGGTGCTGCATGGAGGAGTTTCGGCACTAATAGCCGAGGCTCTAGCAAGCA
CTGGGGCCTACCTTGCTTCAGGCCTTCAGAGAGTAGCTGGCGTCCACCTAAGCATCAATCACCTAAAGAGTGCAAAGTTAGGGGAC
TATGTTGTTGCGGAGGCTACTCCCGTGAACATCGGGAAAAGTATTCAGGTCTGGGAAGTGAACTTATCAAAATGCGATCCTTCGAA
CTCAGAGACAAAAACATTGATTTCTTCTTCAAGAGTTATTCTTCTTTGTAACTTGCCTGTGCCAGATTCATTAAAGGAGGCTGCTC
AAGGTTTCAAAAAGTACTCCAAACTCTAGATAAGTTCAAAACCGATTTTCTTCTGTAATTTTCCGAAACTATTGTGTTGTAATAAG

ATAAAAGGAAAGCAAAAAGGGAAGGAAAATGAACCCTTTTGCAATTACAGCAAAATGTCACCCTACCAAATATGCAGAATATATGC
TAGGCAGTTGGCACAAGTTATTGTTCGGTTGGTCTGTGATAGCAATTAGCAAGGTGGAATTTGTCTTACCAGCATATGATATATTA
TGCTTCGTATAAACTATGACAAGTCTATTTGAATTTTGTGGTTATGATATATTGGATAACAATATATTGCTCTTAGTTTTGATGTT
GTTGTTTTTGTTCCGAAAATTGCTCTTAAATAGTAATCACATTGCACTACAATTAAGAATACAATACCATGAGGATTTCATTTGAG
ATCATGAGATCATAAACTATGTTATTACACACTTCAAATGAATGACATAACATGACTAT
>ShDHNAT2 Striga hermonthica Sh.c13237_g2_i1.9518
GGGATAATAGATAAAGCTGCCATTTTTTTTTCTTGCTTCAGCTTCTCTACCACATTCCCAGATAAATTGTTTTGTGGTATAGTAGT
GTGTGAATAATATACAAAACCACAAAGGCACGAGAAAAAACTCGATTTCTATCCCAGTCGACCGAGCGGAGATGAACAGAACACCA
TCCGCCACAGGGCCTCCGCCACCACCACCATCAAAGACCAAGGAATTGGATATTCCACTCCACACGATCGGCTTTGAAATCGATTG
CCTTTCGCCTCAAAAAGTTTCGGGCCACGTTTTGATTTCTGAAAAGTGCTGCCAGCCGTTCAAGGTGCTTCATGGAGGTGTTTCGG
CACTGATAGCCGAGTCTCTAGCAAGCATTGGGGCCCACTTAGCTTCAGGCCTTCAGAGAGTGGCCGGCGTACACCTCAGCATCAGT
CACTTAAAGAGTGCAAAATTAGGCGATTTTGTTGTTGCCGAAGCTAAGCCTGTGAACATAGGGAAAAGTATTCAGGTTTGGGAAGT
GAACTTATCGAAGTCCGATTCTTCAAACTCTGAGACCAAAACGTTGATTTCATCGTCAAGAGTTACTCTTCTTTGTAACTTGCCTG
TGCCCGAGTCACTCAAGACTGCTGCTCAAGGTCTCAAAAAGTACGCGAAACTCTAAATAAGCTAAAAACCGGTTTCTTTTGTATTG
CTAAAACTATTTTTTATGTAAATAAAAGACAAAAGGGAATGACAATGAGCCCTTTGCAGCTATAGCAATTATCACATCACCCTACC
CAATTTGCATAATCTATGTTTGGCACAAGTTATTATTGCTCAGATGATTAATAAATGTCTATTTGATGACAATTATCAGTCGATTG
TTCGCTCTATCCATCGAGCTATAATGTAAATTAAATAGAAAAAAAAAATACTGTGTGAATTTTTCCAGTTGAACGCA
>TvDHNAT1 Triphysaria versicolor Tv.c6470_g1_i1.9012
CCTTATTATTTTTTATTATACATATCAATGGAAGATTAAACTGTGCACCCGATTGTTTATTTCGTTTGAATCTTACTGTGCTCTGT
CTTGTATTTTGGTTTTCTGCTGTTTCTTTGCCTGGTTTCTGTTCTCTCGAGTTGTCAAAAACGCCCATTTATTCGAGAGGATTGTG
TTTTGGGCTGTCAAGGTTTCAATCAATTAAAAACGCATAAAGTATGAGTGAATCACTACCACCAGCAGTAAAAAGGATGGAGCTTT
TGGATGCTCCACTTCACTTATTTGGCTTTGAACTCGATGAACTTTCGCCTCATAAAGTTTCCGGCCACCTCTTAATCACTTCAAAG
TGCTGTCAGCCATTCAACATGCTGCATGGAGGAGTTTCGGCTCTGATAGCTGAGGCTTTGGCAAGCATAGGAGCTCATATGGCTTC
AGGGTTTCATAGGGTAGCCGGTATACAACTGAGCATCAATCATCTAAAACCCGCTCAGGCCGGAGATTTTGTAATTGCTGAGGCCA
CACCAGTCAGTGTTGGTAAATCTGTTCAGGTCTGGGAGGTGCGTCTCTTCAAATGCGATCCTTTGAAATCCGATGAGATTAGAACG
TTGATTGCGTCATCGAGAGTTACGGTATTCTGTAACTTGCCTGTACCAGAATCTTCAAGGGATGCTGCTCAAAATCTCAAAAAGTA
TTCGAAGCTATAAATCAGTAATCTTGGTCAATGATTATCGGGTTTTTCACATCTTTCATCTGTATGTTTTGAAACCTGATGTTGCT
TGCACTTCCAGATCCTCCCTGTTCATGTATATATATTTCAGGTCCACAGGTAATGTCTATGGCTCGAACATTCTTCGATCCATCGA
CGATTGAAATGCAGTCGTCGCCTAATTGATACGATATTAATTAACAAACGTGTTACTACAAATTAATTCGAAAGTAATATATATTA
ATGAAGAAAATATCGTACCGGTGCCAATAGTACAGCTAGAAATACGGATATTTTGAGTGGCAGTGACATGTATGCCATCAGTGTTG
GGGCTTTCATCAGGAGCAGTCACAACAAGTTTTGAAGCTTCAACATTTTTGCACCTTTGAAATGACAATTGCATTTGCTGTGCATT
TTTTATCTTCAGATTTTGTACTACCAAGTCTATGCACTTGTAGAAGGTTATAGCCTGCACAATTCATTTTCTTCATTAATATATAA
TCGTAATTATATAAATTGATAAATAACACCATTTTATGCCATGAACAAGATGCCTATATACATAAAAAATTTAAAAAT
>TvDHNAT2 Triphysaria versicolor Tv.c6736_g3_i3.9225
AAATCATTTTTTTATTGGAATTCAATCCTTAGTGTCAGAGATGATAATAATAGACCCATACGAGTTTGCGGGCCATCCATTTACTT
GGATTTTTTTTTCGATTTATCGTCCAAGTGGAAAAACACAACAACAATATGATTTGTGAAGCTGCTATATTTTCTCCGCCATTGTA
GACTAGAACTCTGCTTCTTCATTGCCCAATATACAAATTAAAAGCATCTATAGTAGATTTGTTTCCTAATTGAGAGAAGAGCCGAG
AAGATGACCCAACCACCATCTGCCGTCGGGCCGCCGCCACTACCACCACCAGCGAAGACAGAGGAATTGGATTCTCCGCTTCACTT
GATCGGCTTTGAAATCGATTGCCTTTCGCCGGACAAAGTTTCTGGCCATATTATTATCACTTCAAAGTGTTGCCAACCGTTCAAAG
TGCTTCATGGAGGTGTTTCGGCACTAATAGCCGAGTCGCTAGCAAGCATAGGAGCTCACCTTGCGTCCGGTCAGCAACGAGTTGCC
GGTGTACACCTCAGCATCAATCACCTTAAGAGTGCAAAGTTAGGTGATTTTGTCGTTGCCGAGGCTACCCCTGTCAACATCGGCAA
AACTATTCAGGTGTGGGAGGTTCGTTTATCGAAATGCGACGATCCTTCAAACACTGAGATAAAAACGTTGATTTCGTCATCAAGAG
TTACTCTTATTTGTAACTTGCCTGTGCCTGAATCGCTCAAGAGTGCTGCTCAAGGTCTCAGAAAATACGCCAAACTATAATAAGCT
CTTGTTTTTTGAGCATATAATAAACCCCAAATCGAGTGTTGTTGTAATCTCTGTAACAATTTAATGTTTTTTTTTTTTTGGAAATA
TATAGCAAACAGTGCTGTTTGTGGAACCGGAACCTATGTATGTTACACGCACGTCAAATTTCATTTATATACTTGCGCAAACGGCA
TATTACGAGACTCTGCTTCAATTTCATGTGATGGTATATAAATTTATTTGAATCATCATTTCTGCAAAACAATTTGCATATGACAA
ATACATGAGGATAT
>TvDHNAT3 Triphysaria versicolor Tv.c102567_g1_i6.16017
GAGAAATGGAAATCTCATAGGGTTAAAATCTGGGAAGAAAAAAGGCGTTAAAATAAAAAAAAATCTCATATTATTTGGTTGTCAAG
TAAACTATAATCAAGAATCACACCCCGTTTCCACCCTATTACGTTTTCATGTGCGCAAGTGGTGTAGAAAGAGACACAACAAAGAA
AAAGGCGCAGGTGAAGATGAAAAAGCCCTAAAGCAGCAATTTTTATTAGGATTTTTGATTATTTTATTCCCAAATCCTGTGGATAT
GATTCCGCTTCTGCTCCTATTTCTACTTCACTGCCCAACAATCCCTTCATTTTTCTTCGGTATTGTGCACTGATTTCCAGAACTAT
AACGAATACAAAAACTTGATCCATCTCAGCCGAGAGAGGCGATAAGATGAACCAACCACCACCTAATGCCGGACCGCCACCACAAT
CAATGACGGAGAAACTGGATGTTCCGCTTCACTGGCTCGGTTTTGAGATCGATTGTCTTTCGCCTGACAAAGTTTCAGGACATTTT
ATCATCACTTCAAAGTCGTCTCAGGCGTTCAAAGTGCTACACGGAGGAGTTTCAGCGCTGATAGCCGAGTCACTAGCAAGCATAGG
AGCTCACCTTGCGTCCGGTCAGCAACGAGTGGCCGGTGTACACCTCAGCATCAGTCACCTTAAGAGTGCACAGCTAGGTGATTTTA
TCGTTGCCGAAGCTACCCCTGTCAACATCGGCAAAACTATTCAGGTGTGGGAGGTTCGTTTATCGAAATGCGATGATCCTTTAAAC
ACTGAGACGAAAACGTTAATTTCGTCTTCGAGAGTTACGCTTCTTTGTAACTTGCCTGTGCCTGAATCTTTCAAGACTACTGCTCA
AGGTCTCAAAAAGTATGCAAAACTATAATGGCTATATAAAATTATTTGAACATTATTATTATGATTAAATCTGTCTTGGATTTATT
TATGGGTAAATTGGACATTCAGTTCCTGTAGTATAGTGAAATTGCAAAGTCTGTGTTCAATGTTTTGATAATTGCACTTTCAATAC
CTAAAATTTGGGTAGTGACAGTGAAACAGTGGTTACTGAAATTGTTGTTTTTCTAAAAGTTTAGGTGTTGAGAGTGCAATTATTAA
AATATTTGGTATCGACTTTGTAATTTTAGTAT

>PaMenA2 Phelipanche aegyptiaca Pa.c24509_g1_i2.9208
TAAGATTCTATTTATATCACAGCCGCTCAGTTTTTCTCTCTTTTGTTTTTATATTTTTTCATTTACCAAGCTTGTATAACCAGGAC
CAGATCCCAAGCAAATTAAGATTCTATTTATATCACAGCTCATTATTTTCATACACTTGTGGTTTATTTTTTCATACTTTTACTTT
AGTTTGACCCTCGACAATGGCGGAAGCGGCAAACCATCCAAACCAGGCAAACATTGTTAAAAGATTACATAAGAAAGAGAAAAAGG
AAGGTGATATATCTCGAGCAACTTTAATATGGAGAGCTGCCAAATTACCTATGTACACCGTTGCATTAACTCCTCTAACTGTTGGG
ACAGCTGCTGCATATTGGGAGTCGGGATTTTACTCTTTGGAGCGTTATTTCACTCTCTTGGCGTCTTTTGTTCTTGTCAATCTTTG
GATCAATTTAAGCAACGACGTTTACGATTTTGATACTGGAGCTGATAAAAACAAAAAAGAATCTGTTGTCAATATATTTGGCAGTC
GCACGGCCATCCACATTCTTTCGCTGTTAGTACTTGCAATTGGTTTCGCGGGGCTTTGTTGGGTAGCTCTTGAGGCTAAAAACCCG
CGCGCTATTCTATTATTGGCTTCGACAGTATTTTGCGTCTACGCTTACCAGTTCCCACCGTATCGTTTAAGCTATCACGGACTGGG
AGAGCCCTTATGTTTTGCAGCATATGGCCCTTTTGCCACAGTTGCTTTTTATTTGCTCCAAAGCGGCTCTTCAAGTGAGCTACCCA
TATCTAGCACGGTTGTATTTGCATCAATTTTTGTTGGTTTTACATCAGCTTTGATCCTCTTCTGTAGTCATTTTCATCAGATAGAG
GATGATAAAGCTGTTGGGAAAATTTCTCCTTTGGTGAGGCTTGGCACTGAAAAAGCATCAAAAGTTGTGAAAATGAGTGTTTTAGG
GTTCTATTGGCTTGTGTTTGGTTTAGGACTGTCCCAAACACTTCCTTATGCTTGTGTAGTGCTCTGTACTATGACACTGCCCATGG
GAAACTTAGTAGTTAGCTTTGTGCAGGAGAACCACAAGGATAAATCCAAGATCTTCATGGCTAAATACTACTGTGTAAGATTACAC
ACTGTATTCGGAGCTGCTTTGGCTGCTGGACTGGTGGCATCAAGATTAATAATGCATTCTGGAGAGCAACTTCAGCAGAGTATTTT
CGATTATGCCAAGTTTCTTTATCTCTGAGTTTATGCATTTGTATTTCTCAGTCTTTGCACACTAATAAGGAAAACTGATTCTCAGC
TTTTAGGTGGACAAGATGAATCTTCTAGAAGCTCAGAGTCGTTTTTCTTTTTTTCGTAAAATTCGCAAACACTCTCTTTAGAAAGA
GTTGGAATTAGAATTTATTGCTGCCCCAAGTTAAAAGTTTCTTAGAATGATTTCCTTTATATATATTTATATATATATTTTTATAT
TTTCTGTTGCAAAAAAAAAAAAAAAAAAAAA
>ShMenA1 Striga hermonthica Sh.c15001_g1_i2.8280
TTTTTTTTTTTTTTTTTTTCTGCTTTATTCAAGCCATAGAGCTAGATTTATGGCGCCTCTTGCTGTGGCAGCAGCTGTGTATTGTT
CCACAAGCCATGGCTATGGCGTCAAGAAGCTCGACGATTATCTTACCAGAAAACTCAGTATCAGCAGGATTCATCAAGTATTACTT
CTTCCAGATGCCTGTCAAAGGTCACTATGCACAAAATTTAATTTCAAGAAGGCCAGCATGAGGCAATTATATTCTCTACGGGGGCA
TTATATTAATTCATTTAAACAAAGAGCAGAACACTGTGGGAGTAGTAACATTGAAGAAAACAAGGAAGAAAGCATCTCCAGGGTAG
CTTTGATGTGGAGAGCTATCAAATTACCCATTTACTCTGTTGCATTGATCCCTATAACAGTTGGAAGTGCAGCAGCATATTTGCAG
ACGGGCCAATTTTTTGGAAAGCGTTATTTAAAGCTATTGTTATCATCAGTTCTCATCATAACGTGGCTCAACTTAAGCAATGATGT
ATATGATTTCGAAACTGGGGCTGATAAAAACAAGAAAGAATCAGTTGTTAATCTAATTGGCAGCCAAACAGGAACTCATATTTTAG
CTTGGGTACTACTTGAACTTGGTTTCGGGGGCCTTACTTGGGTGTCTATTGAGGCTGGAAGTATACGTTCTATAATTCTACTTGCC
TGTGCCATATTTTGTGGCTATATTTACCAGTGTCCACCTTTTCGGCTGAGTTACTTGGGACTTGGGGAACCTTTGTGCTTTGCGGC
ATTCGGTCCATTTGCTACCACTGCCTTTTACTTGCTCCAAAGCGGGACAAGTGAGCTGTCAATCTCTGCAACTGTAATCTCTTCAT
CAATTCTTGTCGGCTTCACAACGTCCTTAATCCTATTCTGTAGCCATTTTCATCAGATAGAGGATGACAAGGCAGTCGGGAAATTT
TCGCCTGTGGTCAGGCTTGGAACCGAAGGAGGTGCAAATGTAGTGAAAGTGGCCGTACGGATACTTTATTCGCTTCTATTTGTTCT
CGGGCTTGCCCAAATTCTTCCTTTCCCCTCTATAGTTCTCTGTGCTTTAACATTACCAGTTGGAAATTTAGTGGTTAGCTTTGTCG
GGACAAACCACAAGGACAAGATGAAGATATTCATGGCGAAATATTACTGCGTGCGGTTGCACACGATATTCGGGGCTGCATTAGCT
GCTGGGATGGTAGCGGCCAGAATGTTTGCGAGAAAGCAACTCCCGCATGCTATTATTCTTTGAACTATTGTTGGTGCTCATTTGTA
TAAATTTTCGATGTTCTTAAAACTGGACTGACCATTGAAATAAAGCTACAATGGACTGACCATTTAATCAAGAAGATTATATAAGA
ATTGATTTTTCAATAACTCAACCGATTCTATTTTTTTGTTTTTTTTGAATTGTAAACGAAGTTTTCCACAATATCAAAATTAACTA
TTTATCTTTAATACAAATCAATCTTTATCCTTAATAAGTAAAAAAAAAAAAAAA
>TvMenA1 Triphysaria versicolor Tv.c6621_g2_i2.1348
CTCCAGCATTGTGAGGTTGAAATTTGTCCATGTTTTACTTATCTTCTTCGTGACCCAGAATTAGTTACTCTCTGTTCCAGCTAACA
TGGTCTTTTCACATTTCTTAATCCTCACCATTTTTCTTCTTCGATTAGTATATCGTTGATCTACTGTTCACTTTCAAGCTGTAAGG
TTTGGCGCCTGTGGCTATGGCAGCGGCCACGTTCTGTTCTATTAGTATTAGCCATGGCTATGCCGTCCAGAGACTCAACAGACACA
AAATCAACAGGACTTACCAAGTATTACCTCTTGTATGTGGCAGTCGAACCACGAAAGTTCATTTGAACAAGACAATCATAAGACAA
TATTTACATTCTATACAAAGGCGTTACAAAATCTCGCCCAAGTTTAGATCAGAGAACAATGCAGACAACACTCACGTCGAAGAAGA
AGAAGAAGATGAAGATGAGCAAGAAAGTGTATCTAAGGCAACTTTAATTTGGAGAGCCATCAAATTACCAATGTACACTGTTGCAT
TGATTCCTATAACGGTTGGAAGTGCAGCAGCTTATCTACAGACAGGACAATACTTTGGAAAGCGTTATATTATGCTATTGGTTTCT
TCGGTTCTCATCATAGCTTGGCTCAATTTAAGCAATGACGTTTACGATTTCGATGCTGGAGCAGATAAAAACAAGAAAGAATCAGT
TGTTAATCTATTCGGAAGCCGGACAGGAACACATGTTTTTGCATGGCTGTTACTCGCACTTGGTTTCGCGGGCCTTGCTCGGGTTT
CTGTTGAAGCTGGGAGTTTACGTTCTATATTTCTACTTGGGTGTGCCGTATTTTGCGGCTACATTTATCAGTGTCCGCCGTTTCGA
TTAAGTTATATGGGACTTGGAGAGCCCTTATGCTTCGTTGCATTTGGCCCGTTTGCGACCACAGCCTTTTACTTGCTTCAAAGCGG
GACAAGGGAGCTGTCGATTTCTGGCATCGTTATCGTTTCGTCGGTTCTTGTTGGTATCACAACATCCTTAATTCTATTTTGTAGCC
ATTTTCATCAGATTGAGGATGATAAGGCTGTCGGAAAATATTCGCCTTTGGTTAGGCTTGGATCCGAAGGAGGTGCTAACGTTGTG
AAAATGGTTGTTAGGACGATTTATTCGCTTTTATTTATTTTGGGACTGGTCCAAACCCTTCCATTCGCGTCCATTGTTCTTTGTGC
TTTAACATTGCCGGTTGGAAATTTAGTTGTTAGCTTTGTCGAGAAGAACCACAAGGACAAGACGAAGATCTTCATGGCAAAATATT
ACTGTGTGAGATTGCATACAGTATTCGGAGCAGCATTGGCTACTGGGCTGGTTGCAGCTAGAGTGCTTGCAAGAAAGCCAATTCCT
AATGCTATTATTCTTTGAGCTTTTCAGTCCTACAAGGTGGTGATGAGGATTGTTAGTTATTTCGATTACACTTTTCAAAGAAAAAT
AAATAAAATAATCATTCATGTAATTAAATTTGTGTCATTATCACTTGTTAATAACTTGTTGATAAAATACAATCAACAATAAATGT
TTAACAATAATTCCAAATCAATCAAAATCTGAATTTAAACAAAAAAAAAAAAAAAAAAAAACAAATCTACACCGCAAAAATCTACTA
CTCGTTATCGGGCTTAGGGGCTGCTTCCTTGATCTCCTCAGAGTTATCATCCTGCATGTCGGAAGTCCACAAAGTGAGGTTGTCAC
GGAGAAGCTGCATGATCAGAGTGCTATCCTTGTATGATTCCTCCCCAAGTGTGTCTAGCTCAGCAATTGCCTCGTCAAAAGCCTGT
TTGGCGAGATTACAAGCACGATCAGGAGAATTCAAAATCTCGTAGTAAAAGACTGAGAAGTTGAGTGCAAGTCCAAGATCGGAAGA
GCACACGTCTGA
>CaMenA2 Conopholis_americana FAMO-2092133 (1KP)

TTTTATTTTTCCTTTGATAATGGCAGGAGGAGCCAACTTCATCAGTTTGGCAGACAATCCAAGCCTGGAAAAAAATGTTAAAAGAT
CAAAGAAGAAGGGAGAGAAAAAGGAAGAAGAAGATATATCTAGAGCAACTTTAATATGGAGAGCTGCAAAATTACCTATGTATACT
GTTGCACTAATTCCTCTAACTGTTGGAACAGCCACTGCATATTGGGAGTCAGGGTATTACTCTTTGGAGCGTTATTTCATTCTCTT
GGCATCTTTTGTTCTTGTCAATGTTTGGATCAACTTAAGCAACGATGTTTATGATTTTGATACAGGAGCTGATAAAAACAAAAGAG
AGTCTGTTGTGAATATGGTTGGCAGCCGCACGGCCATCTATATTCTTTCATGGTTACTACTTGCACTTGGTTTCGCGGGACTTTCT
TGGGTAGGAATTGAGGCTAAAAACCCGCGGGCTATACTATTATTGGCTTCATCAATCTTTTGCGTCTATATTTACCAGTGTCCACC
TTATCGTTTAAGCTACCATGGACTGGGAGAGCCCATATGCTTCGCCGCATACGGCCCTTTTTCCACCATTGCTTTTTATTTGCTTC
AAAGCACTTCAAAATTTGAGCTACCGATATCCAGCACGATTGTATCGGCAGCAATTCTTGTTGGTTTTACAACAGCCTTGATCCTC
TTTTGTAGTCATTTTCATCAGATAGAGGATGACAAAGCTGTTGGGAAAATTTCTCCTTTGGTGAGGCTTGGTACTGAAAAAGCATC
AAAAGTAGTGAAAATGGCTGTTTTGGGGCTCTATTGGCTTGTTTTTGGTTTTGGACTGACCCAAACACTTCCTTATGCTTGTTTAG
TGCTTTGTACTATGACACTACCCATTGGAAATTTAATAGTTAGCTTTGTTCAGGAGAACCACAAGGATAAATCCAAAATCTTCATG
GCTAAATACTATTGTGTGAGATTACACACCGTATTCGGAAGTGCTTTGGCTGCTGGACTGGTGGCATCAAGAATGCATTCTGGAGA
ATTAACTTCAGAGTTTATTAACGTACTTCGTGCCACGAAGTTTGCGTACTTCTGAGTTTATGCAATTGTTCATGATTTAGTGATAT
TGACGTACTTTGATTGTGCCAAGTTTGCGTACTTCTGAATT
>AfMenA2 Aphyllon (syn. Orobanche) fasciculata VYDM-2034145 (1KP)
CAATACACTTCGTGATTTATTCTTTACTTTTACTTTAATTGGCAATCGACAATGGCGGGAGGCAGCTCTATTAGTTTGGCAGACAA
CCCAACCCAGGAAAAAGTTGTTAGAAGATCAAAGTCAAAAAAGGAAGAAGATATATCTCGAGCAATTTTAATATGGAGAGCTGCCA
AATTACCAATGTATACAGTTGCATTAATCCCACTAACTGTTGGAACAGCCGCTGCATATTTGGAGTCAGGCTTTTACTCTTTGGAG
CGTTATTTCATTCTATTGGCGTCCTTTGTTCTCGTCAATGTTTGGGTCAATTTAAGCAACGATGTTTATGATTTTGATACAGGAGC
TGATAAAAACAAAAAAGAGTCTGTTGTCAATATAGTTGGCAGCCGTACGGCCATCCATATTCTATCATGGTTACTACTTGCACTTG
GTTTCGCGGGTCTTTGTTGGGTAGGACTCGAGGCTAATAACCCGCGCGCTATACTATTATTGGCTTCGGCAATCTTTTGCGGCTAC
ATTTACCAGTGTCCACCGTTTCGTTTAAGTTACCACGGACTGGGAGAGCCCTTATGCTTTGCATCATTTGGCCCTTTTTCCACAAT
TGCTTTTTATTTGCTGCAAAGCAGTTCAAGCGAGCTACCGTTATCGAGCACAATTGTGTCTGCAGCGATTCTTGTTGGCTCTACGT
CAGCCTTGATCCTCTTCTGTAGTCATTTTCATCAGATAGAGGATGATAAAGCTGTTGGCAAAATCTCTCCTTTGGTGAGGCTTGGT
ACTGAAAAAGCATCAGAAATTGTGAAAATGGCTGTTCTTGGGCTCTATTGGCTTGTGTTTGGTTTAGGACTGTCCCAAACACTTCC
TTATGCTTGTATAGTGCTCTGTACTATGACAATACCCATGGGAAACTTAGTAGTTAGCTTTGTTCAGAAAAACCATAAGGATAAAT
CGAAGATCTTCATGGCTAAATACTACTGTGTGAGATTACACACTGTATTCGGAGCTGCTTTAGCTGTTGGACTGGTGGCATCAAGA
TTAATGCATTCTGGGGAGCAATTTCAGAGTCTATTAACGTATTTCGACCGAGCCGAAATTGCGTAATTCTGAGTTTATGCATTTGT
ATTCCATGATTCAGTTCAAACTCTAAATAAGTAAACAGAGAGAGAAGTGATTCTCAGCTTTTAGGTGCGGAAAATGAGAGTTTTGG
AAGCTGAGAGTGGCATTTTCGTAAAATGGGCGAACACTCAATTCAGCAATATGGTTCTCAGGCTTAGATAGAGTTGGGATTGGAAA
TTATTGCAGCCAGAAGTTAAAAGTTTCTCAGGA
>RgMenA1 Rehmannia_glutinosa OWAS-2001018 (1KP)
CGCCTTTGGCTATGGCAGCAGCCACGTTCTGTTCCATAAGCCATGGCTATGGCGTCAACAAACTCAATCAACACCTTCTCAACAGA
CACAATTTCAACAGGATTTATCAAGTAATACCTCTTCCAGATGCCAGTCGAAGGCAACTACGCATGGAAATGTGTTTCGACAAGAC
GATCATAAGGCAATTACATTGTATACGAAGGCATTATGGTATTTTGTTGAAGTGTAGATCAGAGCACAATGACAGTCATGTTGTAG
AAGAGAAGGAAGAAAGTATCTCTAAAGCAACCTTAATATGGAGAGCCATCAAATTACCAATGTACACTGTTGCATTGATCCCTATA
ACAGTCGGAAGTGCAGCAGCTTATTTACAGACAGGCCAGTATTTCGGAAAGCGTTATTTAATGCTATTGATTTCTTCAGTCCTCAT
AATAGCTTGGCTCAACTTAAGCAATGACGTTTACGATTTCGAAACTGGAGCGGATAAAAACAAGAAAGAATCGGTTGTTAATCTAT
TTGGCAGCCGGACAGGAACTCATGTTTTTGCTTGGTTACTACTTGCACTTGGTTTCACGGGCCTTACTTGGGTTTCTGTGGAGGCT
GGAAGTCTACGTTCAATATTTCTACTTGGTTGTGCCGTATTTTGTGGCTACATTTACCAGTGTCCGCCGTTTCGTTTGAGTTACTT
GGGACTGGGAGAACCCTTATGCTTTGCAGCATTTGGTCCATTTGCGACCACCGCCTTTTACTTGCTTCAGAGCGGTACAAGGGAGC
TCTCGATTTCTGTCACCATTGTTTCTTCATCAATTCTTGTTGGTTTAACGACATCCTTGATCCTCTTCTGCAGTCATTTTCATCAG
ATAGACGATGACAAGGCTGTCGGAAAATTTTCGCCTTTGGTCAGGCTTGGATCTGACGGAGGTGCAAAAGTAGTGAAAGTGGCTGT
CGGGACGCTCTATTCGCTTCTATTTATTCTAGGACTTAGCCAAACTCTTCCTTTTTCATCTATTGTTTTATGCGCGTTAACGTTAC
CCGTTGGAAAATTAGTAGTTAGCTTTGTTGAGAAGAACCACAAGGACAAGACGAAGATCTTCATGGCAAAATATTACTGTGTGAGA
TTGCACACAGTATTCGGAGCTGCATTGGCTGCTGGGATGGTGGCTGCTAGAATGTTTGCAAGAAAGCAACTTCCTCATGCTATTAT
TCTTTGAACTTTCCGAGTCGAATAAGGAATTAATATTCTCAGAGTATCCAAGCTCCATACAATTGGTGGTGTTTGATCGAATGAGT
GACGATATTATTTGAAATAGAGGATCATGTGGAGGACTAATGGTGCTGTCCCATTTTTCTCTCTTTTTTTGGGGGCTTTAATTTTG
TATTTCAGTTTATGCATATGTAGATTTAGGAGGGCC
>RgMenA2 Rehmannia glutinosa assembled from SRR999317
CTTTAATTTATTTGGCCTTTTAATTGAAACAATGGCAGGAACCAGCTTCAGTTCGTCTGACAATCCAAGCAAGGAAAAAAATGGTA
AAAGATCAAAGAAGGAGACTACTTCCCATGACGAGAAAAAAGAAGAAGATATAGGTAGAGCAACTTTAATATGGAGAGCTGCCAAA
TTACCTATGTATACTGTTGCATTGATTCCTCTTACTGTAGGAACTGCAGCTGCATATTGGGAATCAGGCTACTATTCTTTGGAGCG
TTATTTCATTCTCTTGGCATCTTTTGTTCTTGTCAATCTTTGGGTCAATTTAAGCAACGATGTATATGATTTCGATACAGGAGCTG
ATATAAACAAAAAAGAATCTGTTGTCAATATAGTTGGCAGTCGCAATGCCATCCATATTCTTTCGTGGTTATTACTTGCCATTGGT
TTTGCTGGCCTTTCTTGGGTCGGAGTCGAGGCTAAAAACCCGCGTGCTATACTCTTATTGGCTGCGGCAGTCTTTTGTGGCTACAT
TTACCAGTGCCCACCGTTTCGTTTAAGCTACCACGGTCTGGGAGAGCCCTTATGCTTTGCATCGTTCCGCCCATTTTCCACCGTTG
CATTTTATTTGCTCCAGAGCAGATCAAGTGAGCTACCGATATCTAGCACGATTATATCCGCAGCAATTCTTGTCGGTTTTACATCA
GCCTTGATTCTCTTCTGTAGTCATTTCCATCAGATAGAGGATGATAAGGCTGTCGGAAAAATTTCGCCTTTGGTGAGGCTTGGAAC
TGAAACAGGATCAAAAGTAGTGAAAATGGCTGTTTTGGGGCTGTATTGGCTTGTTTTTGGTTTAGGACTGGCCCAAACACTTCCTT
ATCCTTGTATAATGCTCTGCATAATGACACTACCCATGGGAAACTTAGTAGTTAGCTTTGTTCAAGAGAACCACAAGGACAAATCG
AAAATTTTCATGGCTAAATACTATTGTGTGAGGTTACACACTGTGTTCGGAGCTGCTTTGGCTGCTGGACTTGTGGCATCAAGATT

AATACAATCTGGAGAGCAACTTCAGAGTCTATCGACGTACTTTGATCGTGCCAAGTTTGCGTATTTCTGAGTTTAATAGTATATTC
AGAAGTCCAACCTTTTGCTGGGAA
>LpMenA1 Lindenbergia philippensis ZVFS-2005540 (1KP)
GATTTAGTGATCTCAAACAAAATGGGTTTTTTCATAATTCTTGATCCTTAATTCCTTATCCCAATTTTTCTTCCATTATTTACATT
TTCTTCAAAAAAAAGAAAAATTGATTGGCGCCTTTGGCTATGGCAGCAGCCACTTTCTGTTCCATAAGTCATGGCTATGGCGTCAG
CAGAGTCAACGACTATCTTCTCAACAGACACGGTATCAACAGGATTGATCAAGTATTACCTCTTCCAGATGCCAGTCGAAGGCGAA
AACTTGTGAAAAAAGATTTCAACAAGACAGTCATCAGGCAATTACATTCCATACGAAGGCATTATACAATGTCGTTGAAGTTTAGA
GCGGAGAACAATGGCGATCGTATCGAAGAAGAGAAGGAAGGAAATGTCTCGAAAGCGACCTTAATATGGAGAGCCATCAAATTACC
GATTTACACTGTTGCATTGATCCCTATAACAGTTGGAAGTGCAGCAGCTTATTTGCAGACAGGCCAGTATTTTGGAAAGCGTTATG
TTATGCTCTTGATTTCTTCAGTTTTTGTCATAGCTTGGCTCAACTTGAGCAATGACGTGTATGATTCCGAAACTGGAGCGGATAAA
AACAAGAAAGAATCCGTTGTTAATCTATTTGGCAGCCGGACAGGAACTCATGTTTTTGCTTGGTTACTACTTGCACTCGGCTTCAC
GGGCCTTACTTTGGTGTCTGTCGAGGCTCGAAGTCGACATTCTATATTTCTACTAGCTTGTTCCGTCTTTTGTGGCTACGTTTACC
AGTGTCCACCATTTCGGTTGAGTTATTTGGGACTTGGAGAACCCTTATGCTTTGCAGCATTTGGTCCGTTAGCTACCATCGCCTTT
TATTTGCTTCAAGGCGGTACAAGGGAGCAGCTATCAATTTCTTGCGCTGTTGTTGCTTCATCAATTCTTGTTGGCTTCACAACATC
CTTGATACTCTTCTGTAGTCATTTTCATCAGATAGAGGATGATAAGGCTGTCGGCAAATTTTCACCTTTGGTCAAGCTTGGAACTG
AAGGAGGTGCAAAAGTAGTGAAAGTGGCTATAGGGACAATTTATTCGCTTCTATTTATTCTCGGACTTAGCCAAACACTTCCTTTC
TCATCTATTGTTCTCTGTGCTTTAACGTTACCTATGGGAAAATTTGTTGTTAGCTTCATCGAGGAGAATCACAAGGACAAGACGAA
GATCTTCATGGCAAAATATTACTGCGTGCGATTGCACACAGTATTTGGAGCTGCATTGGCTGCTGGGCTGGTGGCAGCTAGAATGT
TAGCAAGAAAGCCCCTTCCTTATGCTGTTATTCTTTGAATTTTTTAAGTTACGTAAGGTGATGGGGATTGTTATCTCGATTAATGT
TCAGTTTTTTTTTTTCTCTTCTTCTAACAAACAGTATGCAAATCTGGCATTGTTCAACATATGTTATCTTTCCCTTACACGACATA
ATTTTTAGGGTGAATTACAAAAACTACATGTGAGGTTTTTGATCTAGTGTTTAAAGTAGGAGGTTAGCTGTAATATTTTTATGAAT
GTAAGGTGGTATTTTGAAACATTTTTTCAGTTATAAAAGGATAAATGTAAGACAAAAACAAATCATGG
>LpMenA2 Lindenbergia philippensis EJCM-2018390 (1KP)
TTGCACCTTAATTAGCTAGGTTTTTAACATGATGGCAGGAACCAGCTTGAATTTGGTAGAGAATGCAAGCAAGGAAAAAAATATTA
AAAGGTCAAAAAATGGGAGTACTAGTCCAAATGAAGAGAAAAATGAAGAAGAAATATCAAGAGCAACTTTGATATGGAGAGCTGCA
AAATTACCCATGTACACTGTTGCATTAATTCCTCTAACAGTGGGAACTTCAGCTGCATATTGGGAGTCAGGCTATTACTCTTTGGA
GCGTTATTTTATTTTATTGGCTTCTTTTGTTCTTGTCAATGTTTGGGTCAATTTAAGCAACGATGTTTATGATTTTGATACGGGAG
CTGATAAAAACAAAAAGGAGTCTGTTGTCAATATAGTTGGCAGTCGCACGGCCATCCATATTCTTTCATGGTTAATACTTGCGCTT
GGTTTTGCCGGCCTTACATGGGTTGGAGTCGAGGCTAAAAACCCGCGTGCTATACTGTTGTTGGCTTCAGCAGTCTTTTGTGGCTA
CATTTACCAGTGTCCACCGTTTCGTTTAAGCTACCATGGACTGGGAGACCCCTTATGCTTTGCGGCGTTTGGTCCTTTTTCCACCG
TTGCATTTTATTTGCTACAGAGTAGTTCAAGTGAGCTACCGATATCTAGCACGATAGTTTCCTCAGCAATTCTTGTTGGTTTTACA
TCAGCCTTGATCCTCTTTTGTAGTCATTTCCATCAGATAGAGGATGATAAAGCTGTTGGGAAAATTTCTCCTTTGGTGAGGCTTGG
CACTGAAAAGGGATCAAAAGTAGTGAAGATGGCTATTTTGGGGCTCTATTGGCTCGTGCTTGGTTTAGGACTCGCCCAAACTCTTC
CTTATGCTTGTATTGTCCTATGTGCTATGACACTACCCATGGGAAATTTAGTAGTTAGCTTTGTTCAAGAGAACCACAAAGATAAG
TCGAAAATCTTCATGGCTAAATACTACTGTGTGAGATTACACACTGTATTTGGAGCTGCTTTGGCTGTTGGATTAGCGGCATCAAG
AATCAATGGTGGGGATTATCTTCAGAGTCCATCAACGTATTTTGATCGTGCCAAGTTTGCGTATTTCTGAGTTTAATTATTAATTA
GCACTCATTTTCTTATATCTACCATCAGATACATCTTGAACTACATTTATTCTTGTTATAAAAGAG
>VtMenA1 Verbascum Thapsus XXYA-2074947 (1KP)
AAAAGCATTACCGTAGTTTTTACTCAAGGCATTGAAGTTTCAGCTGGTGACATAGACTATGGCAGCAGCTAATTTCTGTTCAATGA
GCCATGGCTATGGCGTCAAAAACCTCAACCAGTTCCTTCCAAGAAGATGTAATAATTACAGGGCTTATCAAGAATTATCTCCTCAA
GATGCCGTTCGAATGCCATTATGCAGTAAAACGTATTTCGACAAAACAATCATAAGGCGATTACATTCTAAGAAACCGCATCACAG
AATTTTGTTCAAGTGTGCAGCAGAGCTCATTGACACTCACGGTGAAGACGAAAAGGAAGAACATATCTCGAAAGCAACTCTGATAT
GGAGGGCCATCAAATTACCAATATATACTGTTGCATTGATTCCTATAACAGTAGGAAATGCAGCAGCTTATTTGCAGACAGGCCAG
TATTATGGAAAGCGTTATGTTATGCTATTGGTCTCTTCATTTCTCATCATAGCTTGGCTCAACTTAAGCAATGACGTTTACGATTT
CGATACTGGAGCAGATAAGAACAAGAAAGAATCGGTTGTTAACTTAATTGGAAGTCGAAAAGGAACCCGTGCTTTTGCATGGTCAC
TACTTGCACTTGGTTTCATGGGCCTTACAAGGGTGTCGATGGATGCTGGAAGTTTTTACCCTTTATTTCTACTTGCTTGTGCCATA
ATTTGTGGCTACATTTACCAGTGCCCGCCATTTCGGTTGAGTTACATGGGATTGGGAGAACCGTTATGCTTTGCAGCGTTTGGTCC
ATTTGCCACCACAGCCTTTTACTTTCTTCAGACAGGCACAAGGGATCTGTCGATATCTGCCACTGTCATTTCTTCATCGATCCTTG
TGGGTATTACAACATCCTTGATCCTCTTCTGCAGTCATTTTCATCAGATAGACGATGATAAGACTGTTGGAAAATTATCCCCTTTG
GTTAGGCTTGGAACTGAAACTGGCGCGAAAGTAGTGAAAATCGCTGTAATAACACTCTATTCGCTGCTATTTGTATTAGGACTCAG
CCAAACTCTTCCTTTCTCATCCATTGTACTCTGTGCTTTAACATTACCGATAGGGAACAAAATAGTTAGATTCGTTGAGGAGAATC
ACAAGGACAAAACGAAGATTTTCATGGCGAAATATTACTGTGTGAGATTACATACTGTATTCGGAGCTGCATTGGCTGCCGGGCTG
GTGGGGGCTAAAATGTTCGCCCGAAAAAAAATACCACATGCTATGATCATCTCATAACGGCGGCCCGAATACTC

>PaMenG2 Phelipanche aegyptiaca Pa.c176185_g1_i2.37080
TACACGACGCTCTTCCGATCTCAGGGGACCGAAAACCGAAAATGGCTACAGCTACACTTAGACGCCGAACAGGAACACAGACCGCA
ACCCATCAGGGCGCAGCTGAACGCCAGGAGCTCTTTAACCGCATTGCCCCGGTCTATGACAAATTGAATGACGTGTTTAGCCTGGG
CTTGCATAGATTATGGAAGAGGTGGTCTATCTCTTGGAGCGGTGCAAAAGAAGGTGACAAGGTGTTGGATGTGTGCTGCGGAAGTG
GGGATTTTAGTTTTCGGCTGGCTGAGAAAGTTGGGATCAATGGCAAGGTCATTGGTCTTGATTTCTCCAAGGAGCTATTACAGGTC
GCTGCATGTCGTCGGCGTGACCAATCAAGTTCAAAGCCGTGCTACAACAACATTGAGTTCATTGAAGGAGACGCAGTTGCTTTGCC
TTTTCCCGACTCTACTTTTGACGCCGCTACGATTGGTTACGGGTTAAGAAATGTCATTGATCGGAAAAAAGCTCTCGAGGAGATGG
TCCGAGTTCTTAAACCGGGCGCAAAATTATCTGTTCTTGACTTTAACAAAAGCACTCATTGGCTAAACATTAAAATTCAGGAATGG
ATGATTGATTATGTAGTGGCTCCAGTTGCAAGTTGGTATGGACTTGAAAGTGAGTACAGATACTTGAAGATTTCTGTCAAGGAATA

TCTCACAGGAAGTGAGTTGGAGAAGATGGCTTTGGAAGCTGGTTTTTCCGTTGCCAAGTTCCATTCGATTGCTGGAGGAGTCATGG
GAAATTTGGTCGCCACGCGCTAGAACATATTCCTCGTTCCGTTCTATTTCATCATTCTGGTGTTCGCTTTCTGCAATTTCTGAAAT
GCTTTGCGTTCGTGTTTGTTTTTGTTATCACATTATTATGATTTTCGATGATTGTGGCCCGATCCAGTCGATCTTGTTAAAATTTC
TATCAGAAATAAAATTTCTTTGAGCTTTTGTGATCCATTGTTTTTGTTATCAGAAATAAAATTTCTATGAATCTGTCTGTTGAAAT
GTTG
>ShMenG1 Striga hermonthica Sh.c17385_g1_i2.603
AAAAAATGGAAAATCTTGAGGGTAAATTAGACCGTTCACTTGCTCAGAACTGAAAAGGCTATGGCTACTCTTCACTTCACTCTCCC
GTCTACAACCGGCCGGCAGCGGCCGCCGGAATTCCGGTCCATCTTCAAACCGGCTCGGTGTGCAGCTGAGAGACAGGCCCTATTTG
ACCGAATTGCCCCTGTCTATGATAACTTGAATGATTTGTTGAGTTTAGGGGGCCACAGGGTATGGAAGAGGATGGCCGTTTCTTGG
ACTGGAGCAAAAGAAGGAGACACTGTGTTGGATGTGTGTTGTGGGAGTGGGGATTTGGCCTTTCTATTGTCTGAAAAAGTTGGAGT
CAAAGGCAAGGTTTTTGCTGTTGATTTTTCGAAGGAGCTATTACAGGTTGCTGCATCTCGACAGCTTAAGCGGTCAAAAGCGTGCT
ACAAAAACATCGAGTGGATTGGAGGAGATGCGGTCGATTTACCCTTCTCTGGCTCGTATTTTGATGCTGCTACCATTGGATATGGG
TTGAGAAATGTGGTAAATAGGAAAAAGGCCCTGGAGGAAATGAGTCGGGTACTAAAACCCGGTTCGAAACTATCTGTTCTTGATTT
CAACAAAAGCACTAACCCGTTAACGAGCTCAGTCCAGGATTGGATGATTGACTATGTAGTGGTGCCCGTTGCTAGTGGGTATGGCC
TAGCAAGTGATTATAAGTACCTGAAGAACTCAATCAAGGAATATCTCACGGGAGATGAGTTGGAGAAGGTAGCTTTAGAAGCGGGC
TTTTCCCGGGCCAGACATTATGAGATTTGTGGAGGGTTAATGGGTAATTTGGTGGCCACTCTCTAAGAATGTTCTTATTGTTCTTT
TAATAACGTTTTTAAGAGTCGTAAGTGTGATATGTTTGTTCTGAAATATAGTTTAGCTTTTTTTGTAACATTTAGAGTGCTTATAC
TCCCTGTTTCACGATATGAGGTTCTAGAATTTTTGCACATGGAATTTAGTCCAGAAATGGAGTTTATCATGATGTCGATAAAACTT
GATTTTAAAAAGCTTCGCTCATAATTTGATTCCAATAAACCGTATTAATAAGCAAGAAAACTGCATAATAAATTATCAAGAAATAT
AATTATTCTGTTTGCATATATGCCTATAGTTATATTGTCTGTAAGTATTCCTTTCGCTATACATATTCTTTTCTACAACTCTATTG
TACCTTTTATAAATAAAAAGCCGAACTTCCTACATCACCTAGGATAGAAATTGCTGTGGGACATATTAGCAATAGAATCACCATAG
CTGTACTCGAGGGGAGATACCGTTTTTGCCCTTGAGTCGTTAAGTTCCATTGTTATCTCATTCATGAAAGGAGGATTAGTCGGGCT
CGGTAATTCTTCTTTATCCACAAGCATTTGTAAAACCTTCGACATTGATGGCCTTAACGAGGGGTTTTCTTGCATGCACAAGAGCG
CCGCGTGGACTACACGCAACATCTCGTTTTTTGCATTGATATTGTTGTAGTTTTGCTGCATTAG
GTTCGGGTCGAG
>ShMenG2 Striga hermonthica Sh.c18886_g2_i1.0
AGATCAAAAGGCTCAAATGTTGGGTGGCCCTTAACAGATTTTCATCACAAATTGGATACATATGGCCACACTTAGACGCCGATCTG
AGCCACAGCCGCCGAGCCATGAAAGTGCAGCCGAGCGCCAGGAGCTTTTTAACCGCATAGCACCTGTCTATGATAAACTGAATGAC
TTGTTTAGCTTGGGATTGCATAGATTGTGGAAAAAGTGGTCTATTGCATGGAGCGGGGCAAAAGAAGGAGACAAGGTGCTGGACGT
GTGTTGTGGCAGTGGGGATTTGAGTTTTCTGTTGTCCCTCAAAGTCGGGATCGACGGCAAGGTCATTGCTCTGGATTTCTCAAAGG
AGCTATTAGAGATTGCGGCATCTCGCCAGCGCGAGTGGTCCAAGTCGAAACCGTGCTACGAAAACATCGAGTGGATTCATGGAGAT
GCAGTTGCTTTACCGTTCCCCGACTCGAGTTTTGATGCTGCCACCATCAGCTATGGGTTGAGAAATGTGGTGGATAGGAAAAAGGC
TCTCGAGGAGATGGTTCGGGTTTTGAAACCCGATGCCAAGTTATCCGTTCTTGACTTCAACAAAAGCACTAATCGGCTGACCTCTA
AAATCCAGGATTGGATGATCGACTATATAGTGGTTCCAGTGGCGACCTGGTATGGGCTTGCAAGCGAGTATAGATACTTGAAGAAT
TCAATCAAGGAGTACCTCACAGGGAAAGAGTTGGAGAAGTTAGCTTTGGAAGCAGGCTTCTCTGAGGCCAAGCACTATGAGATTGC
TTGTGGATCCATGGGGAATTTGGTCGCCACACGCTAGAAATTTGATTGTTCCACTTTTACTGTTTCTCTTGTGTTTGTTATATTCT
GCAATTTTCCAAATGCTTTCATTTTTGTCATTTTTTTTTTCAACCATTTAATGATTTCTGATGATCTTTTTGGTCTAAAGAACCAT
ATGCTAGAATCCCATATGCTAAAATTTCCTCTCCGGCCTCCGTACCTTTATACGGATGGACCAATTGGAATACTATTCATTCATAT
TTGTGTAATCG
>TvMenG1 Triphysaria versicolor Tv.c7280_g3_i7.0
AAATAATGTTGTTTACAAAGAACTGAAAAGAAAATGATTTCACTTCAATTCACTCTTCCCTCCATAACCAGCCGGCGAAGTTTACC
GGAATCCCGGCCCATTCTCAAACCTATCCGCTGCGCATCTGACCGTCAGGCGCTTTTTAACCGCATTGCCCCTGTCTATGATAACC
TGAATGATTTGCTTAGCTTAGGAGCACATAGAGTATGGAAAAGGATGGCTGTTTCTTGGAGCGGAGCTAAAGAAGGTGACACTGTG
TTAGATGTGTGTTGTGGGAGTGGAGATTTAGCTTTTCTCTTGTCTGAGAAAGTTGGAACCAATGGAAAGGTTTTTGCTCTCGATTT
CTCAAAGGAGCAATTATATATCGCGGGATCTCGACAAAGCAAACGATCGAAAAATTGCTACAAGAATATCGAGTGGATTGAAGGAG
ATGCGGTTGATTTACCATTCTCTGCCTCGTTTTTCGATGCTGCAACGATTGGTTATGGTTTAAGAAATGTATTAGATAGGAAAAAA
GCTCTTAACGAAATGTGTCGGGTTCTAAAACCGGGTTCGAAATTATCTGTTCTTGACTTCAATAAAAGCACTAATCCATTAACCTC
TTCAATTCAGGATTTGATGATCGACTATGTAGTTGTCCCCGTTGCTGACGGGTATGGCGTAGCCAGTGATTATAAGTATTTAAAGA
ACTCAATTCAAGAATATCTCACAGGGAACGAGTTGGAGAAGCTAGCTTTAGAAGCGGGCTTTTCTCGGGCCAAGCATTACGAGATT
AGTGGAGGGTTAATGGGTAATTTAGTCGCTACTCTTTAGAAATTTATTCATAGAAAAAAGAACAAAAATCGTGTCTTTATTACTTG
CTTCGTGTTTGAATAAATATAT
>TvMenG2 Triphysaria versicolor Tv.c6701_g1_i1.0
GCTTGTACGAAATCAAAGTGCTTAAACAGATCCAATGTCGACGACGCTTCTTCGCCGCCGATCGGAGTCAGAGCCGGCCAGCCATG
AGGGCGCAGCTGAGCGCCAGGAGCTTTTTAACCGCATTGCACCTGTCTATGATAAATTGAATGATGTGTTCAGCTTGGGTTTGCAT
AGATTATGGAAGAGGTGGTCTATTTCTTGGAGTGGGGGAAAAGAAGGAGACAATGTGTTAGATGTGTGCTGTGGAAGTGGGGATTT
GAGTTTTCTGTTTTCTCAGACAGTTGGAGTCAATGGCAAGGTTACTGCTCTGGATTTCTCGTCGGACCAACTGCAGGTGGCTAAAT
CTCGCCAGCACGAGCGATCAAAATCAAACCCGTGCTACAAGAACATTGAATGGATCGAAGGCGATGCGGTTGCTTTGCCATTTCCG
GACTCGACTTTTGACGCAGCTACGATTGGTTACGGGTTAAGAAATGTGTTGGATAGGAAAAAGGCTCTGGAAGAGATGGTTCGTGT
TCTTAAACCGGGTGCCAAATTATCTGTTCTTGACTTCAACAAAAACACTAGTCAAGTGATGTGTAAAATTCAGGATTGGATGATTG
ATTATGTAATTGTTCCGGTTGCAAGTTGGTATGGACTTGCAAGTGAGTACAGATACTTGAAGAACTCAATCAAGGGATATCTGACA
GGAAGTGAGTTGGAGAAGCTAGCTTTGGAAGCCGGTTTTTCTGAAGCCAAACACTATCCGATTGCCGGAGGAGCAATGGGAAATTT
GGTCGCCAAGAAATAGAAACGGGTTTTGTTGTTCTTGTTGTTTCAATAATTATCTGTTTGTTGTTGTTGTTGTTGTCGTTGTTTTA
ATAATAAATTGTAG

>AfMenG2 Aphyllon (syn. Orobanche) fasciculata PHOQ-2096404 (1KP)
TCATAGTTGACTCCTAAAAACAATGGAACTTGTGTAATATTAAAATAAACATTTACTCTCTTACAGCATAGATAAGATATACAAAC
ATTTATATATTGCTCGGCCTTTGGGACCGTACCAGAGACTGTAAATCGAAATGGCTACACTTAGACGCCGATCAGAAGCACAGCCT
TCAAGCCATGAGGCCGCAGCTGAGCGCCAGGAGCTTTTTAACAGCATTGCACCGGTCTATGATAAATTGAATGATTTGTTCAGCCT
GGGCTTGCATAGATTATGGAAGAGGTGGTCTATTTCTTGGAGCGGGGCAAAAGAAGGTGACAAGGTGTTGGATGTGTGTTGTGGAA
GTGGGGATTTGAGTTTTCTGCTGTCTGAGAAAGTTGGGATCAATGGCAAGGTCACGTCTCTAGATTTCTCAAAGGAGCAATTGAAG
ATCGCTGCATCTCGCCAGCGTGAGCGATCAAAGTCAAAGCCCTGCTACAACAACATTGAGTGGACCGAAGGAGATGCAGTTGCTTT
GCCGTTTTCCGACTCCACTTTTGATGCTGCTACCATTGGCTATGGGTTAAGAAATGTTATTGATAGGAAAAAAGCTCTTGAGGAGA
TGGTTCGGGTTCTAAAACCGGGTGCAAAATTATCTGTTCTTGACTTTAACAAAAGCACTAACTGGCTAAACTCCAAAATTCAGGAT
TGGATGATTGATTATGTAGTGGTTCCAGTTGCAAGTTGGTATGGACTTACAAGTGAGTACAGATACTTGAAAAACTCCATCAAGGA
ATATCTCACAGGAACTGAGTTGGAGAAGCTAGCTTTGGAAGCCGGTTTTTCCGTTGCCAAGCACCATGCGATTGCTGGAGGATCCA
TGGGGAATTTAGTCGCCACTCGCTAGAAATGTTTTCGCTCCATTCTATTGCAAGGACATGTTCCTCATTCTCTTTCTGCAATTTCC
GAAATGCTTTGTTTTTATTGTTGTTATCGTTGTCATGATTTCCGATGATTATGGCTGATCCGATGAACCTTGTTAAACTATCGGAA
TTTAGTCGGTTTGAGTTTATGTAATAATAGTCAGAGTAATGGTTTATCCATATGAAGTCTCTTTGTTGAACTGTTAACTTTTGCAT
CTTTGAGTGCTCAATATGTATTGAACTTTGTGTGTCTGTTGTATCTTATATTCACTTGTTCAATTAGTCCCGGCCTAGGTGCTGGC
CTGTCCGTAGCCTC
>CaMenG2 Conopholis Americana FAMO-2024401 (1KP)
TTGAGACTGAAAATCGAATGGCCGCTACACTTAGACGCCGATCAGAAGCACCTGCACAGCCCGCTGCCGCAAGCCATGAGGGCGCA
GCTCAGCGCCAGGAGCTTTTTAACCGTATTGCACCTGTCTATGATAAATTGAATGATTTGTTTAGCCTCGGGTTGCATAGATTATG
GAAAAGGTGGTCTATTTCTTGGAGCGGGGCAAAAGAAGGAGACAAGGTGTTGGATGTGTGTTGTGGAAGTGGGGATCTGAGTTTTC
TGATGTCTGAGAAAGTTGGAAGTGGCAAGGTTAATGCTCTAGATTTCTCAAAGGAGCAATTACACATTGCTGCATCTCGCCAGCAT
GAGCGATTGAATTCAAAGCCGTGCTACAAGAACATTGAGTGGTTTGAAGGAGATGCAGTTGCTTTGCCATTTTCGGACACAATTTT
TGACGCAGCTACGATTGGGTATGGGTTAAGAAATGTGGTGGATAAGAAAAAAGCTATGGAGGAGATGGTCCGAGTTTTGAAACCGG
GTGCCAAATTATCTGTTCTTGACTTTAACAAAAGCAACAATTGGCTAAACTCTAAAATTCAGGATTGGATGATCGATTATGTAGTT
GTTCCGGTTGCAAGTTGGTACGGACTTGCAAATGAGTACAGATACTTGAAGAACTCAATCAAGGAATATCTCACAGGAAAGGAGTT
GGAGAAGCTAGCTTTGGAAGCAGGTTTTTCTGAAGCCAAGCACTATGAGATTGCTGGAGGATACATGGGAAATTTGGTCGCCATTC
GCTAGAAATTTATACTTTTGTTCCCATTTTGCTTCCCTGATTACAATGCAAGGAGGACATGTTCTTCATTCCTTCCTTCCTTCGTA
TGC
>RgMenG1 Rehmannia glutinosa OWAS-2002674 + OWAS-2025114 + OWAS-2044184 (1KP)
GTGGTTTTTACCAAGAACTCAAAAAATAGAATGGCTACACTTCAATTTACTCTTCCCTCCATAACCGGCGGGCGACGTCTACCGGA
ATCCCGGTCCACTATTAGACCGGTTCGGTGCGCAGCTGAACGGCAGGCGCTTTTTAACCGCATTGCCCCTGTCTATGATAACTTAA
ATGATTTGTTTAGCTTAGGAAGCCATAGAATATGGAAGAGGATGGCTGTTTCTTGGAGCGGTGCAAAAGAAGGAGATACCGTGTTG
GATGTGTGTTGTGGAAGTGGGGATTTGGCTTTTTTGTTGTCTGAGAAAGTTGGAATCAATGGCAAGGTGATTGCTCTTGACTTCTC
AAAGGAGCAATTGACAATTGCTGCATCTCGCCAGCGCAAGCGATCGAGGGCGTTCTACAAGAATATTGAGTGGATTGAAGGAGATG
CAGTTGATTTACCATTCTCCGACTCCTCTTTCGATGCTGCAACCATTGGCTATGGGCTAAGAAATGTAGTAGATAGGAAAAAGGCC
CTGGAGGAGATGTGTCAAGTTCTGAAACCTGGTTCAAAAGTATCTGTTCTTGACTTGAACAAAAGCACTAACCCATTAACCTCTTC
AATTCAGGATTGGATRATTGACTATGCAGTGGTTCCTATCGCCAACGGGTATGGCGTAGCAAGTGATTATCAGTACTTGAAGAACT
CTATCAAAGAATATCTCACAGGAAATGAATTGGAGAAGCTAGCTTTAGAAGCAGGTTTTTCTAAGGCCAAACATTATGAGATTGGT
GCGGGGTTCATGGGAAATTTGGTAGCCACCCTTTAAGGAATGTTTTCATGCAGTCTCCTTCTTCTCAAGGCAGGTGCATTTTCGTG
TGCTCGCTTTCTGCAACTTCTAGAAGGTTCTATCTCTCTTTCTCTTTCTCCATCTGTATATGCTGA
>RgMenG2 Rehmannia glutinosa OWAS-2072104 (1KP)
GTATTTCTAATACATATTACCCAGATTCTTACATCTTAGATTAGTTTCTACACTTATATATTGCTACTCTAGTAGTGTTGAAAGAA
ATCGAGGGACTTTAGCAGAGAGTGAAAATCGAAATCGAATGGCAACACTTAGACGTCGATCGGAACCACAGACCGTAACCCATGAG
AGCGCAACTGAGCGCCAGGAGCTTTTTAACCGCATTGCTCCTGTCTATGATAAATTGAATGATTTGTTTAGCCTGGGGCTTCATAG
ATTATGGAAGAGGTGGACTATTTCTTGGAGCGAGGCAAAAGAAGGAGACAAAGTGTTGGATGCGTGTTGTGGAAGTGGGGATTTGA
GTTTTCGGTTATCTGAGAAAGTTGGAATCCATGGCAAGGTGATAGCTCTAGATTTCTCAAAGGAGCTATTAGAGATAGCTGCATCT
CGCCAGCGTGAGCATGAGCGATCAAAGCCATGCTACAAGAACATTGAGTGGATTGAAGGAGATGCAGTTGCTTTGCCATTTTCTGA
CTCCACTTTTGACGCTGCTACCATCGGCTATGGGCTAAGAAATATTGTTGATAGAAAAAAAGCTCTGGAGGAGATGCATCGAGTTT
TAAAACCGGGTGCAAAATTATCTGTTCTCGACTTTAACAAAAGCACTAATTGGCTAAACATTAAAATTCAGGAATGGATGATTGAT
TATATAGTGGTTCCTGTTGCAAGTTGGTATGGACTTGCAAGTGAGTACAGATATTTGAAGAACTCAATCAAGGAATATCTGACAGG
ACCTGAGTTGGAGAAGCTAGCTTTGGAAGCAGGGTTTTCTGAAGCTAAATTCTATGAGATTTCCAATGGATCCATGGGAATTTTGG
TAGCCAAGCGCTAGACACATTTTCTGTCCACATTCTGCTTCCCCGACAGGTTTCCGTTGTTGTTGTTGTTGTTGTTGTTGTTGTTT
ATGATGGTTATGTTGATCCAATGTAGCTTGCTAAAG
>LpMenG1 Lindenbergia_philippensis EJCM-2011304 (1KP)
AGCCATCTAAATTGTTTTCTTTGCACGGTCGATTTTCTGTATAAAACTCGAAATACAGTGGAGTTCTTCTAGAACTGAATTGAAAA
TGGCTACACTTCAATTCACTCTTCCCTCCATAACCGGCGGCCGACGTCAACCGGAATCCCGTTCCATTCTTAGACCGGTTCGCTGC
GCAGCTGAACGCCAGGCGCTTTTTAATCGCATTGCCCCTGTCTATGATAACTTGAATGATTTGTTAAGTTTAGGGGCCCATAGAAT
ATGGAAGAGGATGGCTGTCTCTTGGAGCGGAGCCAAAGAAGGAGATACTGTGTTGGATGTGTGTTGTGGAAGTGGGGATTTAGCTT
TTCTGTTGTGCGAGAAAGTTGGGATCGATGGCAAGGTGATTGCTCTTGATTTCTCAAAGGAGCAATTACAGATTGCTGCATCTCGT
CAGCTCGCGCGATCAAAGGCGTGTTACAAGAATATTGAGTGGATTGAAGGAGATGCAGTTGATTTACCATTTTTCGACTCGTCTTT
TGATGCTGCAACTATTGGCTATGGGTTAAGAAATGTGGTAGATAGGAAAAAAGCTCTAGAGGAAATGTGTCGAGTTTTAAAACCGG
GTTCGAAATTATCTGTTCTTGACTTCAACAAAAGCACTAGCCCATTAACCTCTTCGATTCAGGATTGGATGATTGATAATGTAGTG
GTTCCTGTTGCCAGTGGATATGGCGTAGCAAGTGATTATCAGTACTTGAAGAACTCAATCAAGGAATATCTCACAGGGAATGAGTT

GGAAAACTTAGCTTTAGAAGCAGGCTTTTCTGAGGCTACCCATTATGAGATTGGTGGCGGTTTAATGGGAAATTTAGTAGCCACGC
TTTAAGTGTA
>LpMenG2 Lindenbergia_philippensis EJCM-2011303 (1KP)
CGTTGTAAAAAAATCGAGGGGCTTTAGAAGAGACTGGAAAATCGAAAATGGCTACACTACGACGTCGTTCCGAGCCACCGGCCGTG
ACCCATGACAGCGCTGATGAGCGCCAGGAGCTTTTTAACCGTATTGCTCCTGTTTATGATAAATTGAATGATTTGTTCAGCCTGGG
TTTGCATAGACTATGGAAGAGGCGGGTTATTTCTTGGAGTGGGGCAAAAGAAGGAGACAATGTACTGGATGTGTGCTGTGGAAGTG
GGGACTTGAGTTTTCTGTTGTCTGAGAAAGTTGGAGTCAATGGCAAGGTTATTGCTCTAGATTTCTCAAAGGAGCAATTACAGATT
GCTGCATCTCGTCAGCTCGCGCGATCAAAGGCGTGTTACAAGAATATTGAGTGGATTGAAGGAGATGCAGTTGATTTACCATTTTT
CGACTCGTCTTTTGATGCTGCAACTATTGGCTATGGGTTAAGAAATGTGGTAGATAGGAAAAAAGCTCTAGAGGAAATGTGTCGAG
TTTTAAAACCGGGTTCGAAATTATCTGTTCTTGACTTCAACAAAAGCACTAGCCCATTAACCTCTTCGATTCAGGATTGGATGATT
GATAATGTAGTGGTTCCTGTTGCCAGTGGATATGGCGTAGCAAGTGATTATCAGTACTTGAAGAACTCAATCAAGGAATATCTCAC
AGGGAATGAGTTGGAAAACTTAGCTTTAGAAGCAGGCTTTTCTGAGGCTACCCATTATGAGATTGGTGGCGGTTTAATGGGAAATT
TAGTAGCCACGCTTTAAGTGTA
>VtMenG1 Verbascum Thapsus XXYA-2018913 (1KP)
CCCTCCAATACAAAATTACTTCGTCCAAGTCCACTTCCCCCAAGTATGGGCATAAAAGCCAATGGAATTGGATGGGTTTTAGCAAA
ACCGGAAAAATCAAAATGGCCACACTCCAATTCATTCCTCCCTCCATCACCGGCCGGTCCAGTTCCCGGTCAATTCGACGTCCGGT
TCAGTGTTCGGCTGAACGGCAGGCGCTTTTTAACCGCATTGCTCCGATCTATGATAACCTGAATGATTTGTTGAGCTTGGGGAGAC
ATAGAGTATGGAAGAGGATGAATGTTTCTTGGAGCGGAGCAAAAGAAGGAGATACTGTGTTGGATGTGTGTTGTGGAAGTGGGGAT
TTGGCTTTTCTCTTGTCCGAGAAAGTTGGAACGAATGGCAAGGTGATTGCTCTTGATTTCTCGAGCGAGCAATTACAAATCGCTGC
CTCTCGCCAGCGTGAACGAACAAAGGCCTGTTACAAGAACATTGAGTGGATTGAAGGAGATGCAGTTGATTTACCATTCTCTGACT
CGTCTTTTGATGCTGCTACAATTGGCTATGGATTAAGAAATGTAGTGGATCGAAAAAAAGCTCTTGAGCAGATGTTTCGTGTTTTA
AGACCTGGTTCAAAGTTATCCATTCTTGACTTCAACAAAAGCAGTAGCCCATTAACCTCTACATTACAGGATTGGATGATCGACTT
TGTAGTAGTTCCTGTTGCTAGTGGATATGGCCTTGCGAGTGAGTACCAATACTTAAAGAACTCAATCAAGGAGTATTTGACAGGAA
AAGAGTTGGAGAAGCTAGCTTTAGAAGCGGGATTTTGTGATGCCAAATTTTTCGAGATTGAGGCTGGATTAATGGGAAATTTGGTA
GCCACCCGCTAAAAATGATTTTTGATCCAATTTGCTATATACAGTCTCAAATGCCATCTTTCAGCGAGTCGGGTACTTTTTTTACA
GTGTTTTTTCCCCTTCACATTGTAGAATAATAAACTTATATCATTTGGTTGCTCATTGTATGATATGATGAAATAAAAATGTTGAA
ATTTGTATTTATTAAACTCTGTCTCCCTTTCAATACATGTAATAAATGCATGAACTTAGAGCATGGATATAAAAAATGTATTTGGT
TCCCCCAC
>VtMenG2 Verbascum Thapsus XXYA-2014945 (1KP)
GACGGCATCTCCTTAATTAACTATTTCCGGATTATATATTATTGCTCGCCTCTGGAACAGATGAGATGAGTTTTAGTGCGTATAGA
ATGGCATCAATACGACGTCGTTCGGAGTCACAGCCCGCCGGAAAATTCGCCGAGGGCGCAGCTGAGCGCCAGGAGCTTTTTAACCG
CATTGCTCCTGTTTATGATAAATTAAATGATGTGTTTAGTCTGGGGATGCATCGATTATGGAAGAATTGGTCTATTTCTTGGAGCG
GGGCAAAAGAAGGAAACAGAGTGTTGGATGTGTGTTGTGGAAGTGGGGATTTGAGTTTTCGATTTTCTGAGAAAGTGGGCATCAAT
GGCAAGGTGATTGGTTTAGATTACTCAAAGGAGCTGCTACAAATCGCTGCAACTCGCCAACATAAGCTCGTGCAATCGAAGCCAAG
CTACAAGAACATTGAGTGGATTGAAGGAGATGCAACTAAGTTACCATTCCCTAATTCCTCTTTCGATGCTGCTACAATTGGCTACG
GTTTAAGAAATATTGTCGATAGGAAAAACGCTCTGGAGGAAATGGTTCGAGTTCTTAAACCGGGCTCCAAATTATCTGTTCTTGAC
TTCAATAAAAGCACTAGCAAATCAATGTGTAAATTTCAGGAATTGGTGCTCGATTATATAGTGGTTCCTATTGCAAGTTGGTATGG
ACTCAGGGATGAGTATCATTACTTGAACAAGTCGATCAAAGAGTATATGACAGGAAACGAGTTGGAGAAATTTGCTTTAGAAGCGG
GATTTTCTGAAGCCAAGTATTATGAGATTGCCGGAGGAGCCATGGGAAATTTGGTAGCAACGCGCTAGAAAATTTGGCGACTTTTA
GTCGATTCTT

>PaQR2 Phelipanche aegyptiaca Pa.c21330_g1_i1.5664
AGAGAGACGACCCGTACCCTTCCAATGGCGACCACACTTTCTATTGTTTACTATTCGACATACGGGCATGTTGAGAAACTTGCGCA
AGAGATCAAGAAAGGAGCAGAGTCTGTTGAAGGAGTTGAGGCTAAACTCTGGCAGGTTCCAGAAACTCTGTCCGATGAGATTCTTG
GGAAGATGGGTGCCCCGCCAAAGAGTAGTGAAGTGCCTGTAATAACCCCAGACGAACTTGCCGAGGCTGATGGTTTTATATTTGGT
TTTCCAACTAGATTTGGAATGATGGCTTCTCAATTTAAAGCTTTCTTTGATTCAACTGGAGGGTTATGGAGGACTCAGGCATTAGC
CGGCAAGCCTGCTGGCATTTTTTACAGTACTGGATCTCAAGGCGGGGGGCAAGAAACTACAGCCTTAACAGCCATTACACAGCTGA
CTCATCACGGTATGATATTTGTCCCAATTGGTTACACATTTGGAGCTGGAATGTTTGAGATGGAGAAAATCAAAGGTGGGAGCCCT
TATGGTGCTGGGACATATGCCGGAGATGGTTCCCGACAACCTTCCGACATTGAACTTGCACAAGCTTTTCATCAGGGAAAGTACAT
TGCCGCCATCACCAAGAAGCTCAAAAAATCTGTCTAATTTCACACTTCCTTATCAACATCTCCAATATTAATTCCATCCTACGAAT
TAAATATGCTGTTTTTCCTGTTTCGGTTATAAGTTTGCTTTGTTATGTCTTCGTCAAATTAAATTGTTATGTTTTTCAGTCGGAAT
AAATTGGTTTTCACGTACTTTATTTTATTTTATTGGGTTATGTCGATGTTTTTCACATATGCCGCAAACTTATCATAAAAATCAAG
AATCATACATCATCATTACTTTTCCAAAAAA
>ShQR2 Striga hermonthica Sh.c13828_g1_i1.4428
ACTGAATCTTCTTCTTCCATAACCATAACAGTATTGTCTATAAATAGCAGCCTTAATTCTCATTTACTTTCACAACACTACTTCAC
ACACCAAATCAAACTAAAAGAAACACACTTCATTATTACAATGGCTGCTAAAGTATACATTGTTTACTATTCGACCTACGGGCATG
TCGAGAGACTCGCGCAAGAGATCAAGAAAGGAGTCGAATCCATACCAGAAGTTGAAGCTAAACTTTGGCAGGTCCCGGAAACTCTC
ACGAACGATATCCTCGCGAAAATGGGGGCCCCACCAAAGAACAACGACGTGCCGGTTATTAGCCCGAACGAACTTGTGGATGCCGA
TGGCATCATATTCGGTTTTCCGACAAGATTCGGGATGATGGCCGCTCAATTCAAAGCATTTTTCGACTCGACCGGTGGCTTATGGA
GGACTCAAGCACTTGCCGGAAAACCTGCCGGAATCTTTTATAGCACCGGATCTCAAGGCGGTGGCCAAGAAACCACACCATTAACA
GCCATAACACAACTTACTCATCACGGCATGATATTCGTGCCTATTGGCTACACGTTTGGAGCCGGGATGTTTGAAATGGGGGAAAT
TAAAGGCGGGAGTCCTTATGGTGCCGGGACATATGCAGGGGATGGGTCGAGACAACCCTCAGGAATCGAACTCGAGCAAGCTTTTC
ACCAGGGAAAACACATTGCAGCCATCACTAAGAAGCTCAAACAAACTACTTAATGTGGGTCACGAATAGTTTCCCGATTCATCAAA

TAAATTTGTATGCTTTTTTTTTTTTTTGTCGGAATAAATTGATCTTAATTGGTGTACTTCGTTTAGATATCGATTACGAATTGCTTT
AAGTTCATTAGATATACAATTTGCTACAGAAAATTTTACTCAGTTGTAGCTATGAATTGATGTTATATTGTCTATGTTTATTTTTC
TTTGCGTATTGAAATTATGGCCTTTAATCGTGTCTAAAATTATATTTTGATTTGAAGAAAAAAATAAAAGTAGTCAAACTCGGGCA
ATTGCATATTCAAGCTTGTGTAATTTCGTCTCTATAGCTTTCTTGCAGCAAATTTTTT
>TvQR2 Triphysaria versicolor Tv.c6809_g1_i1.4592
CTTCCCAAGAACCTCATCCACCAAGAAAATCAAACTAAACCAAAATACAGAGCTAATTAAACAGTAACCCTTTAATATTCCAATGG
CAACCAAAGTCTACATCGTTTACTATTCGACATACGGACATGTTGAGCGGCTAGCGCAAGAGATCAAGAAAGGAGCTGAGTCTGTC
GGAAATGTTGAGGTTAAACTATGGCAGGTTCCTGAAATTCTGTCGGATGAGGTTCTTGGGAAGATGTGGGCCCCACCAAAGAGCGA
TGTGCCGGTGATCACACCCGATGAGCTCGTCGAGGCTGATGGTATCATATTTGGTTTCCCAACGAGGTTTGGAATGATGGCCGCTC
AATTTAAAGCCTTCTTCGATTCAACCGGAGGTTTATGGAAGACTCAAGCACTTGCCGGCAAGCCGGCTGGCATCTTTTTCAGCACT
GGAACTCAAGGCGGCGGTCAAGAAACTACCGCATTAACAGCCATTACACAGTTGACACATCATGGTATGATCTATGTCCCCATTGG
CTATACATTTGGAGCTGACATGTTTAATATGGAGAAGATCAAGGGTGGTAGCCCATATGGCGCTGGCACGTTCGCCGGAGCAGATG
GATCTAGACAGCCTTCGGACATCGAACTCAAACAAGCTTTTCACCAGGGAATGTACATCGCCGGCATCACAAAGAAGATCAAGCAA
ACATCGGCCTAATTTCGACCATAAATTTCATTCTACGAATAATAAACATATGTTGTTTATTATATACTTGATTCCTGTTTTATTTT
ATGATTTCCAGGCTTGATGTCTTCAACAAATAAATGTTACACTTTTTATTCGGAATAAAATTGATGTTCACATAAAAAAAAAAAAA
AAAAAAAACAATTACCACTAACTCCGAAAAATAAATAAAATAGTAACGACAAACAGTTGACCCCAGCGGCCCAGCCCATCAAAATT
GATCACAAAATCTCATTCTCATGAAGAATGATAATCAAGCCCAAGCTCCATTTTCCATAAGCCGTCTTCGCAAACAGGCTCATCAC
TATGGCTCGGCTTAA

>PaNDC1 Phelipanche aegyptiaca Pa.c24070_g1_i2.305
GTTTAAGTTGACTTGTGAGAGCTCGTCCTTTGTGAGACACTCGTGGAGCCAAGACGGCCACACACCCATACCGCCCAGCTCCGCCA
CCTTCGTCCCATTCTACCGTCGATCATAGATGGCACACGCAGTTGCATCTCTCACACCATCATCACTGCTCAGCTGTACGCTTTCG
TTTACCCCAGTATTTGGCGAGAAATCCCAGTTGCGGAATTTATTCCAGCAGCATTCGCTTAAATTGTGGACCAATTCGGGGTTCTA
TTCCAGAAGGCGTGGAGTCCCTGTTGTTGGTTCGAGCTCGAGCAGAGGGTACGGCGTCGTTTCTGCTGTTTCTGAAAGTGAGTCTC
AGCGTCCCAGTTACGTTTGGCCTGATAACGAGAAGAGACCAAGAGTGTGCATACTTGGTGGTGGTTTCGGAGGATTGTCCACTGCA
CTGAGATTGGAATCACTTGATTGGCCGGATAGAAAGAAACCACAGGTGGTTCTTGTTGATCAATCCGAGCACTTTGTCTTCAAGCC
ACTATTATATGAACTTCTCTCTGGAGAAGTAGATGAATGGGAAATAGCTCCTCGTTTTTCAGACTTGCTGTCCAGCACTGCTGTGA
AGTTTTTGAAAGACAGGGTTAAATCTTTACATCCCTCTTATCATTACGGACTGGTTGGGGCTTCCATAACTCATTCTGCTGGAGTC
GTGCATCTTGAAAGTGGTCTCCGTATTGAATATGACTGGTTGGTACTTGCCCTCGGAGCTGAAGCCGAACTTGATGTTGCGCCAGG
AGCAATAGAATATGCATTACCATTTTCCACTCTTGAGGATGCTCGTAGAGTCGATGAGAAGCTAAAAACACTTGAGCGGCAATTCT
TTGGTAAAAACTCTCCAATTCGTGTTGCCGTTGTAGGATGTGGTTACTCTGGAGTTGAATTGGCTGCCACAATATCAGAAAGACTA
CAAGCTAGGGGAGTTGTACAAGCAATCAATGTGGGCAAAACAATCTTGTCAAATGCTCCACCTGGCAACAGAGAATCTGCAATGAA
AGTTCTCTCATCCAGGAATGTTCAGCTTTTATTGGGTTACTCTGTTCGCTGCATAAGAAGAAATGTCGAGTATGCGGTTTCATCGG
AACCTACCAATGTAGAAGCTGATCATGATAAAGTGGAAGCTCATAATCAGAAAAGACTTGTATTGGAGTTGCAGCCTGCTGAAAGG
GGCATGCAGAATGAAGTTGTTGAAGCAGATTTAGTTTTATGGACTGTTGGGTCTAAACCTGTACTTCCTGATCTTGAACCCAGTGA
TGAACCCATTAAACTTCCTTTAAATGGTAGGGGGCAAGTAGAAACCGATGAAACTCTTCGCGTTAAGGGTCACCCACGTATATTTG
CAGTTGGGGACTCTTCTGCTGTGAGGGATAGACAAGGTAAATTGCTTCCAGGCACCGCACAGGTAGCATTGCAACAGGCAGATTCC
GCAGGCTGGAATTTATGGGCCGCAATTAATGGCCGACCGCTATTACCATTTAGGTTTCAGAATTTAGGTGAGATGATGACTCTTGG
GAGATATGATGCTGCTATTACACCAAGTTTCATCAAGGGTCTGACATTGGAGGGTCGAGCTGGTCACACTGCAAGGAAAATAGCCT
ACTTAATCCGACTACCAACAGAAGAGCACCGGTTAAAAGTAGGGATCAGTTGGTTAACGAAGACGGCTTTAGAGTCTACTGCGTTA
CTGCAGAATACTGTTACCAGAATGCTTTCGGGGAAGTAGAAGACGTTAGTATATCATGTGATTAAATTACTACCAAATTATCTTAC
ATGACAATATTATTTTTGAACTATCTTACATGCAATATTTTAAGAACAATAAGAAAATACCTGAAGCCTTCTGGCAGGTCTTTGAA
TTACATTGGATTGAGGTCTGTGTTTCTTACCATGTTATCTAATAGACCATCAATGCGAGA
>ShNDC1 Striga hermonthica Sh.c14874_g1_i1.8637
CGCTCACCTGCTCGCTCTCACTTGTGGAGCCACGACAGCGTAGTAATAGTATACCCCCACTTCCCGCCGGCAATTCCACACTCTTA
CACCCTCCACCGTAGATGGCGCTCGCTGTGTCTTCTATAACATCACTAACATTAATCCAGAGTGAGCGGTTCCGGCCGGTGAATCC
ATTCCGTGTTCTTGGTTCGAGTAGGTTATGGACCTACTCGATGTTCTTCTCAACCTCCGCCAAAAGTCCTCCACTCCGTGTTCTTG
GTTCGAGCTCCGGTGGAGGTTTTGGCGTCGTTTCTGATGCAGAATTTCAGCCTTCCAGTTACGTTTGGCCTGATAACAGGAAGAGA
CCAAGAGTGTGCATACTAGGTGGTGGTTTCGGAGGGCTGTACACTGCATTGAGATTGGAATCACTTGATTGGCCAGATGGAAAAAA
ACCACAGGTGGTTCTTGTTGATCGATCTGAGCATTTTGTTTTCAAGCCGCTACTGTATGAACTCTTATGTGGAGAAGTAGATGAAT
GGGAGATAGCCCCTCGTTTCTCAGACTTGCTGTCAAGCACTGGTGTGCAGTTTTTGAAGGACAGCGTGCAATATTTACTTCCCTTT
GATCAATATGGTATGGATGGGGATGCGTTAACTCATTCCGCTGGAGTAGTGCATCTTGAAAGTGGTCTCCTTATTGAATATGATTG
GTTGGTACTTGCTCTTGGAGCTGAAGCTAAACTTGATGTTGTGCCGGGTGCAACAGAATATGCATTGCCGTTTACCACTCTTGAGG
ATGCTCGTAGGGTCAATGAGAAGCTAAGAACACTTGAGCGTGAATTCTTTTGTAAGGACTCTCCAATTAGAGTTGCAGTCGTTGGA
TGTGGTTACTCTGGAATTGAATTGGCTGCCACAATATCAGAAAGACTCCAAACACGGGGAGTTGTACAAGCAGTCAATGTTGAGAA
AACCATTTTGTCGAATGCTACTCCTGGCAATCGGGAATCTGCATTAAAAGTTCTCAAGTCCAGGAGTGTTCAGCTTTTGCTGGGTT
ATTTTGTCTGTTCTATAAAAAGAGTTGCACAGTGTGACAATAAAGTAGGAGCACAAAACCATGAAAGACTTAAATTGGAGTTGCAG
CCTGCTGAAAGGGGTGTGCCTAGCCAAGATGTAGAAGCAGATTTAGTTTTATGGACTGTCGGTTCAAAACCTGTGCTTCCTCAGCA
CAAACCCGGTGATAAACCCATATCACTTCCTCTAAACAGTAGGGGTCAAGCTGATACTGATGAAACTCTTCGTGTTGAGGGTCACC
CGCGTATATTTGCTATTGGCGACTCTTCTGCTATGAAAGATGGTCGAGGAAATTTGCTTCCAAGCACTGCCCAGGTAGCATTTCAA
CAGGCTGATTTTGCCGGCTGGAATCTGTGGGCCGCAATAAATGGTCGACCTCTGTTACCATTTAGGTTTCAGAATTTAGGTGAGAT
GATGACGCTTGGGAGGTATGATGCTTCTGTTTCACCAAGCTTCACGGAGGGCCTGACATTGGAGGGTCCAATTGGTCACACTGCGA
GAAAAATAGCATACTTAAGCCGACTGCCAACAGACGAGCACCGATTAAAAGTAGGGATCAGTTGGCTAACAAAGGCTGCCATTGAA

TCAACTGCATTACTGCTGGATAGTATTACCAAAATGCCTTTCGAATTGTAGAGGACGGTGTAGTATCATATGCTGTGCTGCTCTCT
CTCTCTCTCTCTCTCT
>TvNDC1 Triphysaria versicolor Tv.c5468_g1_i1.261
GAAGCAAGGGCCATATATTTTTATCAGCCAACTGTTAGTAAAGCTGGTTCTTTCTCGACCACTCGTGGAGCCAAGACGGCCGCGTA
TACCATTTCGCCCCCGTCTCGTACCGTCGATCATAGATGGCGCTCGCAGTTTCCTCTCTCACGTCATTAACACTACGCAGCCGAGA
GCAATTCCAGTGCCATTCGCGTAAGTTTTGGACCAGATCGGGGTTCTTTTCTAGCTCTTCCTCTAGAAGAAGAGGAGCCTGTGTTG
TTGGTTCGAGCTCGAGCAGAGGATACGGCGTCGTTTCTGCTATTTCTGAAAGTGAATCTCAGCGTCCGAGCTACGTTTGGCCTGAC
ATAAAGAAGAGACCAAGAGTGTGCATACTAGGTGGTGGTTTCGGAGGGTTGTACACTGCACTGAGATTGGAATCACTTGATTGGCC
AGATGGAAAGAAACCACAGGTGGTTCTTGTTGACCAATCTGAGAACTTTGTCTTCAAACCACTACTATATGAACTCCTCTCTGGAG
AAGTAGATGAATGGGAAATAGCTCCTCGTTTCTCAGACTTGCTGTCAAGCACCAATGTGCAGTTCTTGAAAGACAGGGTGCGATCT
TTACATCCGTCTGATCATTATGGAATGGATGGGGCTGCTGTAACTAATTCTGCCGGGGTCGTGCATCTCGAAAGTGGTCTCCTTAT
CGAATATGATTGGTTGGTACTTGCCCTTGGAGCTGAAGCCAAACTAGATGTTGTGCCAGGTGCTATAGAATATGCATTGCCTTTTT
CCACCCTTGAGGATGCTCTTAGAGTCAACGAGAAGCTAAAAGCACTCGAGCGGGATTTCTTCGGTAAGGACTCTCCAATTCGTGTT
GCCATTGTAGGATGTGGTTACTCCGGAGTTGAATTGGCTGCCACAGTATCAGAAAGGCTGCAAGATCAGGGAGTTGTACAAGCAAT
CAATGTTGATAAAACAATTTTGTCAAATGCTCCACCTGGCAACAGGGAATCTGCAGTAAAAGTTCTCTCGTCTAGGAATGTTCAGC
TTTTGTTGGGTTACATTGTCCGCTGTATAAGAAAAAATGTTGAAGATCACAATCACGAAAGACTTGTACTGGAGTTGCAGCCTACC
GAAAGGGGAATACAAAGCCAAGAAGTCGAAGCGGATTTAGTTTTATGGACCGTTGGGTCTAAACCTCTAATTCCTGAGCTAGGATC
GAGTGATAAACCTGTTTATCTTCCTTTAAACGGTAGGGGACAAGCTGAAACTGATGAAACTCTTCGAGTTAAAGGCCACCCACGAA
TATTTGCAGTTGGTGACTCGTGTGCTGTGAGGGACGGAAAGGGTAAATTGCTTCCAGGCACCGCTCAGGTAGCATTTCAGCAGGCA
GATTTTGCAGGCTGGAATCTGTGGGCTGCAATTAATGGCCGGCCTCTATTGCCATTTAGGTTTCAGAATTTAGGCGAGATGATGAC
TCTTGGGAGATATGACGCTGCTATTTCACCGAGTTTCATCGAGGGCCTCACATTGGATGGCCCAATTGGTCACACTGCCAGGAAAA
TAGCCTATTTAATCCGACTACCAACAGACGAACACCGGCTAAAAGTAGGGATAAGTTGGCTAGCCAAGACTGCTTTAGAGTCTACT
GCATTACTGCAGGATACGGTTACCAGGGTGCTTTCCGGACAGTAGAAAGACGTATATTAATTATCATGGATTCTAGATACAAATTG
GCGCAGAGCATTGTAAAAGACAATTACAGTTTACAATGTATGCATAAATGTAATTTTGATGAAATAACTATATATGGGGTGCCCAA
TATCTGAATATTTAACATAATTATATTTCAAGAAAAGATTCTTTTTTAACATGTGGTAAAATTATATTATTGATTGTTATGTAGAT
TTTATGACATTGTGTTGCATATATTTC

>PaPRX1 Phelipanche aegyptiaca Pa.c19539_g1_i2.433
ACACAAATTATTTCAATATGGACAACACAAAATCCATCAACTCTCTTCTCACTCTAACAATCTCATCACTCATCCTCCTCTCGCTC
TCCATCACACCATCTCATGCACAACTATCTCCCACGTTCTACGACAGTACATGTCCCAATGCACTCAGTACCATCCGCACTTCCAT
CCGACGAGCAGTAGCTGCCGAGCGCCGCATGGCTGCTTCGCTTATCCGACTCCATTTCCACGATTGCTTCGTACAGGGTTGTGATG
CTTCCATCTTACTCGATGACAGCCCAACGATCCAAAGCGAAAAATCCGCGGGCCCAAATGTAGATTCTGTAAGAGGGTATGATGTG
ATAGAGACCGCCAAACGTGAGGTTGAGAGTATATGCCCTCGCAACGTTTCTTGCGCTGATATTGTAGCGTTAGCAGCACGTGACGC
CTCTGTCGCCGTCGGCGGTCCGACATGGACTGTAAAGCTTGGGAGAAGGGACTCTACCACCGCTAACCCTAACGAAGCCAACACAG
ATCTTCCTAGTCCGTTCGCTAGTCTCCAAACTCTCATCACTGCCTTCGACGATAAGGGACTTAGTGAAACAGACATGGTCGCCCTA
TCAGGATCACACACAATAGGTCAATCTCGATGCTTCTTGTTCCGCTCTAGAATATACAGCAACGGAACCGACATCGATCCCAACTT
TGCCAGCACCCGGAGGCGCCAATGCCCTCAGACAGGCGGTGACAATAATTTGGCGCCGCTAGATTTGGTGACGCCAAATTCATTCG
ACAATAATTATTTTAGGAATTTGATCCAACGGAAGGGACTTCTCGAATCTGATCAAGTCCTTTTCAATGGAGGATCCACAAACGCT
CTCGTCACTAGTTATAGTAACAACCCACGACTCTTTGCGACAGATTTCGCAAGTGCAATGGTCCGAATGTCGGAGATTCAACCCCT
CCTCGGATCAAATGGGATCATAAGGAGGGTTTGCAATGTTATCAACTAGTTATAAAATGTTTTAATTACTGCATGCATGCCACTAA
GCAATCAAATCAATGTTGGGGTTAATAAAGCGAGTGAAATCTGCTATTCTGTAATTGTGTATATCTTGTTTATCGCATTTTCGTAA
AATGTTCTTTGTCGTTATTTGCTCATGGAATAATATTTATTGAGATTTGTTATATTTTTCGATTAACAATTGTACTTGTGTATTAG
TGTTCATCTTGATTAACCCAATAATAGATCATATGTGCCATGGACTTATTC
>PaPRX2 Phelipanche aegyptiaca Pa.c24858_g1_i1.13544
TGATGAAAAAGCAGAAAATTGTTAGTCTTTCACTAAACTTCAGCGCCAAGCAATTAAGTTTAGTGAAAGACAACAATTTTCTGCTT
TTTCATCAATATGGAGAACAAAAAATCCATCAATTCTGTTGCTGTGACGCTAGCAATATTATCACTCATACTCTTGCTGTCCAGCA
CACCAACTCAAGCACAACTATCTCCCACATTCTACTCTCGCACATGTCGTAATGCGCCAACTATAATTCGCAATTCCATCCGCAGA
GCAATATCACGTGAGAGGCGAATGGCGGCCTCACTCATTCGTCTCCATTTTCATGATTGCTTCGTTCAGGGATGCGATGCATCAAT
CTTACTAGACGAAACACCTTCTATTCAAAGTGAAAAGACTGCATTCCCAAATGTCAATTCCGCAAGAGGGTTTGATGTGATAGAGG
CTGTCAAGCGTGAGGTTGAACGTATATGCCCTCGAGTTGTTTCTTGTGCCGATATTCTAACTTTAGCAGCTCGCGATGCCTCCGTT
GCTGTCGGGGGTCCATCATGGACTGTGAGGCTCGGGAGAAGGGACTCTACCACTGCTAATCGCGGTCAAGCGAACACTGATCTTCC
TAGTCCGTTTGCTGGTCTCCAAGCTCTCATCACTGCCTTTGCTGATAAAGGACTTAGTGAAACAGACATGGTTGCCCTATCAGGAT
CACACACGTTAGGTCAAGCCCAATGTTTCTTATTCCGTGCTAGAATATACAGCAATGGAACTGATATTGACCCCAACTTCGCCCGC
AATCGAAGACGCCAATGCCCTCAGACCAGTGGCAACAGTAATTTGGCGCCACTCGATTTGGTGACCCCAAATTCATTCGACAACAA
TTATTTTAGGAATTTGATCCAAAGGAGGGGACTGCTTCAGTCAGATCAGATCCTTTTTAGTGGAGGATCCACAAACACTCTCGTCA
CTCGTTATAGTAACAACCCACGACTTTTTGCTTCAGATTTTGCAAGTGCAATGGTCCGAATGTCAGAGATCCAACCCCTCCTCGGA
CCGAATGGGATCATAAGGAGAGTTTGTAATGCCACCAACTAGTTTGTTACTTAAAAACTTGTCACAACCACTATTTAGCAATCATA
TTATTTTTGGGATTATTATATAGCAAATAAATCAGCTGTTCAACAATTGTATGTACTTCTGGTACTATTCATGTTATGTCCAATGT
TGTGAAGCCATTATTTTCTGATAATGTAATGTGTCTTTTATTCAAATGTTTTACCATATTGAGTACAAAAAAAAAAAAAAAAAAAA
AC
>ShPRX1 Striga hermonthica Sh.c9817_g1_i1.12343
CAATTTCACCAGCTCCGGAATCAACTTCACCTCAAACCAAATTGTCCAAGTACGTACTTAGTCTTAGCATGGCCACCGAAAAGCCC
ATCAACTTTGTATTCACCCTAACCACACTCACAGTACTACTCATCCTCTCCCTGTCGATAATCCCATCCCGAGCCCAGCTGTCCCC

GACTTTCTATGCCCGCACATGTCCCAATGTGCTGCGTACAATCCGTGGTTCCATCTGGAGGGCCGTGGCTCGTGAGCGCCGCATGG
CGGCCTCACTCATCCGCCTCCACTTTCACGACTGCTTCGTGCAGGGCTGTGATGGTTCCGTGTTGCTGGATGATGGGCCCACGATC
CAGAGCGAGAAGTCGGCTGTCCCTAACCTCAACTCCGCAAGAGGGTTTGATGTGATTGAGGCCGCCAAGAGAGATGTCGAGCGAAC
GTGCCCAGGTGTCGTCTCGTGTGCCGACATTCTGGCTGTGGCCGCGCGTGATGCCTCTGTGGCAGTGGGTGGGCCGTCATGGAATG
TGAAGCTCGGGAGAAGGGACTCTACCACAGCTAACCGTGATGCGGCGAACCGTGAACTACCGAGCCCATTTTCCAGCCTTCAAGGC
CTAATCACTTCTTTTTCCGACAAGGGCCTCAGTGAAAGAGATATGGTCGCCCTCTCTGGATCACACACGATCGGGCAGGCCCAGTG
CTTCCTGTTCCGCAGCAGGATCTACAGCAACGGCACGGACGTCGATCCACTCTTTGCCCGTCTGAGGAGTCGGACGTGCCCTCAGA
CGGGTGGGGACAGCAACCTTTCCCCGCTCGACTTCGTAACTCCCAACAGGCTCGACAACAACTACTTTACGAACCTTCGTCAAAGG
AGGGGTCTCCTTCAGTCGGACCAAGTCCTTTTTAGCGGGGGCTCCACAGACAGTCTCGTCTTCAACTACAGCAGAAATCCCCACCT
GTTCGCCTCCGATTTCGCTAATGCCATGCTCAAGATGTCCGAGATTCAGCCCTTGCTCGGGTCCAATGGGATCATAAGGAGGGTTT
GTAGCGTCCCAAACTAAGTGTGCGGTTAAGTAATGTTTGCAAACACTTCAAAAAGAGAAATAACTAAGAGAGATACTTTTCCTAGT
GTTTGCGAATACTCTGTTTGCGGCTGCGAGGGTTATATATGAAAAGAGGTTATTTTACGTAATTGTATTGATTAATGGAGCATATG
AATATGCTACTCTATGATCTGTAACCTTTAATTGTAAAATGGCTGATCCGTCTTAGTAGTTTGCCATCAAATACTTGTGGTGTATT
CCTTTAATAAATATTTTGAAGGCTTCAGAAAAAAAAAAAAAAAAAAAAAAA
>ShPRX2 Striga hermonthica Sh.c9802_g1_i1.12342
AACAATCAAAACAAGCACCAATTTTCCTTAGTAAATATTACAATAACAATGTCAGCAACGCGTTTTACCGATCAGCAGTGACCAGA
TTAAATTCAAACACACACACACACGTTCATCCCTGCTCCCTATAAATTCAGCACCCAACTTCTCAATCACCTTCATCTCCAAGTAA
ATTGACTTTAGCACTTGTACGTGTCTTTCGATTTCAATTAGTTTCTATATCTCTTTTTCGCAACATGGGCACGACGAAAGCCGTCA
ACTTTGTCCTAACCTTAACAACAATCTCACTACTCATCTCCACCACCCCGACCCAAGCCCAGTTGTCCCCGACTTTCTATAACCGT
ACATGTCCCAATGCACTGAGTACAATCCGTACATCCATCCAGCAGGCGATTTCCCGTGAGCGCCGCATGGCTGCCTCCCTCATCCG
CCTACATTTTCACGACTGCTTTGTACAAGGCTGCGACGCTTCCATCCTGCTGGACGACTCGCCCACAATACGAAGCGAGAAATCGG
CCGGCCCAAACGCCAATTCCGCCAGAGGGTTTGATGTGATTGAGGCTGCCAAGAGAGCAGTCGAGGGTATTTGCCCGGGCGTCGTC
TCGTGCGCCGACGTTTTAGCCGTGGCTGCACGTGATGCCTCAGTTGCTGTTGGAGGGCCGTCATGGAATGTAAGAGTTGGGAGACG
GGATTCTACGACTGCCAACTTCAATGCAGCAAACACAGATTTGCCGGGACCTTCTTCTAGTGCACAAGGCCTCATTACTGCTTTCC
GCAATAAGGGACTCAGTCAGACAGACATGGTTGCCCTCTCAGGGGCACACACGCTGGGGCTAGCCCAGTGCCAATTTTTCCGGGCC
AGAATCTACAGCAACGGCACGGACATCGACCCCAACTTTGCAACCACAAGAAGAAGCACATGCCCACAGTCGGGCGGGAACAGCAT
CCTTGCCCCGCTCGACATCCAAACCCCCAACTCGTTCGACAACATCTACTACAGAAACTTGGTCCAGCGGCGGGGGCTCCTCCAGT
CGGATCAGGTACTTTTCACCAGTGGGTTAACCAATGCCCTAGTCAACACCTACAACAACGACAGTCGGCGCTTCGCCTCCGACTTC
GCGAGCGCCATGCTCAGGATGTCTGAGATTTCGCCGTTGGTGGGGTCCCGTGGGGTCATAAGGAGGGTTTGCAATGCCCTCAGCTA
GACGATGTGGCTGTGGTGCTGTCAGGGTTATATAATGAATTATTAAAACCACTGAAATGCAGTGGCTTTATTATGTGATTTAATTT
GTTTCTTGTCCGCTTAATTTTTCATTTGTTGTCAGTCTTGGTTTTTTTTTTTTTTTTTTTTTCCTTTTTTTGTGAGTGCCAGTAACT
CCGATAACGCACCTAGCATACCGGTCCCGTCAATCGTCCAGCAGCCGGCGATCTTGGTTTAGCAACGACTCTGTCCGACCAAGCCA
AGGTCACCTCGCCATCGCCGTTAACATCACGTGGGTCGGCAGTTTCAAGCCATCGAGACCTGTTTGAAATTTCAGACCCCGTCACC
CTGTCTCGTCCATCCTCGAGCAAAACCGCCGACGAACCAGTCGAGAAATTCTCGTCGGCGACCTTCAACAGCAGTGGCCCTCCTTC
TGTCCGTTAACGTCGGTCAGTGAGCAGCGACGGTTTCCTCCGAGGAGTTTCGCCACCAAAGTCGGAAAATCAGCATAGCCGCTCA
CCAGGAGTCCTGTTCGCAACGAGCAGTTTCTCAGCCGTCGCCGGCGATCCACCACCACCGCGCGACTCCTTCTGGCGAGCAGCAGG
TCCCGTCGGTTCGCTGTCGCGTCTGATCTCTTGTCACTTTCGTAGGCAAGCGAACAGGTACCGT
>TvPRX1 Triphysaria versicolor Tv.c5216_g2_i1.11956
TAACTTTTTATTCTTTCTTGTCAACGCTTAATTTCAAAATGAAGATCAAAAGCTTAGACTCCATAAACATTGCCACTATATTCACA
ATCACAACTCTCTTATTCTTGCACAATTTCATGCCATGTGAAGCACAATTATCTTCAACGTTCTACGACAGAACGTGTCCAAACGC
ATCTACTATCATCCGTAATTCCATCCGTCGAGCAATAGCTCGTGAGAGGCGAATGGCTGCATCCCTCATTCGCCTCCATTTTCATG
ATTGTTTCGTTCAGGGTTGTGATGCATCGATCTTACTCGACGAGACACCTTCCATCCAGAGCGAGAAGACTGCTTTCCCGAATGCC
AATTCTGTAAGAGGCTATGATGTCATCGAGGCGGCAAAGCGCGATGTTGAACGTGTTTGTCCTGGCGTCGTCTCTTGTTCCGATAT
TCTAACATTAGCCGCACGTGATGCCTCCGCTTACGTTGGTGGTCCATCGTATACCGTTCGTCTCGGGAGAAGGGACTCAACCACTG
CGAGTAGAGCCCAAGCAAACGTAGAACTCCCAAGCCCGTTTGCCGGTCTTGACGGCCTCATTTCCGCTTTCGACAACAAAGGACTT
AATGCAAGAGACATGGTTGCCTTATCTGGATCACACACATTAGGGCAAGCTCAATGTTTCTTGTTCCGCTCAAGAATTAGCACCAA
CCGAACCGATATTAACGCCGGATTCGCTAGCACTAGAAGACGACAATGCCCTCAAGTCGGGGGCGATGCAAACTTGGCCCCACTTG
ATTTGGTGACCCCAAACTCGTTCGACAACAATTATTTCAGGAACTTGGTCCAGAGGAGGGGTCTTCTTCAGTCTGACCAAGACCTC
TTCAACGGAGGGTCAACTGACAACATCGTCTCCGAGTATGTTAGGAACCCTCGACTTTTCGCTTCTGATTTTGCGAGTGCTATGAT
CAAGATGGGCGAAATTCCAGCAACACAACAAAATGGGATCGTAAGAAGGGTTTGCAGTGCCATCAACTAAGTGATCGATATATCTT
TATTCAAAACTAATTATTGATGGTTACATTGCTGATTTCATTTACTTTTTTGTCTTCATTGTTTATTAATTGTATTTGTAATAAAA
ATAACTTGTGATTTTTGTACTACTATTCTTTGATTTATGGTATTAAATTATGTAACTCTCATTTTTAAATTAAT
>TvPRX2 Triphysaria versicolor Tv.c5326_g1_i1.12010
ACTTGTAAAATACATCTTCAATTCAAATTCATCTCATTCAATTTTCCTTTTGCTCTATAGTTTTGTGATCTTTCAAACTCCAAATT
ATTTCAATATGAAGACCATAAACTTTCTTGCAACCCTAACAATATTATCACTAGCCCTCTCCATTATTATCACACCAACTCAAGCA
CAACTATCTCCCACGTACTATACCGCCACGTGTCCGACTCTCGTAGCCGTAATTCGTACATCAATCCGTCAAGCGATTGCCCGTGA
GCGGCGTATGGCCGCATCGATTATCCGTCTTCATTTCCACGATTGTTTCGTGCAGGGATGTGATGCCTCGATCCTTCTAGACAACG
CGGCGAATATCCAGAGCGAGAAGTCCGCGGGGCCCAATGTTAACTCGGTGAGGGGGTTCGATGTGATCGAAGCCGCCAAGCGTGCG
GTCGAGGCCGCATGCCCCGGTGTCGTTTCTTGCGCGGATGTTCTAACTATAGCGGCTCGCGAAGCGTCCATTGCTGTTGGTGGGCC
AACATGGAATCTGAATCTTGGAAGACGGGACTCGACCACGGCTAATCCTAATGCAGCCAACTCGGATCTTCCTTCACCCTTTTCTA
CTCTCCAAGGTCTCATCGACGCCTTCTCTAATAAGGGACTTAGTGTCACCGACATGGTTGCCCTATCAGGGGCTCACACATTAGGC
CAAGCCCAATGCTTCTTGTTCCGCGGTAGAATAAACAGCAACGGGACGGACATCGACCCTAACTTTGCTAGCACACGGAGACGCAC
GTGCCCATCGACCGGTGGGGACAGTAATTTGGCCCCGCTCGATTTGGTGACCCCAAATTCGTTCGACAACAACTACTATAGGAACT

TGGTTGCACGTAGGGGTCTTCTTTCATCTGACCAAGTCCTTTTCACGGGGTCCACGGCCGCCACGGTTACACGTTATAGTACTCGA
CCCAGAGATTTTGCAACGGATTTTGCAAGTGCGATGGTTAGAATGTCGGAGATTTCGCCCACTCTCCGACCTAATGGGGTCGTACG
AAGGGTTTGTAATGTCATCGGCTAGTTAATTAATTAAATTATGGGAGTCAAATCTGCTAGTCGATGTGTAATTGTAAGATAACTAT
TTATGGTGCATGTGTGTTTTTGTGTAATTTCAATATTGGTTGTTGTTACTTGTTGATCGTTAAATTGTGAAATAAAATTGATGTGA
TTTGTTGTGTTTTGTCAATCAATGGTTTCTTTAATTATTTTCGTGTCTTGTAGTTGTTGTCATTATTGATGTAATTTTATATTTTT
T

>TvPRX3 Triphysaria versicolor Tv.c105997_g3_i1.21359
TTAGCATCATCCTCTTCATCATCAATCAACTCAATTCATCTTTATTATCCACTTCTAAATCCTTGTTTTTTGCTCTACCCTAATCT
TTCAACATGAATTTTCTTGTAACCCTAACAATATTATCATTGGCCCTTGCCACTATTATTACACCAACTCAAGCCCAACTTTCTGC
GACGTACTACGCCACCACGTGTCCTAACTTCGTCACCATTGTCCGAACCTCCATCCAGGCAGCAATTACCCGTGAGAACCGAATGG
CGGCGTCAATCCTCCGCCTTCATTTCCACGATTGTTTCGTGCAAGGATGTGATGCCTCGATTCTTCTCGACGATACAACCACGATA
CGGAGCGAAAAGAATGCGGGCGCCAATGCTAACTCTGCTAGGGGGTTCGATGTGATCGAAGCCGCTAAGCTTGCCGTGGAGCAAGT
ATGTCCCGGGGTCGTTTCTTGCGCCGATGTGCTCACGCTAGCGGCCCGTGAATCCTCTTTTGCTCTTGGTGGGCCCACATGGAATC
TACTATTCGGGAGAAGAGATTCTACAACAGCTAACCTTGCGGTTGCAAACACCGATCTACCTGGACCTTCTTCTACTCTCCAAGGC
CTCATTACCGCCTTCTCGAGGAAGGGGCTTACTGCCACTGACATGGTTGCCCTATCAGGAGCCCACACACTGGGCCAAGCCCAATG
CGCCTTTTTCCGCTCAAGAATATACGGCACCGCGAACATCGACCCGATCTTTGCCAACAATACGAGACGCGCGTGCCCTCAATCGG
GTGGGAATAGTAATCTATCACCGCTCGACGTTCAGACACCCACTAGATTCGACAACTTCTACTACCAAAACTTGGTCGCACGGAGG
GGTCTACTCCAGTCGGACCAAGTCCTTGCTAGCGGGTCCACAAATGCCACCATCGTACGTTATAGTACCAATCCGGCAGCATTTGC
AGCCGATTTTGCAAGAGCGATGATTAAAATGTCGCAAATTGCACCGACGGTTCGACCGAATGGGATCGTGAGAAGGGTTTGTAGCG
CCATCAACTAGTTACGTGTGTGTGATTAATCACATGCATGCTAATTAAGCAATTTCTTGAGATTAATTAATTATATTGTTGGTGTT
AATAAGTTAAGCTAGTAAAATTGTGCTAGCTAGTTGATTTGTAATTGTTTATTCAGTTACGGTTCATGTTTCGTGTAATGTTTTTT
TAATGTTGTTGTTATTCGTTGTTAATCGTGTTGTCGTGGGATGAACTAAATGTGATTTGTCGTGTTTTTGTCGGTCAACGGTGTTT
TTTTAATTCATGCTTGTAATTTTAATTTTTTATTGTTCTAGTCTATTGTCAATTACATAAAAATGTCTCATATAATATTTTTT

>PaQR1 Phelipanche aegyptiaca Pa.c25149_g1_i2.4599
GGGGAACGTACGCATCACTTTTTTATTTATAATCAATTAATTAATGAATAATTGTGCGCCACCCACTTTCGATGCTGTAAATTCCG
GACGCCACTTTCAAGCTTCTCAGACCTTCTATTTCTTGCTACAATTCTCTTATTTTCTTCTTTAATTTTACAAATCGGTCTCCCAC
AGTCGCTGAAAATTACCCAGTATTTCGAGATTTGAGTATGGCGGGGAAGCTTATGCATGCGGTTCAGTACGACGGTTATGGCGGTG
GAGCTGCTGGTTTGAAGCATGTTGAAGTTCCAATTCCTACTCCTAGTAAGGGTGAGGTCCTGCTAAAGCTGGAAGCCGTAAGCTTA
AATCCTATCGACTGGAAGACACAGAAAGGCTTGCTTCGTCCTCTCCTTCCTCGAAAATTCCCTTTTATACCTGCAACCGATGTAGC
TGGAGAAGTAGTGGAGGTTGGAACTGGAGTCGAAACCTTCAAACCTGGTGACAAAGTCGTTGCAATGCTGAGTCATCTTACGGGAG
GCGGTTTGGCTGAATTCGCAGTGGCCAAGGAAAACTTAACCGTCTCTAGGCCTCCTGTGGTCTCAGCGGCCGAAGGTGCAGGCCTT
CCAGTTGCAGGCCTCACGGCCCACATGGCCCTAACCCAGTCAGCGGGGCTCAAGCTCGACGGAAGCGGACCCCAGAAAAACATCCT
CATCACCGCTGCCTCCGGCGGAGTTGGCCAATACGCCGTCCAGTTAGCAAAGCTGGGAAACGCGCACATAACCGCCACTTGTGGTG
CCAGAAATGTTGACTTAGTCAAAAGTCTCGGAGCCGATGAGGTTCTCGACTATAAAACTCCAGAAGGGGCAGCCCTTAAAAGTCCG
TCTGAGAAGAAATACGATGCGGTGGTTCACTGTGCTTCAGCTTTGCCCTGGTCAGTTTTTGAACCGAATTTGAGCGCCAATGGGAA
AGTAATTGATATAACTCCTGGGCCGAGTGCCATGTGGACTTATGCTCTAAAGAAACTTACATTCTCTAAGAAACAGTTGGTGCCAC
TACTTCTTGTTCCGAAAGGCGAGAATTTGAAGTTTCTTGTTGAGTTAGTGAGAGAAGGGAAGCTTAAGACGGTGATCGACTCTAAG
TTTCCTTTAAGCGAAGCTGCGGATGCTTGGGCCAAGAGCATCGATGGACATGCTACTGGGAAGATCATTGTCGAGCCGTAAATCAA
TGAGAAGAATGACCTTAATATTATTATGCTGTGAGAAACTTTCCTGTTTGATATGTTATTTGATCTGGTCACCTTTTACTCGTGTG
AAAATGTTACCTGGATATCAGAATGCATAGGAATATATAGTCGTTGGTTGATATAATAAATGCTTTTGTGTGTAAGGTTAAAGAAA
GTTGACGAAAGCTCTATGGATTAAGGCACACAGATCAGTTAATGGCTTAGTCGAAATTTTATTCATACTTCTGTTATACTTGTATT
ATATATGCATGAATGGATGTATATGAGTTGAGAAATCTCTGAACAAAAAAAAAAAAAA

>ShQR1 Striga hermonthica Sh.c18735_g1_i1.2952
TACATATATGCTCTTGATTCCATATTCGTTGAAATTATAGTATTTCGTGATTAAGGTATGGCGGGGAAGCTTATGCGTGCTGTTCA
GTACGAGGGTTATGGCGGTGGAGCTGCTGGTTTGAAGCACGTTGAAGTTCCAGTTCCTAGACCTAGTAAGGGTGAGGTCCTGCTAA
AGTTGGAAGCTACTAGCTTAAATCCCATTGATTGGAAAACTCAGAAGGGCGTACTTCGCCCTTTTCTTCCTCGAAAGTTTCCGTTT
ATACCTGCTACTGATGTAGCTGGAGAAATAGTGGAAGTTGGAGCTGGAGTCGAAAGTTTCAAACCTGGCGACAAAGTTGTAGCCAT
GCTGAGTCATACTACAGGGGGGTGGCCTGTCCGAATACGGAGTAGCCAAGGAAAACCAGACCGTACCAAGGCCCCCAGAAGTCCCCG
CTCCCGACGCCGCAGGTCTCCCGATCGCGGGCCTCACGGCCCACATGGCCCTCACGCAGACCGCGGGGCTCAAACTCGACGGTACT
GGGCCTCGGAAAAACATCCTAGTCACCGCCGCCTCGGGTGGCGTGGGCCACTACGCCGTCCAGCTGGCGAAGCTCGGCAACGCGCA
CGTCACCGCCACATGTGGGGCCCGCAACCTCGACCTCGTCCGGAGCCTCGGGGCCGACGAGGTCCTCGACTACAAGACTCCGGAAG
GTTCCGCCCTCCGGAGCCCTTCAGGTAAAAAGTACGACTTTGTGGTCCACTGCGCCTCGGCTTTCCCTTGGTCAGTTTTCGAGCCG
AATCTGAGCGAAAACGGGAAAGTGATCGATATCACGCCGGGGCCTGCCGCCATGTGGACTTTTGCGATGAAGAAAATCACTTTTTC
AAAAAAGCAGCTCGTCCCGTTGCTTCTTTTCCCGAAAGGCGAGGATTTGAAGCGACTGGTCGAGTTGGTGAAAGAAGGGAAGGTTA
AGACGGTGATCGACTCGAAGTTCCCTTTGAGTAAGGCCGAGGATGCGTGGGCTAAGAGCATCGACGGGCATGCCACCGGGAAGATA
ATCGTCGAGCCATAGATTACAGGTAAAATGTTGTTATTACATACATGAGGTAAGAAAACTTGTGGGTTTTTTAAAGAATGTTTTTT
ACATGTTGTGGCTGAACTCTCGTCTGGTTTTAAGAATGTATGGGGACATGTATATGACTTGGTTTGATGAATGAAAAACATGTGAA
TGATATAGTGAATAAAGTTGGCAATTGGGACTTTTTGGATCTCATTATTGTTATAGTGGATCATGGTTGAGTTGTGAACTTTATAC
ATGTACCTATGGTTAGTTTGAG

>TvQR1 Triphysaria versicolor Tv.c107009_g2_i1.16950
AAAAAATCGCTGAAATTTAATTTAATTATGGCCGGAAAGCTTATGCGTGCGGTTCAGTACGACGGTTATAGCGGTGGAGCTGCTGG
TTTGAAGCATGATGAAGTTCCAATACCTAGTCCTGGCAAGGGCGAGGTCCTTATAAAGCTTGAAGCCATAAGCTTAAATCAACTTG

ATTGGAAGCTTCAGAATGGCATGGTTCGTCCTTTTCTTCCTCGGAAATTCCCTTTTATACCTGCTACCGACGTGGCTGGGGAGGTG
GTCCGGATCGGACCGGATGTCAAAAACTTTAAACCCGGTGACAAAGTTGTTGCTATGCTTGGCAGTTTTGGAGGAGGTGGCTTAGC
CGAATACGGCGTAGCAAGTGAAAAGCTAACAGTCCATAGGCCGCCCGAGGTATCAGCTGCCGAGAGCTCAGGCCTTCCCATTGCCG
GCCTTACAGCCCACATGGCCCTAACCCAACACATTGGCCTAAACCTCGACAAAAGTGGTCCCCACAAAAACATCCTCATCACAGCC
GCCTCCGGTGGTGTTGGCCAATACGCCGTTCAGCTCGCAAAGCTAGGAAACACACATGTAACCGCCACATGTGGGTCCCGAAACTT
TGACTTGGTCAAAAGCCTCGGAGCCGACGAGGTTATTGACTATAAAACCCCCGAAGGGGCAGCCCTTAAGAGCCCGTCGGGCAAAA
AGTATGATGCGGTTATTCATTGTGCATCGCCTTTGCCATGGTCCGTTTTTAAACCGAACTTGAGCAAACATGGGAAAGTGATCGAT
ATAACTCCCGGTCCGAGGGTTATGTTGACTTCGGCTATGACAAAACTTACGTGCTCGAAGAAACGATTGGTGACGTTACTTGTTGT
GATCAAGGGCGAGCATTTGAGTTATCTTGTTGAGTTAATGAGAGAAGGGAAACTTAAGACGGTTATCGACTCTAAGTTTCCGTTAA
GTAAGGCTGAGGAGGCTTGGGCTAAGAGCATCGACGGCCATGCTACCGGGAAGATCGTTGTCGAGCCATAAGTTAGTAAGATTTTG
TTTTGTTTTATGATATTGTAATGTGGAATTTGGCTTATGACTTGTTTTGGTGATCTTTATGTTTTGATATGTACTCTTTTGTTAAC
CTACTTGTGGTTAGAGTGGCAATTTGTGTCACCATGGTTGTGTTTGTTCGTGTCCTTAAGTCCTATAATGTAATTTTCATATTTTA
TACTTTATTTAGTC

>PaNQR1 Phelipanche aegyptiaca Pa.c18296_g1_i1.11052
AGAGGCATACACGACACTCATTCAAACATCAAAAAGCAAAAAACCAACTACATAATTCATGATGACAGGCGACGACCGCCGCCTCA
CTTCACTTACCCAGCTTTGACCTTTTACGGCAGAGAAGGAAGAATCCATGGCGGCGGTCTCAGCACCTTCTCCAGTCATCAAAGTT
GCTGCCCTCTGCGGTTCCCTTCGCAAAGGTTCTTACCATCGCGGCCTCCTCCATACGGCGATGGATCTATCAAAGTCAATTAAAGG
TTTGGAGATAGAGTATGTGGACATATCACCATTACCATTTCTCAACACAGATCTTGAAGTACATGGGACTTACCCACCAGTTGTTG
AGGCATTTAGGCAGAAGATCCTTGCTGCGGATAGCATACTCTTCGCTTCACCTGAGTACAACTACTCTTTTACTGGGCCTCTGAAA
AATGCAATTGATTGGGCTTCCAGGCCCCCAAATGTATGGGCTGACAAAGCCGCTGCAATTGTGAGCACGGGAGGAGGTTTTGGCGG
CAGCCGATCTCAATATCATCTCCGCCAGACCGGGGTTTACCTTGATCTTCATTTCATCAATAAGCCCGAGTTTTTTCTAAACGCAT
TTCAACCTACTGCAAAATTTGATGGCGACGGCAACTTGATAGATGAGGCAGCCAAGTCGAAATTGAAAGATGTTCTCTTATCCTTG
TACGCATTCACGCTACGACTTCAAGGTAAGTGTGCATAGTTTATCCCACGATCCTTGTTGTGCATCTTAAAGATAATATTATTCCA
AAAATATTCATCTTATTGTACCCGCCCGCGAGTACTTAACCTTTAATTGCTCAAGTTCAAGGTATGTAATGACATTGTTCTTGTAT
CATGTGTTGTTTTTGCATGAAATCTATATGCTAATGCAGTTCCTGTAGAAATTCTACGGGACATCCGGAACTCCTATATATTAAAA
ATCATATGTAGATTTTCT
>ShNQR1 Striga hermonthica Sh.c10873_g1_i1.9809
AAACAAGTGTCCATGGCGGCGATCTCAGCTCCTACTCCAATCATCAAAGTCGCCGCCATCTGCGGTTCCCTCCGCAAAGGTTCCTA
TAATCGCGGCCTCCTCCGTGCCGCTATGGATATATCGAAGTCGATTGAAGGGTTGGAGATTGAGTATGTGGACATATCACCATTAC
CGTTTCTGAACACGGATCTTGAGGTAAATGGGACATACCCACCTGCTGTAGAGGCATTTAGACAGAAGATTCGTGCTGCTGATAGC
ATACTCTTTGCGTCGCCCGAGTACAACTACTCTATCACTGGACCTCTGAAAAACGCGATCGACTGGGCTTCCAGGGCACCAAACGT
CTGGGACGACAAAGCTGCTGCAATCGTGAGTGCGGGCGGTGGTTTTGGCGGTGGACGGTCACAGTATATTCTCCGCCAGACGGGGG
TTTACATCAACCTTCATTTCATCAATAAGCCGGAGTTCTTCCTGAACGCATTCCAAACTCCCTCTCCATTCGATAGTGATGGCAAC
TTGATCGATGATGCCTCCAAGACGAGGTTGAGAGCCGTTCTCTTGTCCTTGCAGGCATTCACAATCCGGCTCCAGGCTTAGTGTGT
GCAGGTATACCTCCCAGTAGGTGAGGTAATATTGTTCAAGTATATTCATTATCAGTATGTGTGTAATGGTACTACTCTTGTATTGT
GTTTGTGTGTTTTAATGGGATTCATGTACTTTGTCTAAAATTTATAGCGATGTATGAATAAGGTTCATCATAAGATTTGATGAACC
GTTTTTCTTGTTAGACATCTTGAATCAGCTGCAGTGCTGTCTTTCAACTACAGACTATCAATAAACGTCGATCTGTGGTCTATTGC
TTGTGGCTTGTGCAATATAAATATTTACTGGTGTGCTAACTGTTGAGCAGTGGTGCATCTAATAGCTCGGTTGACACTTGACAGTG
TTATAAATGTGGTCTCAGTCTTGATTGATAACCAAATAAGACTCGGGTTTTAGTAAAGGGAAAAAAAAAAAAAA
>TvNQR1 Triphysaria versicolor Tv.c5241_g2_i3.1001
CTAGCAACGTATTGATCACAAATAATGACAGGGGTGATTAAGATTGCTTGTCTCTCTGGTTCTCTACGAAAAGGCTCTTTTCACAC
TGGCCTCATTCGTCATGCTATTCATCTGAACATATTTGGTGATGTTGATGTTGATGATATGGAATTCGTGTACGTTGATATTTCGA
ATTTGCCATTGTTGAACACTGATCTTGAGGAAGAAGGGACTTTTCCTCTTGAAGTTGAAGATTTTCGTGATTATATTCTTGGAGCT
GATAGCTTTCTATTTGCTTCTCCTGAGTATAACTATTCCATCTCAGCTCCACTAAAGAATGCACTTGATTGGGGATCAAGACCACC
AAATGTATGGGCTGGTAAAACTGCTGCCATAGTGAGTGTTGGAGGAGGACACGGCGGTGCAAAGTCACACTATCATCTCCGACAAA
TTGGAGTTTTCATCGATCTTCATTTCATCAATAAACCCGAGTTTTTCTTAAACGCGTTTCAGCCTCCAGCAAAATTTAACGGTGAT
GGCGATTTGATTGATCAAGATGCCAAGAATAATTTGGAGGGAGTTCTTTTGTCCTTGAAGGCATTTACCCTTCAACTTCAAGGCAA
AAATTGAAACAACTGAGGTCCATAAGTCTTTAATTTGAAATAAAATATGTTTTGAATTTCAC

>PaFRO1 Phelipanche aegyptiaca Pa.c170304_g1_i1.19665
TTGTAGCAAATCAACATGTACTATGAAGTCATGTTGTATATATACAGCACATAATTTTCCATAGGAACTACAATATATCTCTTTCA
ACAAAGCAACAGAGAAAAAGAAGATCATGGGAAACTATAGAGTCTTAAAGATTATATCCCTTTTGGTGTTCTTTGGATGGCTATTG
CTTTGGACATTGGTGCCAACAAAAGTTTATAAAAATAAATGGACTCCCAAACTAAAAAAAAGTCTCAGCTCAACATACTTCAGAGA
ACAAGGTGTAAACATCCTTCTATTCTCATTCCCGATAATGTTCATAGCAGCTTTCAGCTGTGTTTATCTCCATCTCCAGACCAAGA
AACCGGTAAACGGCTCAAAGAAGAGCGCCGCACGAGCTGAGAAGCGTCACTGTGCATCGTTGAGGCGTCCAATGTTTGTGAACGCG
CCTCTTGGGATCGTCACCGCCGTGGAGGTTCTATTTGCGATAATGTTCGTTGCTCTCCTAGCCTGGTCTCTAGGAAATTACTTGTA
CATCAGCTTCGGCGCTCTTCACATGCACAAAGTCGGAGAGAAAGTATGGCAAGCAAAGTTTAGAAGCGTGTCGTTGAGGCTAGGTT
ACGTCGGAAACACTTGTTGGGCGTTTCTCTTCTTTCCCGTAACAAGAGGGTCTTCGATTTTGCCTCTCCTTGGATTGACATCTGAA
TCGAGCATTAAGTACCACATCTGGCTCGGCCACGCCTCGAATCTCCTCTTCGTGTTGCACACCGTAGGGTTCTTCATCTACTGGGC
GATGACCGATCAAATGTCTGACGCCCTGGAATGGAGCAGCACGTACGTGTCGAACGTGGCGGGAGAGATTGCGACGGTGATAGCGG
TCGTGATGTGGGCCACGAGCCTCGAAAGGGTGCGGAGGAAGATGTTCGAGCTCTTCTTCTACACTCATTACCTCTACACGGTTTAC
ATTGTCTTCTACGTTTTGCACGTCGGACCCGCCTACACGTGTATGATTCTCCCCGGAATATTCCTCTTCCTCATAGACCGTTACTT

GCGCTTCTTGCAATCCCGAGACCATGCAAGATTGCTCGCCGCTCGCCTTTTGCCCTGCGGTGCCCTCGAACTCAACTTCTCCAAGT
CCCGAGGGCTGCAATATAGTCCGACAAGCATAATGTTCATAAATGTGCCATCTATCTCCAAGCTACAATGGCACGCGTTCACGGTG
ACGTCGAACTGCAATTCAGAGCCGGAGCAGTTGAGCGTTGTGATCAAAAGCGTGGGAAGTTGGTCTCAACGCCTTTACAAACAACT
CTCTTCTTCCCCTGAGCACTTGGCTGTTTCCGTTGAAGGACCCTATGGACCGTCTTCTTTCTCGTTTCTAAGTCATGAGTCTCTGA
TAATGGTAAGCGGTGGGAGCGGAATAACGCCGTTCATCTCAGTCATTCGCGAGATTCTATTCCAAAGCACAGATCCCAAAGCTCAC
ATCCCAAAGATTCGCCTCATTAGTGCGTTTAAAAACTCCTCCGATCTCTCAATGTTGGACTTCTTGGTCCCCATCTCCGGTGCATT
CTCCGACCAGATCCCCCAAATAGACCTCCAAATTGAGGCTTACGTCACCCAGGACAACGAAGGGGACAAAGTAGAAGCCCTTCAAA
CCATATGCTTCAAGCCCAATCCGCGCGACTCCCCAGTAACGGCGACTCTCGGCCCAGACAACTGGCTCTGGCTCGGCGCAATAATA
GCGTCGTCGTTTTTGCTGTTCCTTGTCGTTTTGGGGCTCGTCACGCGGTACTACATTTACCCGATCGACATGAGGGACGCAAAGCG
TCCTTACCACTACTCATATCGGTGTCTCTGGGACATGTTTTTGGCGTGCACGAGTGTTTGCGTGGTCTCTAGCGTGGCGTTTCTTT
TGTGCAGGAGAAGAAACGAGGCCAAAGGAAACATTAAGAACCAAATCACCAACGTCGAAATGACGACTCCAACGGCTACGCCGGCC
TCGTGGTCATACGCGGCGGGGCAGAACCTTGACCTTGAGAGCCTTCCTAATCAGTCATTAGCTCAAGCCATGAAGGTTCATTTTGG
CGCCAGGCCTGATCTCAAGAAGATTTTGTTCGAGTCGGAAGCGTCTGACGTGGGAGTTTTGGTTTGTGGCCGAGGAAGATGCGAC
ACGAGGTCGCCAAGATTTGTGCCTCCGGTTCGGCAGATAATCTGCATTTTGAGTCTATTAGCTTTAATCTCTGATTAATCATTTCC
CTTAATTTCTACACAGATCAATCAATATGTAAATGTCGTTTGTATGTATTTCTATCAACAAGCCTGTTTTTTCATTTTTCAACTTG
TAACAAGTTTGTGATCTATGTGTAATAAATAAGTGTATATATTAAAGATCGGAAGAGCGTCGT
>ShNOX1 Striga hermonthica Sh.c18212_g1_i2.3625
AATTCTTCTTGCAATTTCTGAGCCAAGGACACAATTATTAGGTAAGAGAATTATTCCCCATAACTGATTTAACCAACTTCTCTTCA
TTCTTCCTCTCTCTTTCACCTTCCAAAGATCTCTCTTATACTGGTAAAAAATGAGAGAAAACCTCATTGACATGGGATCATCCAAC
ATCGGCTCGGATCTTCCGAGGGACGAATCCGTAAAATGCACTCTCGAGCGTATAGAAGTCGACACCATGGCAAACGACTCGGACGG
GAAAACGGGCCCACGCATTGGAAGGCTCGAATCGGGCGTGGACCGAGGCCTCAGAAGCCTCCGTTTTCTCGACCGAACCACAACCG
GAAAAGAGGAGGACTCGTGGAGAGCCATAGAGAAGCGTTTTCATCAGCTTTCGGTTGAAGGCAGGCTGTTCAAGGACAAATTCGGA
GTCTGCATTGGGTTGGGGGATAGTAAGGAGTTTGCAGAGGAGCTTTACGACGCGCTGGCGAGGCGGAGGAACGTGAGCACGGAAAA
CGGGATAACCGTGAACGATGTTCGGGAGTTTTGGGAGGATATGACAAATCAGGACCTAGACACAAGGCTTCATATTTTCTTTGACA
TGTGTGACAAAAATGGTGATGGGAGGCTATCAGAGGATGAGGTTAAAGAGGTTCTGGTAATGAGCGCCTCGGCGAACAAGCTCTCG
AATTTCAAGCAGCAAGCCGCCACTTATGCTTCGTTAATCATGGAGGAGCTCGACCCGGACCACCAAGGATATATCGAGATGTGGCA
ACTAGAAGCTCTATTGAGAGGAATGGTCGGTTCCGAGCAAGGAAAAATGAAAGCTACCCGAAAAACCGAGACACTAGCGAAGACTA
TGATTCCCAAGGAATACCGAACGCCCGTCAGCAAATTCGTGTCCAAAAATACCGAGCGATTCTTCGAAAACTGGATGAAGATATGG
GTCTTGTGCCTTTGGTCCTTCATAAACATATCCCTCTTCATCTGGAAATTCAATCAGTACAAAAAACGAGCCGCGTTTCGAGTCAT
GGGCTACTGCCTCTGCTCGGCAAAAGCCTCGGCCGAGACCGTCAAATTCAACATGGCACTCATTCTCCTGCCCGTCTGCAGAAGGA
CGCTGACGTGGCTCCGCGAGTCTTTTCTCGGGACTTTTATACCCTTCGACGAGAACATTAATTTCCACAAAATCATTGCTGCTGGG
ATTGTGTTGGGTACATTGATCCACGTCGTAATGCACTGCACCTGCAACTTCGTGAGGCTTGTTTCGTGTCCCGATAATCAGTTTTA
CTCGATTTTTGGGCCAGCGTTCGATTTTCATCGGCCGAGTTATTTAGATCTCGCAGGGACCACGGTTGGGGTCACGGGGATAGTGA
TGCTCGTTTTGATGGTTTTTTCGTTCACGCTTGCGACTCATTCGTTTCGGAGGAATGTGGTGAAGCTGAGGTGGCCATTTTGCCAC
CTGGCGGGATTTAACTCGTTCTGGTACGCGCACCATTTGTTGGCTCTTGTTTATGTGCTCTTGATCGTTCATGGTTACTTCATATT
CATCACCCGAGAATGGTACAAGAAGACGACGTGGATGTACGTGGCAGTGCCGATGCTGGTCTATACGAGCGAGAGAATACTTACAC
TTTACGATCACAACTATAAAGTTGGGATCATCAAGGCTGTGATCTATACGGGTAATGTGCTAGCATTGTACATGAGTAGGCCTCCG
GGATTCAAATACAAGAGTGGGATGTACCTTTTCGTCAAGTGCCCGGATATATCGAACTTCGAATGGCACCCGTTTTCAATCACGTC
TGCACCGGACGATGACTACCTTAGCGTACACATACGAACACTCGGAGACTGGACAACTGAACTCAAGAACCGTTTTGCAACGGCAT
GCGAACCTCGATCGATCAAACCGAGAAGAGGTAATCTAGTGAGAATGGAAACAAAGGCATATTCCGAAACTCTCCAAGACTTCCCG
CGAATCGTGATAAAGGGTCCATACGGAGCTCCGGCACAAAACTACAAGAAGTTCGATATCCTTCTGCTCATCGGTTTGGGTATTGG
AGCCACCCCTTTTATTAGCATTATAAAGGATATAGTGAATAACGAGTCGAGATATGTACTAACAGATGATGCCTCTGGAGGAGATA
AGAAAGGTCCCGAGCGAGCGTATTTCTACTGGGTAACAAGAGAACAAGGATCGTTCGACTGGTTCAAAGGCGTTATGGACGACATA
GCAGAATACGATCACAATCATATAATAGAAATGCACAACTATTTGACGAGCATGTACGAGGAAGGAGATGCTCGGTCAGCACTAAT
AACAATGGTTCAATCGTTACAACACGCTAAAAATGGAGTCGATGTCTTATCGGAAAGCAGGATACGGACACATTTTGCTCGGCCGA
ACTGGAGGAAAGTATTCACTCACTTAACTAGCGTGCATCCTTCTACCCGAATAGGCGTATTCTACTGTGGGAGTCCTACGCTAACC
AAGCCACTCAAGAAGCTCTGTCAGGAGTTCAGCTTGAATTCGTCTACACGATTTCAGTTTCACAAAGAGAACTTCTAAAGCTTGTT
GCGATGCTTTTTGGTCGCTTATTATCAGCATCGGCTTTTGATTGTTCGATTTTTCGAACCTTCGACTAGCATAGAGAAATTAGGAA
GGTAATGAATGAATCTGGAAAGTGACCGAAAGATTTACGGAACTTCCTATATTTGTTAAAAGCTTCAAAGTTCGATTAACTGCTAG
ATCACAAGTAATTTTCTATAACTCCCTTAAAAGAGTG
>TvFRO1 Triphysaria versicolor Tv.c101806_g1_i2.15882
AAAAAACAAAGTGAAAAAGATGAAATATCGTGCAGCATGGCAGCTTTTTTTCATGTTGGTCTTTGTAGGATATATGCTTATATGGA
TTATGTTGCCTAATAAGACTTACAAGGATTCATGGACTCCTAAACTTAACAAACATCTCAACTCCACATACTTTCGCGAACAAGGG
ACGAATCTTGTTTTATTTAGTTTCCCGGTTATGCTCATTGCCTCTTTAGGATGCGTTTATCTACATTTCCAGCAAAAGTCGAATAA
TTCTAAGAGTAATAAGAGCTACTTACAATCATTGAAAACCCCGGCAATGGTAATGGCCCCACTCGGAATAGTCAACGCCATCGAGC
TTACATTCGCAGCCATGTTTATCGCTCTTTTAATCTGGTCATTAGCAAATTACTTGTATGTCAGCTTTGGCCACCTCCACATGCAT
ACGCCCGGAGAAAAAGTGTGGCACGCGAAGTTTCGCAGTGTGTCCTTAAGGCTTGGATACATCGGGAACACATGTTGGGCTTTTCT
GTTTTTCCCCGTGACACGTGGATCGTCGATCTTGCCCCTGATGGGCCTCACATCTGAGTCCAGCATCAAATATCACATTTGGCTTG
GCCATCTTTCGATGGGCCTTTTCGCCCTCCATAGTGTTGGCTTCGTCATCTATTGGTGGTCGATGACTCATCAAATGTACCTGATG
TTGGAGTGGAGCAGTACGTATCTGTCGAACGTGGCTGGGGTGATCGCGTTCGGTCTATCGTTGGCTATATGGGGAACGAGCCTAAA
TCGGGTCAGGAGAAAAATGTTTGAGCTTTTCTATTACACACATCATATCTACATTCCCTTTGTTTTCTTCTACATGCTTCATGTTG
GAGTGGGCTATCTATGCATGATCCTTCCCGGGATTTTCCTCTTTCTCATCGACCGGTACTTGAGGTTCTTACAATCGAGACGGAGG
GCCCGGCTAGTTTCGGCTCGAATTTTACCTAACCGCACCATGGAACTTACATTCGCCAAGAATCCAGGGTTGTGTTATAGTACGAC

TAGTACCTTGTTTGTGCATGTGCCGGTCGTGTGTAAATTACAGTGGCATCCGTTTACAGTAACTTCGAGCTGTGATTTGGAGACGG
ATAAGTTAAGTGTTGTGATTAAAAGTCAAGGGAGTTGGACACAAAAGCTCTATAACCAACTTTCCTCTTCCCTCGTCTGTCTTCAA
GTGTCGACTGAAGGACCTTATGGACCGACATCGTCTAATTTTCTAAGTCGTGAGTCGTTAGTGATGATCAGTGGTGGAAGTGGGAT
CACGCCGTTCATTTCCATAATACGCGAAACCATTTCCGAAAGCACAAAATCAAAAACAAAGGTTCCGAAAATCCTTCTCATCGCAG
CTTTCAAGAACACGGCCGATCTAACAATGCTCGACCTCTTGCTTCCTATCTCAAACACAACAACTCCTTTAGACATTTCCAAGCTA
CACCTCCGAATAGAAGCTTACGTCACTCAAGAGCAAGAAAAACCTCTCGACGACACAAAAACTCAAATCGAGACAAAAATGTTCAA
GCCAAATCCGTCTGATTCGCCGATATCTTCAACTCTTGGTAAAAACAGCTGGCTATGGCTCGGAGGGATAATATCATCGTCTTTTG
TGATGTTTCTACTTCTTTTGGGATTTGTGACTCGTTACCACATATATCCTGTCGAAAGAAGAGGAGAGAATTATCATTACAGTTAT
AAGATTTTATGGGACATGTTTCTTGTGGTTGCTAGTGTGTTTGTGGCTACCAGTGCTGTCTTTTTGTGGCAAAAGAGAGAAATTTC
AAAGGAAGCTGGGAAGCAAATTCAGAATGTGGATATGCTGACACCAGCGATGATGTCACCATCGTCGTGGTTATGTGGTGTGGGTT
CAGATAGAGAACTCGAGAGCCTTCCGAGTCAGTCTCTTGTTCAGTCGACAAACGTGCATTTTGGCTCACGGCCTGATTTAAAGAGG
ATACTTTTGGAGTGCAAAGAATCGGACGTTGGAGTTCTAGTTAGCGGACCTAAAAGCATGCGACACGATGTTGCTAAAATATGTTC
GTCTGGTGACATGAAAAACCTGCATTTCGAGTCTATCAGCTTCAACTGGTGATGCATGTGACATTGTATACCTTTTGTTCGTGTTA
CGTATTTTCTCTTTTCGGATCCTTGCCACGTTTCAATCGATCTGCAAAGGAATGTTGATTTGTGCCTTGGAATTATTTAAATTGAG
TTTGTTACAGCTCTGCGTGCATAAGAATTTGTTTGATTTTATTTTCTGAGTTATGTTGGAATATATTTGAAGCTAATTAAATCTGT
TTGAATGTATCATATAACCATTTG

>PaCSD1 Phelipanche aegyptiaca Pa.c163109_g2_i2.16053
CATCGGCATCCACCAAGTCGATTTTGACTATTTACTACTAATCACCAAGCAGCTTCCAATGATCCGCAGCTACGGGGAGCCCGCGA
AGCTTGTCTAGCCTCCAAATACTGTTTAAGCAGAGATACCGATGACACCGCAGGCAGGGCGGGGGCCAGCGTTGCCGGTCTTCTTA
GACTCCTCGGTGCCACCCCTGCCGAGGTCATCAGTGCCGGCGTGGACGACGACGGTACGGCCGATGATGCTCTCAGCACCAATGAG
CTTGATCAGCTTGTCGGTGGTGGAGCCAACGGCGTTGCCCTGGCCATCGGTCTTGATGTTGCCGAGGTCACCAACGTGGCGGTCCT
CGTCGGTGGGAGCACCGTGGCCCTTTCCGTGAGGGTTGAAGTGAGGGCCAGCGGAGGTGCAGCCGTTGGTGTTGTCACCGAAGGCG
TGAATGTGCATGCCACGCTCAGCGTTGGCGTCGTTGCCGCTGATGTTCCATGAGATGCTGGTAGGAGCAGACTCGGATTCCTGCTC
GAAGGTGACGGTGCCCTTGACGTTGGAGTCACCACGGACGACTGCGACGGCCTTGACCATTTTGACGATTTTGATGCTTTCTTCGG
ACGTTTGATGTGAGAATTGAGAGCTTTTGCTGATGGAGAAGAGGAGAGAAGGTGGACGATGATCAAGCTCTCGCGTTTT
>ShCSD1 Striga hermonthica Sh.c12050_g1_i1.14196
ATCCACCAAGTCGATTTTGACTATTTACTACTAATCACCAAGCAGCTTCCAATGATCCGCAGCTACGGGGAGCCCGCGAAGCTTGT
CTAGCCTCCAAATACTGTTTAAGCAGAGATACCGATGACACCGCAGGCAGGGCGGGGGCCAGCGTTGCCGGTCTTCTTAGACTCCT
CGGTGCCACCCCTGCCGAGGTCATCAGTGCCGGCGTGGACGACGACGGTACGGCCGATGATGCTCTCAGCACCAATGAGCTTGATC
AGCTTGTCGGTGGTGGAGCCAACGGCGTTGCCCTGGCCATCGGTCTTGATGTTGCCGAGGTCACCAACGTGGCGGTCCTCGTCGGT
GGGAGCACCGTGGCCCTTTCCGTGAGGGTTGAAGTGAGGGCCAGCGGAGGTGCAGCCGTTGGTGTTGTCACCGAAGGCGTGAATGT
GCATGCCACGCTCAGCGTTGGCGTCGTTGCCGCTGATGTTCCATGAGATGCTGGTAGGAGCAGACTCGGATTCCTGCTCGAAGGTG
ACGGTGCCCTTGACGTTGGAGTCACCACGGACGACTGCGACGGCCTTGACCATTTTGACGATTTTGATGCTTTCTTCGGACGTTGA
TGTGCAG

>PaCDS2 Phelipanche aegyptiaca Pa.c24060_g1_i1.8178
GTTGAAGTTGAAAATATGTATATTGAGATCCGACAGGTCGAGCTTGTCTCGAGCTCGAGTTTGACAGAGTATGAGTCAAGCTCTGA
CCCGAGTTGCTCGACTCGTTTGCAGCCGTATATGCAATCCATGGTTCGGTCCTCTAAGATAACCTAAGCAGACCCTTTCAGCCAAT
ATTGCTATGTAATACTTAACCTACAACCAAACTTGCAGCCTGACGTGACGTCATTAAACCATAGCTCTGTAATTATTCAACTCTTA
GTGAAGTAAAACTAGTTCCGATGGTAATACCGTAATAAGCATAACACAAGCTTACAATGTTTTGAGAAGGTTATATCAACTCTGAT
AGAGAACTCATAGATAGTTCAATTATTGGTCGATAATGCCGAATAACAGTTATATAATACAACGTAAAATCACACCGATGCCATAG
GCCTCTATATGAACCATAAAGAGTTATGGAGTTCGGACGCACCAACGTGGTTTCGGAACAAGAAGCTACTAAATTACAGTGGAGTC
AATCCAACAACACCACATGCCAAACGCCCGCCAGCATTGCCTGTAGTAAGACTAAGCTCATGACCGCCCTTTCCGAGATCGTCTTC
ATGTTCATGAACAACAAAAGCTCTTCCAACAACTGAGTGAGGACCACTCAACGGTATCTGTGTGTCTACAATTGTCGCTTCAGCCA
CACCCTCAGCATTAGCAATTATGTTTCCCAGGTCACCCGCATGACGAACATCGTCTTCAGGAGCTCCATGTGTCAAGCCATTCGGG
TTAAAATGTGCTCCTGTTGATATACATCCATTTGTGGTGTCACCGTACTCATGAAGGTGAAACCCGTGTTTGCCTGGTGCGAGTCC
AGTCACACGAACACTCACAGTAGTGGGGCCATCGTCTTCTTGAGTTAGAGTGACGACGCCTTCGACGCTCGAAGTGCCTTTGAGAA
CGGCGACAGCTTTGTTGCTGGCGGCGACG
>ShCSD2 Striga hermonthica Sh.c17257_g1_i1.7008
TTTGCAAATGAAAATCTTTCAATTTTGAGTTTTTTAACTAGAGAAAGAAGAGTTGTTTCAACTGTACATGCTTTTTGCCATTTATT
GAAACAAAACCTAGTTCATGCAAATCGCTTAACCATAGTTAGTACCCTTTTTATTAAACTCACTGAAGCAAGTGCCATTACACAAT
TTTCTGAACCACAAGGTTGTAGATTCAAACTGAAGGGTTACATCACTCAATTACTAATAGACAAAGCCAAAATAACAGTAAATACA
ACACAATGTAAAAACATAGTTCCACCATATATGGGCCTCTCTCTCTATATGAACCGCACAGAATCAGGCCCCAAGGGTGGGTTTTG
AACCATCTTACTGTGGGGTCAAACCAACAACACCACAAGCCAAACGTCCGCCAGCATTGCCTGTAGTTAGACTAAGCTCATGACCG
CCTTTTCCAAGGTCATCCTCAAGTTCATGAACAACAAAAGCTCGTCCAACAACTGAGTTGGGCCCACTCAACGGTATCTGTGTGTC
GACAATTGTTGTCTCAGCTACACCTTCGCCATTAGCAACAATGTTTCCCAGGTCACCCGCATGACGGACCTCATCTTCAGGAGCTC
CATGAGTCAATCCATTTGGATTAAAATGTGCTCCTGTTGATATGCATCCATTTGTAGTGTCACCATATTCATGAAGGTGAAAGCCG
TGTTTGCCTGGAGTAAGTCCGGTTATACGAACATTCACAGTAGTTGGTCCATTGTCCTCCTGGGTTAGAGTAACGACGCCCTCGAC
GGTGGAGGTGCCTTTGAGAACAGCAACGGCCTTCTTGGTGGCGGCGACGACGGTGAGCGGCCTGGGAGCGGTGGCGGCTGAGAGAG
ACAAGAAAGGGCGGACTTTGGCCTTTACAGCGACGCCGTTGAAGGAGGAATGGAACGGAAGGGACTGCGACGAAACAGAGGACGTC
GCGGGGAAGAGAGCAGGAAGCTGAGGTGTTTGAACGGCGGCGGAGAGGACTGAGTGGGCGGCCATGGCTGCAAGCGCCGCTTGCAT

CTAAAAGATTGGATTTTGGGGTGGAGAAGTGATGAGATTTGAGTGAGTGGGTGAAGGGTTTACCGAGTCATGTGAGGGCATAACGA
AGGATATGGTATATATTTGTGC
>TvCSD2 Triphysaria versicolor Tv.c6286_g3_i1.7001
TTTTTTTTTTTTTTTACGGTTTGTCACGTCACTGGGCATGAAGCTAGTTTGGTTAATCGGTAAAGAATTCAATATCACGAAAAGTA
CACTTCCCTGGCACATTTCTCATAACATTTTTTAACAAATTTATTCAACTCCAATTGAAGCAAAACTAGTTCCCAATGGTAATGGT
ACAATACAGAACTAACCAGAGAGAGAAATCATTCATACATTGGTAGACAATTCGAAATACCAGTTATCTAATGCAATGCAAGTCAC
TGCGCCACAATAGGCCTCTATACGAGCCATCGAGAGTTACAGCGCACACACGAGGCTGGTTTCAAGCATCTTAAAGTGGCGTCAAA
CCAACAACACCACAGGCTAAACGCCCGCCAGCATTTCCAGTACTAAGACTAAGCTCATGACCACCCTTTCCAAGATCATCCTCAAG
TTCATGAACAACAAGAGCTCTTCCAACAACAGAGTTAGGACCACTTAAGGGTATATTTGTGTCCACGAGTGTTGCTTCAGCCACAC
CCTCAGCGTTAGCAACTATGTTTCCCAGATCACCCGCATGACGGACTTCGTCCTCAGGAGCTCCATGTGTTAAGCCATTTGGATTA
AAATGTGGTCCTGTTGATATACATCCATTTGTGGTGTCACCATACTCGTGCAGGTGAAACCCGTGTTTGCCTGGAGTAAGTCCAGT
TATACGAACAGTCACAGTAGTTGGGCCATCGTTCTCCTGGGTGAGAGTGACAACGCCCTCTACGCTCGAACTGCCCTTGAGAACGG
AGACGGCTTTCTTGGTGGCTGCGGAGACAGTGAGAGGCTTTGGGGCGGTGGTGGCGATGGAGAGAGACAAGAAGGGGCGAACATTG
GCCTTAAGAGCGATGCCGTGGAAGGATGAATGAAGTGAGAGAGACGGCGGAGAAATGGAGGATGATACGTTCGGGAAGAGAACAGC
AAAGTGAGCATTTGAGACAGTGGCGGCGGAGAAAACTGTGTGGGCGGCCATGGCTGCTAGCGTCGCTTGCATCAGAATGGATTGGA
TTGGATTAGTTGATAATGTGAGCGAGTGAGTTGGTATAATTCAAGCGAGTTTTTTTTTTACCTTTGTTATAAAGGCACAAATATAG
GTACGTGTTAG

>PaGPX4 Phelipanche aegyptiaca Pa.c20866_g2_i1.5606
TTTTTTTTTTTTTTTTTTTTAAAACGAAAAGGTAATTTGTTTCATAAGTTATATATTTATTTTGTTTGTTACAATATCCCTAAACC
AAAATTACACACAGACACATTACAATACAATTACAATTACACAAGCATTCACCAACTGAAAATTGAGTAAAAAGAAGACACAAACA
CACACCTAGTTTTGCAGAATTCTATAGGTCTTCAAGTTTCACCCAAAGCTTCCTTTATGTCACCCTCAATTGCCGATGGTGAGGTA
GACGGTCCATAACGTTTGATCACATGCCCATCTTTGTCGACTAAAAATTTGGTGAAGTTCCATTTTATGCCGGAACCAAAATAACC
ACCTTTGCTTGCTTTAAGGAACTTATAAACCGGTGCTGCATCTGCTCCATTCACTTTTACCTTTTGAAATATGGGATACTCAGCTT
TGAATCTTGTACAAGCAAAATGTTCTGCTTCTTGGCTCGTTCCTGGCTCTTGATACAAGAATTGATTGCATGGGAAGGCCAATATC
TCAAAACCTTTATCCTTGTACTTAGAGTAAAGCTCAGTCATCTGCGTGTAATTTGAGTTCGTCAACCCACATTTAGACGCAACATT
AACAACAAGCAAGACTTTGCCTTTATAGATATTCAGATTCGCATCATTACCTTTAAAGTCCTTGACGTTGAAATCGTGGATCGATT
TTTCCGTGACCGATGAAGAAGCACCCATTGTGTTGTTTCTCTGTTTAAGCTCTCCTGTTTTTCCTTCCTACGCTTGACAAATACTG
CACTGATGCATCAATAATTGCA
>ShGPX4 Striga hermonthica Sh.c2340_g1_i1.4308
AAAAAACCCATTCCAAAGGGTTACCATCATTATAAACAACCAGATTTTCATGCCATATCACAAAACAATGCCATAAGAACCATTAA
TACAAGCACTCTATTAATGGAGGGAAATGGGTGAAAAAGAATACACACATCTAAGTATTTTTTAACTTTTTTTGCTGCTGTCTACA
GTTTTGCAATACCATAAAATTTTAAGGTCATAATATAACAACAAAACCGAATGCTCGCCTTTCAATCTTCACCGAGAGCTTTTTTTT
ATATCACCCTCGATTGCCAAGGGTGGGGTAGAAGTCCCATAACGTTTGATCACACGACCATCTTTGTCAACTAGAAATTTGGTGAA
GTTCCATTTTATGCGAGACCCGCAAAAACCACCTTTACTTGCCTTAAGGAACTTATACACAGGTGCTGTATTTGGCCCATTCACTC
TTATCTTTTGAAAGATGGGATACTCAGCTTTGAATCTTGTGCAAGCAAATTGTTCTGCTTCTTGGCTTGAGCCTGGCTCTTGATAC
AAGAACTGATTGCACGGGAAGGCCAATATCTCAAAACCTTTATCCTTGTACTTGGAATAAAGCTCAGACATCTGTGTATAATTTGA
GTTCGTGAACCCACATTTGGACGCGACATTAACAACAAGCAAGACCTTGCCTTTATAAACACTCAGATTCACATCATTGCCTTTAC
TGTCCTTGACGTTGAAATCGTGGATCGAATTTTGTGGGGGGACTGAAACTGAAGAGGAAGAAGCACCCATTATTATGCTATCTGTT
TTATCTGCTTCTCTACGCTTGACCAGATAATCACAGGCCACATAAAGGCATTCATTCATTCACTTGTTATTGCAAAATGCGGAGGT
TTGAAAATTTTTTGTTGATGCTAAATGTGAAAACAGAGGAGAAAAGCCCCTTTATCACATGACAGGCTGGCACG
>TvGPX4 Triphysaria versicolor Tv.c4008_g1_i1.95
CCATAACGTTTAATGACTTGCCCCTTAAAACCCCAACATTTTTCAACCCTTTCACAACTACCCCACAACAAGAAAATGGGCGCTTC
TCCATCGGTCCCAGAAAAATCCATACACGAGTTTACTGTAAAGGACAGTAAAGGTAAAGATGTGGATTTGAACGCCTACAAAGGGA
AGGTCTTGCTTGTTGTTAACGTTGCTTCCAAATGTGGGTTTACAAATTCAAATTACACCCAGTTGACTGAGCTTCATTCTCAATAC
AAGGATAAGGGTTTTGAGATATTGGCATTCCCGTGTAACCAGTTCATGAATCAAGAACCGGGAACGAGCGAAGAAGCCGAGCAATT
TGCCTGCACAAGGTTCAAGGCTGAGTATCCCATCTTCCAAAAGGTGAGAGTGAATGGAGCAGAAGCAGCACCTGTATATAAGTTCC
TAAAATCGAGTAAAGGAGGTGGTTTCTTTGGCTCAAGCATAAAATGGAACTTTACTAAGTTTCTAGTCGACAAAGATGGGCAAGTC
ATTAAACGTTATGGAACAGCCACTTCGCCATCTTCAATCACGGATGATATCAAGAAAGCTCTGGGAGAAATTTGAAGGAGTTGTGT
TAATTTGATTTGGATGTTAGATACAAAGCTTTCTTCATTGCTCGTTTGTGCATTTGTTTTTCTTATTTTATCAAATTGTTTGAACC
AGAAG
>TvGPX5 Triphysaria versicolor Tv.c106689_g1_i1.19515
GAATAACACTAAAAAATCATAGTTTTGCATGTAAAAAAAGAAGAATTAAGAGAAACCCTTTTTCTTATGCCCACAACATTTTATTT
CGAAATAATATATCCTCCTATATCCAGTCCTTGAAGACAAATCATCCAATAAAAAACTATAGAATATACAATACAGCCATATGTTT
GTATGTCATATCCAAAACATCAAACTTACCATTTTACAATACTTGCTTTAATTATATGGATCAATTAATTAGCTTCGTGTTTATAC
TTCACCCAGGGCTCTTTTTATATCACCCTCAATTGCCAGCGGCGGGGTAGAAGGTGCATAACGTTTAATTACAAGCCCATTTTCAT
CAATTAAAAACTTGGTGAAGTTCCATTTTATGCGGGACCCAAAACAACCACCTTTACTTGCTTGAAGAAACTTATAAACTGGTGCT
GCATGCGAGCCGTTCACTTTTACCTTTTGAAAGATGGGATACTCAGCTTTGAATCTTGTACAAGCAAATTCCTCTGCTTCTTGGCT
CGACCCTGGCTCTTGATACAAGAATTGATTGCACGGGAAGGCCAATATCTCGAAACCTTTATCCCTGTACTTGGAATAAAGCTCAC
TCATCTGCGTATAATTTACGTTTGTGAACCCACATTTGGACGCAACATTAACAACAAGCAACACCTTGCCTTTATAAATCTCCAGG
TTCACATCATTACCTTTACTATCCCTGACGTTGAAATCGTGGATCGATTTCTGCAGGATTGAAGAAGAAGCGCCCATTTTCTTCTC
TGTTTGGCCTTCCTTGCGCCTGGTAAAACGGCGTCGTACGCACGTGAAAGCTTGATTAAGAATAATTATTGCAGGCAAGGAAATAA

ACGGCGTGCTACGATGTGAAAATAGAGGAAGGGTGGTGATGGTGAGTGGTATGGATGAGTGAATAATCAATCATCTGAGAATTGTT
GGATATTCTAAAAAAAATCAAAT

>PaGPX6.1 Phelipanche aegyptiaca Pa.c182494_g1_i3.24732
ATTTATTTCATATATTAAATTAAAATGACAGATTGCTTGCAGGATTTGAATCCATCTAATACGAAATGCTCATCAAGTAACTAGAT
GATGATTGATTGCTACATTGATATACCTATATAAATTGATTAGGTTGTCTATTAATTCACAGTTCGGCATTCCCCACATGATGGAA
TCACTGAGATAATATCAAAATATTTGCATACACTACTAAAGAAAAGAGAGAACTGCATTGTAAAACATCAGTAATATCCCGGAAAA
GATCTTACACAAACTCCGTACACTTTATTCACTCAAATATTCTCCACGGAGATCGTTATTAGTAGTAGTAGTAGTATGTATCGAGT
TACATACAGTCATGTGTACTCCTTACTCACGCTGTTCTTTGATAACCTTTCTTCGTTATTTTCTTTATATGTTTGCACCACTCTCC
AGGAGTTTCTTTATATCCTTCTCTATGCTGAGAGGAGAGGTAGTGGGCGCATATCGATCAACAACATGCCCCTTTTGATCAACAAG
GAATTTGGAGAAGTTCCATTTAATGTTATCCCCCAAAAACCCTCCTTTAACTGATTTCAGGTATCTGTATAGTGGAGCAGCATTTG
GACCATTCACCTCAATCTTGTCAAATATGGGATACTCTGCCTTGAAACGAGTGCAAGCAAAGTTTTGGATTTGTTCATTGGTACCA
GGCTCTTGTGAACCAAACTGATTGCAGGGAAATGCAAGAATCTCAAAACCTTGATCCTTGTACTTCTCGTATAACTTGGTGAGCTC
GGTGTAATTTGAGTTGGTCAAGCCACACTGTGATGCAACATTGACAATTAGAAGGACTTTTCCCTTATATGTACTCAGATCAACAT
CATTCCTCTGGGCATCCTTGATAGTGAAATCATGGACAGATTGAGTCTGGGTTGACTGGCTAGCCATCGTATGATCCGCTCTGAAG
CCTAAAAACAAAGACTTTCTCGAACTAATACCAGATGAAGAGTACAAAGACCTAGCTTTGATTGGCTGAAAAAGCAAACAATTCGA
CGCAACAGCTGGTGAATTAAATTGGATTCTCGCCAAGATCGCATAAATGGAGCCC
>PaGPX6.2 Phelipanche aegyptiaca Pa.c21890_g1_i5.5938
CATAAAATTTCATAAAATCTTCCATTTTTCATTTTTTCTTCTAAGTGAGGGGGCATTGTGGTCATTTTGGATAAAAATCAAGTTTT
AACGCCATGAAGCATAAAATCCAAAATTTCCCAAATATAGACTACTTCAAAACCACTGAACCAGACACATTTTCATTTCAACACCA
TGACATCAAATTCTCAGTCATTTTCACATAGTTTCACCTTACACAAATCCAGAATCCTCGTTCACTTGCATATTCTCCAAAGTGAT
AATATTATTAGTAGTAGTAGTATTTATTGAGTCGTACCATACAATCACGTATACACCCTTTCCATAATTCATTGATCACCTTTCTC
CATCATTTTCTTTATATGTTTGCACCATTCTCCAGGAGTTTCTTTATATCCTTCTCTATGCTGAGAGGAGAGGTAGTGGGCGCATA
CCGATCAACAACATGACCCTCTCGATCAACAAGGAATTTGGAAAAGTTCCATTTAATTTTATCCCCCAAAAACCCTCCTTTGACTG
ATTTCAGGTATTTGTAGAGTGGAGCAGCATTTGGACCATTCACCTCAATCTTGTCAAATATGGGATACTCTGCCTTGAAACGAGTG
CAAGCAAAGTCTTGGATTTGTTCATTGGTACCAGGCTCTTGTGAACCAAACTGATTGCAGGGAAATGCAAGAATCTCAAACCCTTG
ATCCTTGTACTTCTCGTATAATTTGGTCAGCTCAGTGTAATTTGAGTTGGTCAGGCCACACTGTGATGCAACATTGACAATTAGAA
GGACTTTTCCCTTATATGTACTCAGATCAACATCATTCCTCTGGGCATCCTTAATAGTGAAGTCATGGACAGATTGAGTCTGGGTT
GACTGGCTAGCCATCGTATGATCCGCTCTGAAGCTCAAAAACAAAGACTTTCTCGAACTAATACCAGATGAAGAGTACAAAAACCT
AGCTTTGATTGGCTGAAAAAGCAAACAATTCGATGCAACAGCTGGTGAATTAAAATGGGCGCCTAAGCCTATGGGTCTGATCAAAT
TCCTGTTTCTCACAAGGCAAGTTGAGCAAAAGGACATGAGGAGATATATATAAAGGAATATAAAAAAGAATTACGGGTTCCAAATG
GATTTGGATGTGAAAGCGCATACGATACATGCAAGAGAGATTGGCAATGGTATGATTTGCGTGGCTGGACATGCTAATTTACGCGT
GACATAGTGGAATGTACACCACGTGGGATGCCTTCCAACTATATTTCGATACTCCACCA
>ShGPX6.1 Striga hermonthica Sh.c13821_g1_i1.4424
TTATCCAACCTTTCAGTTTTATCCGAACTTTTGCGCAAGCGAACCAATTTCGATCCACTGCATAACTATCCAACCTTTCAGTTTTA
TCAGCCGATCGAAGATAATAACAATATGGCCAGCCAGAGCCAGTCCAGCCCGGCCCAATCAATCCATGAATTCACTGTAAAGGATG
CCAAGGGCAGTGATGTCAATCTGGGTACTTATAAGGGCAAAGTCCTGCTGATTGTCAATGTTGCTTCACAATGTGGTCTGACCAAT
TCAAATTACACTGAGCTGACACAATTATACGAGAAGTACAAAGATCAAGGTCTAGAGATACTGGCATTTCCCTGCAATCAGTTTGG
CTCACAAGAGCCTGGCACCAATGAAGAAATTCAGGAATTCGTGTGCACTCGGTTCAAGGCTGAATATCCTGTATTTGACAAGGTCG
AGGTGAATGGTTCGAATGCTGATCCAATATACAAGTACATGAAGTCGATCAAAAGCAGCATTTTTGGGGACAGTATCAAATGGAAC
TTCTCCAAATTCCTTGTGGACAAAGAGGGCCGAGTTGTCGACCGTTATGCTCCCACCACATCTCCTCTTAGCATTGAGAAGGATAT
AAAGAAACTCCTTGAGAAGGCGTGAGCGTTGGAGGTAGCCAAGATCAATATGTTTCTGTTGGTGGTTAATATGAATAGTCGTGTTA
ATAAGAATTTGACTTGATGTACGCCTTACGACTTTGTTTGAAATAAATTGCCATCATACTTAAACAACTTGGAATAATTAATTTTG
CTGCTGTTATTTGGATAGCTGATCAGATCATCTTCTACCAGTGACATATATAGTACCATCTGTGATTATATAAGCAGAACTACTTT
AATAGCCGTTCTTGTGGATCCCAGTTTTTACTTCGTTCGTTG
>ShGPX6.2 Striga hermonthica Sh.c13847_g1_i2.4434
CAAAAGTCAAAACTTCCAGAAACTAGAAGTATAAAGTAAATAAAATCGTCTATACGTTGAAACTGATTGGCCACATTGTCAACACG
TGGCGTGCATCCAGCCTCTTACGCGTACATTAGCAACGTCACATATCTTGCGCCATCGTTCAAATCTGCATTCACATTTGGACCCA
TAATAAAGAATAGTAGTACTCTTTATTTCTTTTCTTTATATATATCTGTTCATTCATGTCCCTTTTCAAATCTCGCATTCTGATAA
ATATAAGAACCTCATACATCACAGGCTTAATTTCTTCTTCCCCGATCAGAACACATCTCGATTCACCAGTTACCCCGCCGGAATCT
GCGCGTTTTGAGCCCATTAAAGCTAGGTTTCTGTACGGCCCTTCATCCTGTGTTTCTTCGAGGAAGTCTTTGATTTCGAGCTTCAG
AGCAGATCGTACGATGGCCAGCCAATCAGATTCGCCTCAATCTGTGCATGGTTTCACTGTTAAGGATGCCAAGGGGAATGATGTCG
ATTTGAGTACATATAAGGGAAAAGTCCTTCTAATTGTCAATGTCGCATCACAATGTGGCCTGACCAATTCAAATTACACCGAGTTG
ACTAAGTTGTATGAGAAGTACAAAGATCAAGGTTTGGAGATATTGGCATTTCCATGCAACCAGTTTGGCTCACAAGAGCCCGGTAC
AAACGAACAAATTCAAGAGTTTGCTTGTACTCGTTTCAAGGCTGAGTACCCCATATTCGATAAGATTGAGGTAAATGGTTCAAATG
CTGCTCCACTATACAAATATATGAAATCAGTCAAAGGTGGGCTCTTCGGGGACAGCATCAAATGGAACTTCTCTAAATTTCTTGTT
GACCAAGAGGGTCATGTTGTTGACCGATATGCTCCGACTACTTCCCCTCTCAGCATAGAGAAGGACGTCAAGAAATTGCTGGAGAA
AGGTGCAAAGAACTGAAGAAGTTCATGGAGTAAAGGTTAATAAGCGATGCAGTGTATGAATAACTCAATAACGATAATAACTTCTT
GGAGCATATGAGGAGCTTTTTACATTTGTATTATGAATGCTTTGTATTGCAAGGTTTCGACTGCATACATTTGTGTTGTGTGTTAT
GAATGCTTTATATTGCAAAGGTTTCGATCATTACTAAAGGATCGGAATTTGGTGATGCCATAGTTTGTGCCTTTAGTGCTTATTTG
TCATGTAGGGTTTATCTTCAAAATCTTGCTTAACATTTTATAACAGCTAGAAAAACCAAACTTTTGACTT
>TvGPX6 Triphysaria versicolor Tv.c6882_g2_i2.4631

CGCGTTACAACTTAGTCCATTAGTTCTAACAAAAAAAACTTGTGATCAAAAGCTTACTACTGTCTCCCCCATTTCAATACACTGCG
TAGTTATCCAACTTCCTTGTTTTCTCGATCGATCAAAGCTAGATTTGAATATAAGATGGCCAGCCAGTCCAGCACGCCCCAATCAA
TCCATGATTTCACTGTCAAGGATGCTAAGGGTAATGATGTAAATCTGGGTATCTACAAGGGCAAAGTCTTGTTGATTGTCAATGTT
GCTTCACAGTGTGGCTTAACCAATTCAAATTACACTGAGTTGACCCAACTATATGACAAATACAAGGGCCAAGGATTGGAGATTTT
GGCATTTCCTTGCAATCAGTTTGGCTCACAAGAGCCCGGCTCCAATGAAGAAATTCAGGAATTTGTCTGCACTCGTTTTAAGGCCG
AGTATCCCGTATTTGACAAGGTTGATGTAAATGGGCCGAATAGTGCTCCGATATACAAGTACATGAAGTCGGCCAAAGGCAGCATT
TTCGGAGACGGTATTAAATGGAACTTCTCGAAGTTCCTTGTCGATAAAGAGGGTCGTGTTGTTGATCGCTATGCTCCCACCACATC
TCCTCTAAGCATCGAGAAGGATATTAAGAAGCTCCTTCAGAAGGCTTAAGAATGGGTATTAATAAGCTCCTATACCGTTTGGTCCA
TAGCTATAAAAGAATAAACCCTCGTTGAGGATGTTTGTATTAATAATCGTGTGAAACGTACAAGACCTTGTTATTTTGCATCGTGT
TCGAAGAATATAGGTCATACTTCCGTGGGAATTTATCGTATTTATTGTTTGTTTCCCATGCTCGATCTTTATGAAAACTATGGTTT
TATATTTGTTCTTTAAATATCCCGGAATGTCTGATTTTCTTGTTTAGTAATTTTTTCAGTAATTGAGAAAACTGTGTTTCGTGTTA
CAAAGTGGGGCATATAATTTAACTAAAAGTAAATAAAACTACAAACAAG

>ShGPX8 Striga hermonthica Sh.c14473_g1_i1.4564
GTAGAGATGGGTATGAGCCTTGTTGCGTCTGGTGGGTCCCATGGCGGTATCGAGACCGTGAAGAAGAAGAGGGGTAGGCCGAGGAA
GTACGGGCCCGAAGGGCCTCCGCCTAAGGTGTCTTTGAAACTCTCGTCGCCTGTTCCAAAAACGCCTTCGTCGTCTGATCCGAACA
CTTCGGGGGAGAAAGCAAGACGAGGGAGGCCGCCCGGGACCGGGTGGAAACAGAAACTTGCTCCTCTTGGCGATTGGATGAATAGC
TCAGCTGGACAGGCTTTTACCCCCCATGTTCTTCACATAGGAACTGGAGAGGATATTTCAGCAAAAATATTGGCATTTGCGCAACA
GAGATCGAGAGCTTTATGCATAATGTCAGGAAGTGGATCGGTTTCTGTAGTGACACTAAAGCAACCTGCAAATCCTTCTAGCACCG
TAACTTTTGAGGGTCGATTTGAGATTCTATGCCTGTCTGGTTCTTACTTGGTAGCTGAGAATGGTGGTCCCCACACTCGAACTGGT
GGTATTAGCATATCTGTGTGTAATCCTGATGGCCATATGCTTGGGGGTTCAATAGGCGGCCGACTTATTGCAGCAAATAACGTGCA
GGTGGTGGGGTGCAGCTTTGTGTACGATGCTACAAAGGCAAAGACCAAACCCGAGTCCCAAATAACGAACGAAAGGAACCTTCTAG
AACAATCTTCTGAAGAGGCGTACACCCCGTATAGTGCTGCCGCCTCTAACCAAAACCCTAATGTGGGGCCGAGTGCTTGGCAGCTA
AGTTCACGACAAGACATAAAGAGATCACAAGAGGATATTGACCTGACTCGCGGATGATTTATGATGATAAAATGTGCTATTATGAA
TTCTCTGTATATATGATGGAAAACGAGAAAATGTTCGAGTGTTCGAGTTGTGCTAACGTATTCTTGTAACGAAATTCAGTCTGTAG
GATCTTAAGAAAGTAGGAGAAAGTAGTAGAGTTTGATATAGTCGGTTGAAGTTTAAGCAAGGATGTATTGTTCTAAATTTCTAATA
GGTGTAAGTGATGTTGTATTAATGGATTGTATTGTAAAAAACATTGTGGGATTTATTCATTTTCAGTTGGAAACACTAGTTGTGCA
TAGCTTGACCATATATTGTTAACTTGAAAAATTTTAGACAGAGGGTAGAAATTCTTCATAACTAGAATTGAAAATTAGGGGAACTG
AGTTCAAAATTATTACAAGAAATCCAACACAATTATAATTACAACAAGTCTCAATCTGCTAGCCATTTAATATATCATGCAAGATA
ACCGACCAGTCGAGCATCAATAGGACCCGAGGTTGCAAGGGTTATGAAAAAAACACGTTTTTTCGTTTACGACCCCAATAGCTTTA
CGATATCCCTCTCGATCGTGAGAGGTGAGGTCGTAGGGTAATAACGATCAACAGGCTGCCCATTCTTATCTACCAAAAACTTTGCA
AAATTCCACTGGACATCATCCCCAAAGATTCCCCATTTGCCCGATTTTAAGAACTTGTACAGTGGAGCAGCATTATCACCATTCAC
CTCAATCTTAGCAAAGATGGGGAAATCCGACTTGAATCGAGTGCAAACAAAGTCGAGAATTTGATCATTGCTTCCAGGTTCTTCTT
CACCAAACTGGTTGCATGGGAAAGCTAAAATCTCGAGGCCTTGATCTCTGAACTTTTCATACAGCTTATTTAGCTCGGTATAGTTT
GAATTTGTCATCCCACATTTTGAAGCAACATTGACTATCAACAACACTTTGCCTCTGTAAGTGCTTAGCTCCACATCACCTCCATT
AGCATCCTTGACAGTGAAATCGAATACAGACTGAGGCGACGCCATTTGTTTGAATTCGTGTTTTCGGAGAGCTAAAGAAGAATGAG
ATAGTGGTAGTGTATCTCGTGAGGGCCATAGCTAAGTACATAG
>TvGPX8 Triphysaria versicolor Tv.c6531_g1_i1.4491
CATTCGTAAAATGATCATCCAGTAACGAACATAAAATGGTAAATATCAAAGAACATTGAGTTCAAATACATTTAAAATACATTTTA
AAAGAAAAGTGGAGTATCTCATGTGTTCCCCGATATTATTTCATTTTTTTACAACATAAGGAATCAGATTTTCTATTCCAACTCGA
ATCAATCAAAGTCCAATGGTTATCGCAGTGACAAAATAGAATAAAAACTGCTGCCCATGGAAATAAATTCGACATGTTATATAACA
ATAGTACAATAAAGGTTCAACAGTACCGGTTATGAGAGCTCCAGGAGCTTTCGAATATCCCTCTCAATTGTAAGAGGCGATGTCGT
GGGGTAATAGCGATCGACAGGCTGACCATTCTTATCAACCAAAAACTTGGCAAAATTCCACTGGATGTCATCGCCAAAGATTCCCC
ACTTTCCCGACTTCAAGAACTTGTACAGTGGAGCAGCATTATCACCATTCACTTCGATCTTACCGAAGATGGGAAAATCTGACTTG
AATCGAGCGCAAACAAAATCAAGAATTTGATCATTGTTTCCAGGTTCTTCTTCGCCAAACTGATTGCATGGAAAAGCCAGAATCTC
GAGGCCTTGATCTTTGTACTTTTCATACAGGTGATTCAGCTCAGTGTAGTTCGAGTTAGTCATCCCACATTTGGAGGCAACATTGA
CAATGAGCAACAGTTTGCCTCTATAAATGCTTAGATCCACATCATCTCCTTTAGCATCCTTGACGGCGACATCAAACACTGATTGC
GGCGATGCCATTTGATTAAAGTGAGTCCG
